# Regioselective Radical Cascade Cyclizations of Alkyne-Tethered Cyclohexadienones with Chalcogenides under Visible-Light Catalysis

**DOI:** 10.1021/acsomega.3c03362

**Published:** 2023-09-20

**Authors:** Vadla Shiva Prasad, Vadithya Ranga Rao, Maram Gangadhar, Sunil Kumar Nechipadappu, Praveen Reddy Adiyala

**Affiliations:** † Department of Organic Synthesis and Process Chemistry, CSIR-Indian Institute of Chemical Technology, Hyderabad 500007, Telangana, India; ‡ Laboratory of X-Ray Crystallography, Department of Analytical Chemistry, CSIR-Indian Institute of Chemical Technology (CSIR-IICT), Hyderabad 500 007, India; § Academy of Scientific and Innovative Research (AcSIR), Ghaziabad 201002, India

## Abstract

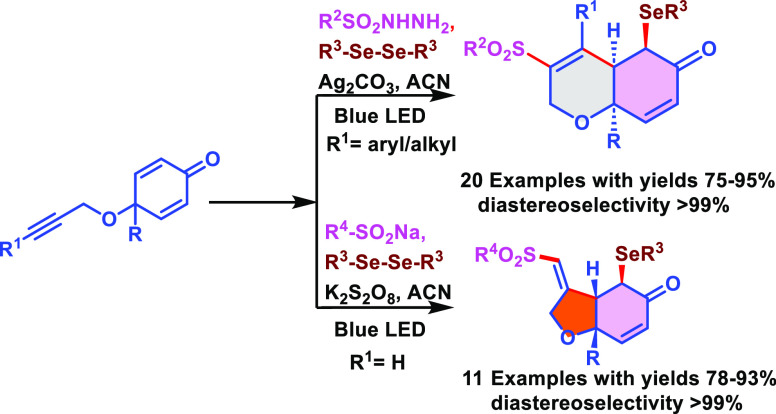

Herein, we demonstrated a silver/K_2_S_2_O_8_-mediated highly regio- and diastereoselective 6/5-*exo* trig radical cascade cyclization of alkyne-tethered cyclohexadienones with sulfonyl hydrazides or sodium sulfinates and subsequent selenation to access 6,6-dihydrochromenone and 6,5-fused tetrahydro benzofuranone derivatives. This reaction protocol features high functional group compatibility and has a wide substrate scope providing a variety of dihydrochromenones and tetrahydro benzofuranone derivatives in good to excellent yields. The reaction proceeds via the attack of a sulfonyl radical to alkyne over the activated Michael acceptor. The TEMPO quenching experiment implies the presence of a radical intermediate. Further synthetic versatility of 6,6- and 5,6-fused derivatives is also showcased.

## Introduction

Organochalcogen compounds,^1^ particularly containing sulfonyl^2^ (R^1^SO^2^−) and seleno^3^ (R^2^Se−) groups, are highly versatile functional groups that are widely present in natural products and^4^ pharmaceuticals and play a substantial role in organic synthesis (nucleophiles, electrophiles, ligands, and catalysts), biochemistry,^5^ medicinal chemistry,^6^ and materials science.^7^ Additionally, molecules containing selenium and sulfone have been widely used in organic synthesis.^8^ The C–S and C–Se bonds are weak, making it simple to add, delete, and change them into different functional groups. The incorporation of chalcogen functionality into a single molecule might provide an opportunity to enhance the bioactivity of the drugs or original lead compounds.^9^ Therefore, the discovery of novel techniques for incorporating both seleno and sulfonyl groups into a single molecule is of enormous synthetic significance, enabling a rapid increase in molecular complexity.

**Scheme 1:**
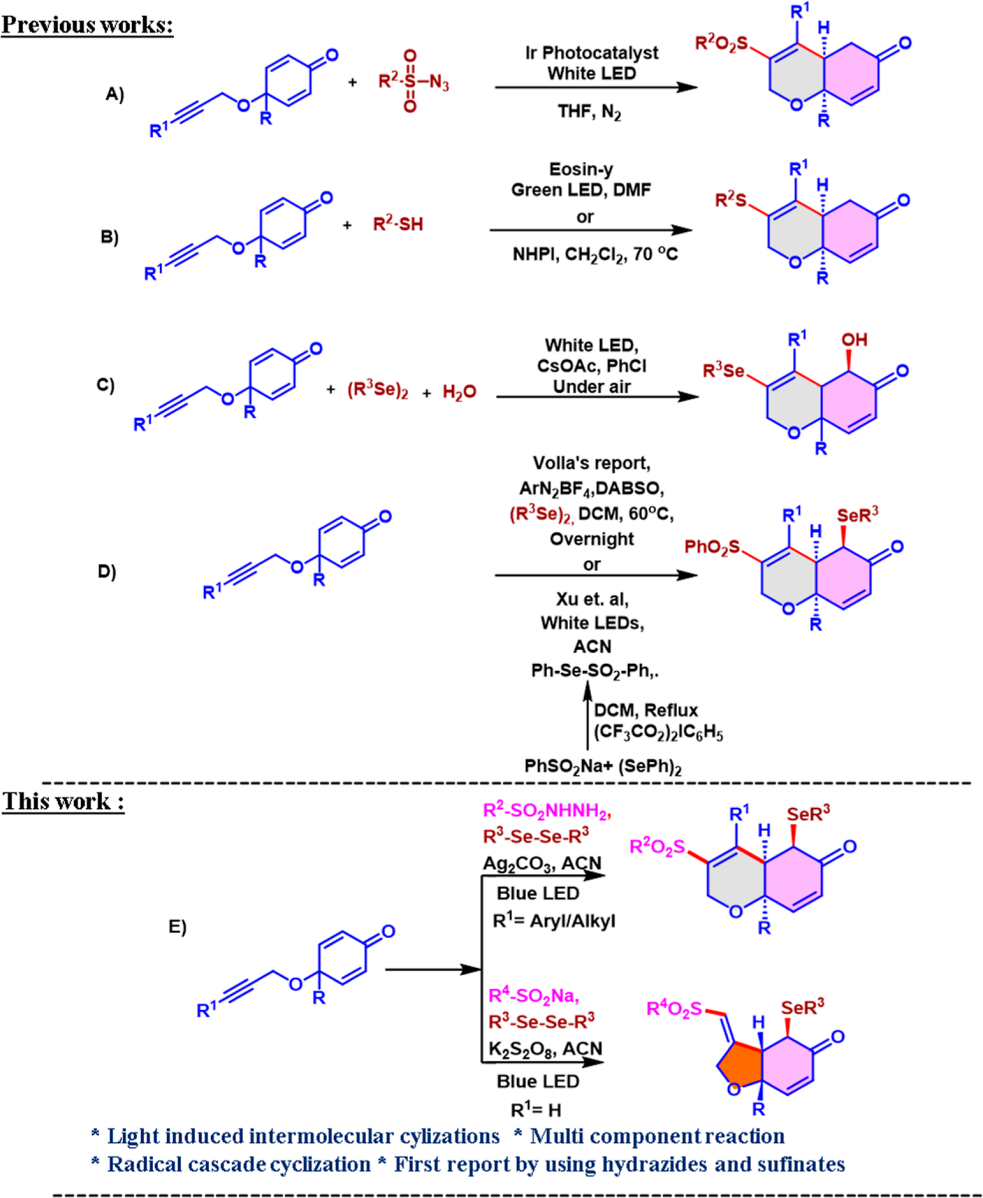
Comparative Approaches for Cascade Cyclizations of Alkyne-Tethered Cyclohexadienones between the Reported Work and This Work

Visible-light photocatalysis^10^ has emerged as a key method for stimulating a variety of synthetic conversions for the creation of C–X and C–C bonds in the quest for energy-efficient and sustainable synthesis. For the production of radical ion intermediates under benign conditions, visible light-induced catalysis has proven to be one of the most reliable methods. On the other hand, the radical cascade cyclizations^11^ are privileged strategies that have been rapidly applied for the manufacture of complex molecular skeletons from commercially available acyclic substrates. In particular, the cascade cyclizations of 1,6-enynes^12^ have drawn a lot of attention for manufacturing of cyclic complex ring systems from acyclic moieties. Alkyne-tethered cyclohexadienones are used in manufacturing cyclic complex ring systems from acyclic moieties. Alkyne-tethered cyclohexadienones, which can be obtained by dearomatizing the appropriate phenols, are annulated in a variety of ways and are able to generate novel cyclic structural entities. In this context, the production of fused carbocycles from alkyne-tethered cyclohexadienones has received substantial research attention.^13^ As a result, great efforts have been made to synthesize these fused carbocycle motifs in a simple and effective synthetic approach. Although several strategies have been developed to construct decorated bicyclic frameworks via cyclizations of cyclohexadienones, recognized as powerful strategies, these structural entities are widely found in bioactive natural products. For instance, metal catalysts including Rh,^14^ Pd,^15^ Cu,^16^ and Ni^17^ have been widely studied in the synthesis of 5- and 6-fused carbocycles. There are only a few reports on the radical cascade cyclization of alkyne-tethered cyclohexadienones for the formation of 6,5- and 6,6-fused carbocycles. In a pioneering report, Lam and co-workers developed a radical cascade cyclization of alkyne-tethered cyclohexadienones with sulfonyl radicals produced from sulfonyl azides (Scheme 1,A)^18^ under visible light-induced iridium photocatalysis. Volla and co-workers demonstrated a Giese-type cyclization protocol for the highly diastereoselective synthesis of sulfenylated dihydrochromenones under visible-light irradiation (Scheme 1,B).^19^ Sahoo’s group also discovered a novel protocol for the construction of 3-thioaryl-bearing fused dihydrochromenones via regio- and chemoselective radical cyclization under *N*-hydroxyphthalimide (NHPI) catalysis (Scheme 1,B).^20^ Later, Xu developed a three-component tandem cyclization/substitution for the construction of highly substituted dihydrochromenones using cyclohexadienones, diselenides, and H_2_O (Scheme 1,C).^21^ Xu and colleagues reported the cascade cyclization of alkyne-tethered cyclohexadienones, although they employed a presynthesized starting material where the preparation required reflux temperature and a pricey hypervalent reagent (Scheme 1,D).^22^ Very recently, while this manuscript was under preparation, Volla and co-workers also demonstrated a multicomponent radical cascade reaction for the synthesis of highly functionalized dihydrochromenones using explosive aryldiazonium salts as aryl partners and expensive DABSO as a SO_2_ source and also as a redox mediator under reflux conditions (Scheme 1,D).^22,23^

Inspired by these results and our previous experience on photoredox catalysis,^24^ we envisioned that sulfonyl radicals generated from sulfonyl hydrazides or sodium sulfinates might be added to alkynyl cyclohexadienones and undergo Giese-type cyclization, followed by selenation by diselenides to produce bis-functionalization (Scheme 1,E) (see Scheme 1).

## Results and Discussion

**Scheme 2:**
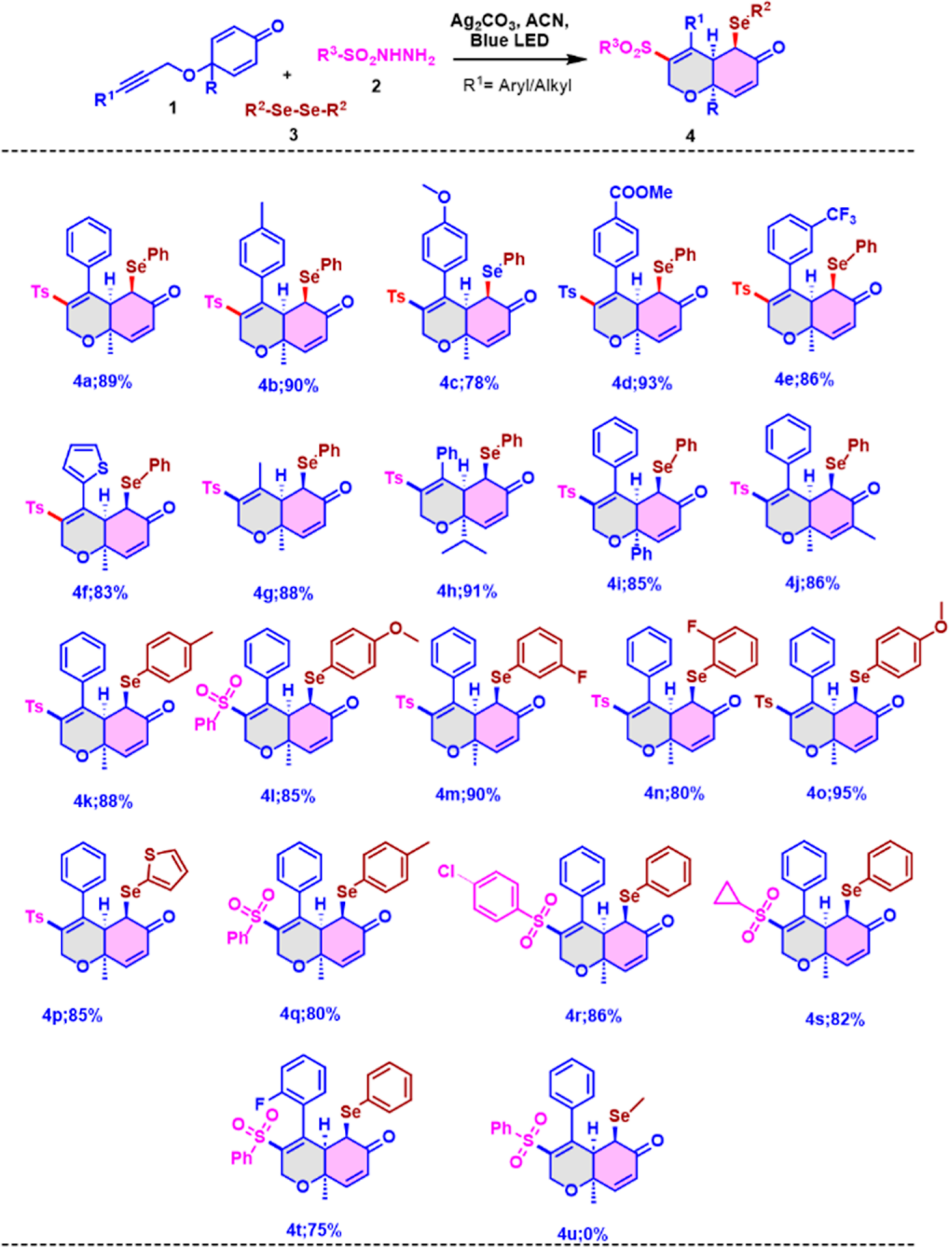
Substrate Scope of 6-*Exo* Trig Radical Cascade Cyclization of Cyclohexadienones Standard Condition for 6-*Exo* Trig Cyclization: Internal alkyne (1.0 equiv), hydrazide (1.0 equiv), diphenyl diselenide (0.5 equiv), and Ag_2_CO_3_ (1.0 equiv) were mixed in 2 mL ACN under an inert atmosphere with 5 W blue LED irradiation.

To test the above hypothesis, we commenced our study to investigate the optimal reaction conditions (see Supporting Information Table S1). Under the optimized reaction conditions in hand, we turned our attention to probing the versatility of the developed protocol by independently screening with various cyclohexadienones, sulfonyl sources, and diaryl chalcogens (Scheme 2). First, the electronic effect of substituent R^1^ on the cyclohexadienones was investigated. Gratifyingly, both electron-withdrawing and -donating substituted arenes at the alkyne terminus of cyclohexadienones were well tolerated in this transformation and afforded the corresponding 6-*exo* trig cyclized products in good to excellent yields and with high diastereoselectivity, which indicated that electronic effects are not much effective in this protocol (Scheme 2,**4a–e**). We then examined the reaction efficiency with the replacement of arene at the alkyne terminus of cyclohexadienones with hetero aryl as well as alkyl substituent under the standard reaction conditions; the reaction proceeded well to afford the corresponding desired products in excellent yields (Scheme 2, **4f**–**g**). Changing the substituent in the quaternary carbon of cyclohexadienones from methyl to *iso*-propyl or phenyl made the 6-*exo* trig transformation proceed smoothly and gave the desired products in good yields (Scheme 2, **4h–i**). The radical cascade cyclization is also feasible for the cyclohexadienone having a methyl moiety at the α-position of carbonyl, affording the 6-*exo* trig cyclized product in an excellent yield (Scheme 2, **4j**). Encouraged by these results, we were devoted to searching for the versatility of this present protocol to further extend the substrate scope with respect to diaryldiselenides.

**Figure 1:**
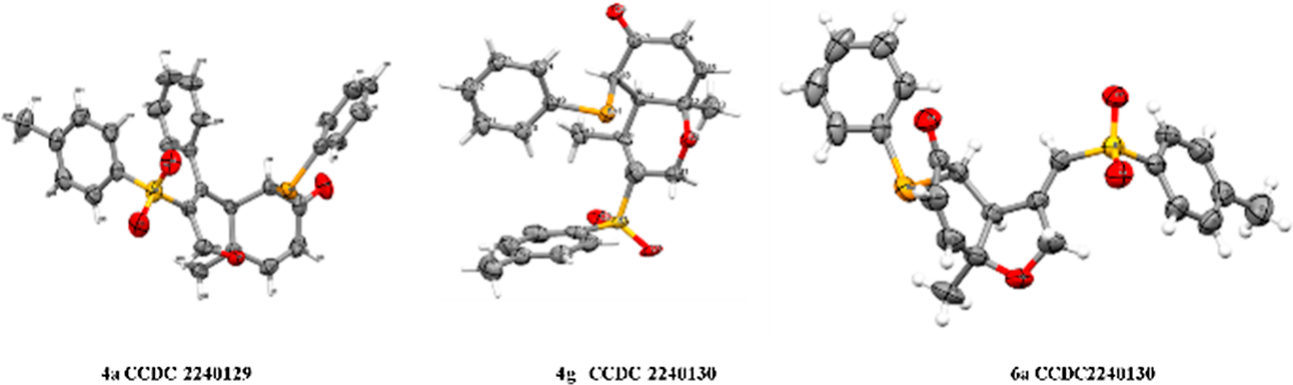
Crystal X-ray diffraction analysis of **4a**, **4g**, and **6a**.

**Scheme 3:**
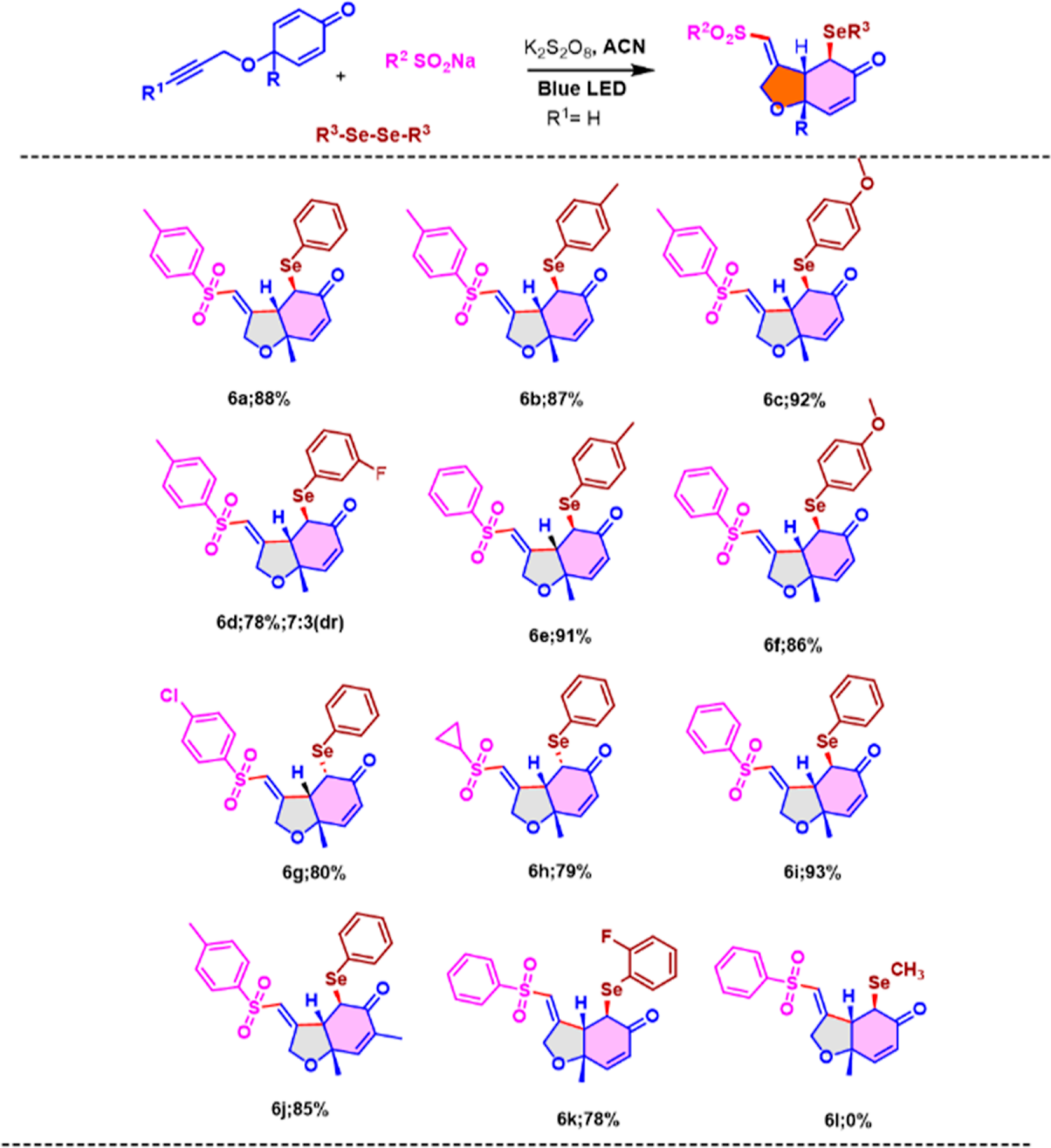
Substrate Scope of 5-*Exo* Trig Radical Cascade Cyclization of Cyclohexadienones Standard Condition for 5-*Exo* Trig Cyclization: Terminal alkyne (1.0 equiv), sodium phenyl sulfinate (3.0 equiv), diphenyldiselenide (0.5 equiv), and K_2_S_2_O_8_ (2.0 equiv) were mixed in 2 mL of ACN under an inert atmosphere with 5 W blue LED irradiation.

**Scheme 4:**
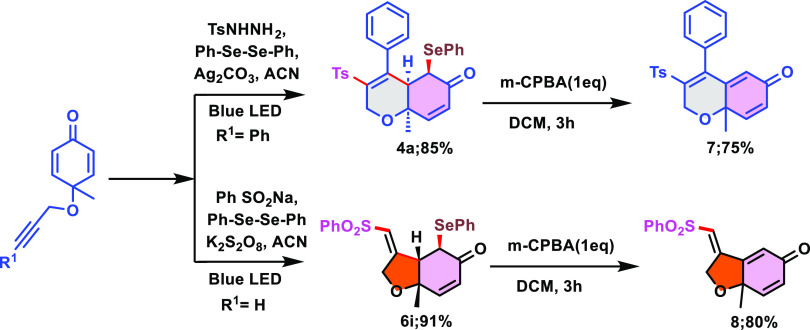
Gram-Scale Reaction and Synthetic Utility of 6/5-*Exo* Trig Radical Cascade Cyclization of Cyclohexadienones

Delectably, electron-withdrawing and -donating groups were well tolerated at the ortho, para, and meta positions of the phenyl ring of diaryl diselenides, affording the desired cyclized products in good to excellent yields (Scheme 2, **4k–o**). Notably, heteroaromatic-substituted diaryl diselenides underwent this transformation and afforded the corresponding 6-*exo* trig product in a good yield (Scheme 2, **4p**). Furthermore, various sulfonyl hydrazides such as 4-methylphenyl and cyclopropyl sulfonyl hydrazides were also compatible with this catalytic system, leading to the expected 6-*exo* trig cyclized products **4r** in excellent yields (Scheme 2, **4q–s**). We also further examined the performance of reaction efficiency with ortho-substituted arene at the alkyne terminus of cyclohexadienone, and it was observed that the formation of the desired product in a good yield (Scheme 2, **4t**), and it indicated there is no steric effect on this transformation. The addition of the aryl sulfonyl group occurred from the less sterically hindered *exo* side, and it is important to note that all of the products were produced as single diastereomers. Crystal X-ray diffraction analysis of **4a** and **4g** (Figure 1) indicates a clear relationship between all the stereocenters. However, the aliphatic selenides fail to give the desired product due to the low stability of the methyl selenide radical (Scheme 2, **4u**). Next, we then investigated the scope of developed radical cascade cyclization with a variety of cyclohexadienones having terminal alkynes, sodium arylsulfinates, and diaryldiselenides in the presence of K_2_S_2_O_8_ under blue LED light irradiation (see Supporting Information Table S1). Our assumption was expected to be a 6-*exo*-trig cyclized product similar to internal alkynes, but the reaction surprisingly afforded the 5-*exo*-trig cyclized product as depicted in Scheme 3. The absolute structure of **6a** was further confirmed with crystal X-ray diffraction analysis (Figure 1). Next, we explored the substrate scope with respect to various diaryl diselenides. Both electron donating as well as withdrawing groups bearing the phenyl ring of diaryl diselenides underwent this 5-*exo*-trig cyclization and we observed the desired products in good to excellent yields as well as excellent diastereoselective (Scheme 3, **6a–f**). Notably, meta halo substituted diaryl diselenides gave the product **6d** in good yield (78%) with a dr of 7:3.

Later, sodium arylsulfinates having functionalities such as 4-chlorophenyl and cyclopropyl were also tested to check the reaction efficiency, and the reaction proceeded well to afford the desired 5-*exo*-trig products in excellent yields (Schemes 3, **6g–h**). The reaction also proceeded with cyclohexadienone having a methyl moiety at the α-position of carbonyl, affording the 5-*exo*-trig-cyclized product in an excellent yield (Scheme 3, **6j**). Notably, 1,2-bis(2-fluorophenyl)diselane underwent 5-*exo* trig transformation smoothly and afforded the desired product in a good yield (Schemes 3, **6k**). Aliphatic selenide failed to give the desired product (Schemes 3, 6**l**).

To demonstrate the synthetic utility further, a gram-scale reaction for the synthesis of **4a** and **6i** is carried out, as illustrated in Scheme 4. Internal alkyne (0.500 g, 1.0 equiv) and terminal alkyne (0.500 g, 1.0 equiv) were both subjected to this transformation and afforded the required products **4a** and **6i** in 85 and 91% yields, respectively. When chalcogenated dihydrochromenones **4a** and tetrahydrobenzofuranones **6i** were individually treated to react with *m*-CPBA, they generated deselenated products 7 and 8 in 75 and 80% yields, respectively (please see the Supporting Information).

**Scheme 5:**
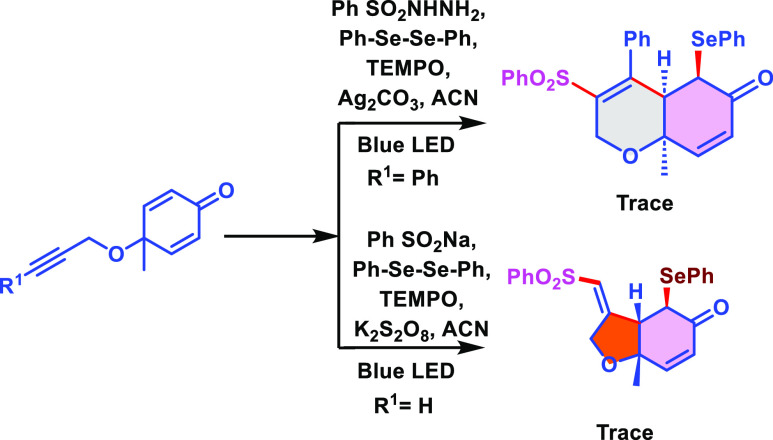
Control Experiments

The reaction was carried out in the presence of the radical scavenger TEMPO (1.0 equiv) under optimal reaction circumstances to ensure the involvement of the radical pathway for both 5/6-*exo* trig cyclizations (Scheme 5). A trace amount of the desired product was observed. This result indicates that this reaction possibly proceeds through a radical pathway.

**Scheme 6:**
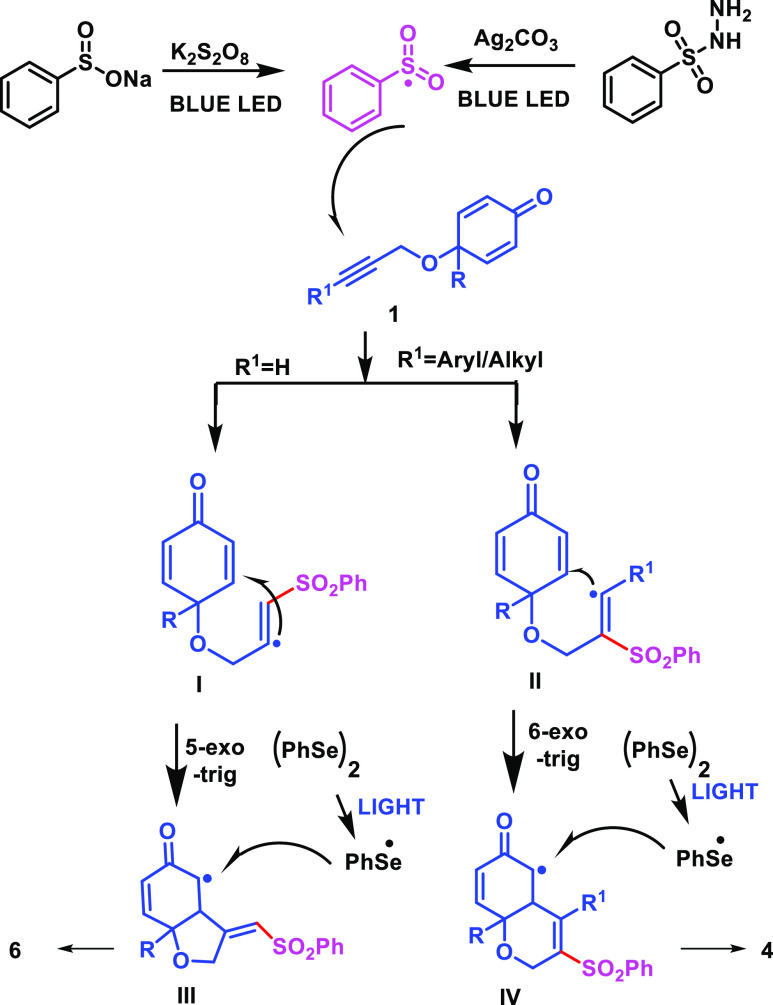
Plausible Mechanism

Based on the control experiments and previous literature,^25^ a plausible mechanism of the present method for 6/5-*exo*-trig cyclization is shown in Scheme 6.

Initially, the interaction of sulfonyl hydrazide with Ag_2_CO_3_ produced the highly active species Ts^•^. The sulfonyl radical species was added to the internal alkyne of cyclohexadienones to give alkenyl radical **II**, which underwent an intramolecular Giese-type 6-*exo* trig cyclization, furnishing the α-carbonyl radical intermediate **IV**. Furthermore, the stereoselective attack of the aryl selenyl radical onto the intermediate **IV** generated the final product 4 (The stereoselective attack of aryl selenium is explained in the Supporting Information). Similarly, in the case of terminal alkyne-tethered cyclohexadienones, sulfonyl radicals were generated from sodium sulfonates, and further, this sulfonyl radical species was added to the terminal alkyne of cyclohexadienones to give alkenyl radical **I**, which underwent an intramolecular Giese-type 5-*exo* trig cyclization, furnishing the α-carbonyl radical intermediate **III**. Further, the radical cross-coupling of intermediate **III** and the aryl selenyl radical that was formed by homolytic cleavage of diaryl diselenide in the presence of light generated the final product 6.

In conclusion, highly functionalized fused chalcogenated dihydrochromenones and tetrahydrobenzofuranones have been developed via silver/K_2_S_2_O_8_-mediated highly regio- and diastereoselective 6/5 *exo*-trig radical cascade cyclization of alkyne-tethered cyclohexadienones sulfonylhydrazides/arylsulfinates and diselenides under visible-light irradiation. The reaction displayed good functional group tolerance for a wide variety of functional groups and proceeded under the radical mechanism and mild conditions, employing cheap and easily available Ag_2_CO_3_ and K_2_S_2_O_8_ as an additive and an oxidant, respectively. Initial mechanistic research supported a radical route. In addition, we also demonstrated the deselenation of dihydrochromenones and tetrahydrobenzofuranones using *m*-CPBA. Further studies on the enantiomeric radical cascade cyclization under visible-light catalysis are underway in our laboratory and will be disclosed in due course.

## Experimental Section

### General Information

Unless otherwise noted, all the commercial materials were used without further purification. All reactions were performed under an inert atmosphere and in oven-dried glassware with magnetic stirring. All solvents were dried before use following the standard procedures. Reactions were monitored by TLC on precoated silica gel 60 F-254. TLC plates were visualized with UV light (254 nm), iodine treatment, or *p*-anisaldehyde stain. Column chromatography was carried out using silica gel (60–120 and 100–200 mesh) packed in glass columns. NMR spectra were recorded at 300, 400, and 500 MHz (H) and at 75, 100, and 125 MHz (C), respectively. Chemical shifts (δ) are reported in ppm, using the residual solvent peak in CDCl_3_ (H: δ = 7.26 and C: δ = 77.00 ppm) as the internal standard, and coupling constants (*J*) are given in Hz. HRMS were recorded using ESI-TOF techniques.

### General Procedure for the Cascade 6-*Exo* Trig Cyclization of Alkyne-Tethered Cyclohexadienones (i)

In an oven-dried glass vial, internal alkyne (0.05 g, 1.0 equiv), hydrazide (0.039 g, 1.0 equiv), diphenyldiselenide (0.032 g, 0.5 equiv), and Ag_2_CO_3_ (0.057 g, 1.0 equiv) were dissolved in 2 mL ACN; the vial was then sealed with a PTFE septum and purged with argon. The reaction mixture was stirred continuously for 6 h while being exposed to 5 W blue LED light at room temperature. Then, the reaction mixture was diluted with water. It was extracted with ethyl acetate (3 × 10 mL). Then, to the organic layer was added Na_2_SO_4_, and the mixture was concentrated under reduced pressure. The residue was directly subjected to flash chromatography on silica gel (hexane/ethyl acetate) to afford the desired product.

### General Procedure for the Cascade 5-*Exo* Trig Cyclization of Alkyne-Tethered Cyclohexadienones (ii)

In an oven-dried glass vial, terminal alkyne (0.050 g, 1.0 equiv), sodium arylsulfinates (0.151 g, 3.0 equiv), diphenyl diselenide (0.048 g, 0.5 equiv), and K_2_S_2_O_8_ (0.166 g, 2.0 equiv) were dissolved in 2 mL ACN; the vial was then sealed with a PTFE septum and purged with argon. The reaction mixture was stirred continuously for 6 h while being exposed to 5 W blue LED light at room temperature. Then, the reaction mixture was diluted with water. It was extracted with ethyl acetate (3 × 10 mL). Then, to the organic layer was added Na_2_SO_4_, and the mixture was concentrated under reduced pressure. The residue was directly subjected to flash chromatography on silica gel (hexane/ethyl acetate) to afford the desired product.

### Characterization Data

#### (4a*R*,5*R*,8a*R*)-8a-Methyl-4-phenyl-5-(phenylselanyl)-3-tosyl-4a,8a-dihydro-2*H*-chromen-6(5*H*)-one (**4a**)

The title compound was prepared according to the general procedure as described above (i) in an 89% (102 mg) yield; it was purified by column chromatography (20% EtOAc/hexane; R_*f*_ = 0.2) to afford **4a** as a white solid (mp: 195–200 °C). ^**1**^**H NMR (400 MHz, CDCl**_**3**_**)**: δ 7.41–7.39 (m, 2H), 7.36 (d, *J* = 8.3 Hz, 2H), 7.29 (dd, *J* = 12.7, 5.3 Hz, 2H), 7.26–7.23 (m, 2H), 7.21 (dd, *J* = 7.4, 4.0 Hz, 3H), 7.18–7.14 (m, 3H), 6.54 (d, *J* = 10.2 Hz, 1H), 6.09 (dd, *J* = 10.2, 1.3 Hz, 1H), 4.94 (d, *J* = 17.6 Hz, 1H), 4.66 (dd, *J* = 17.6, 2.5 Hz, 1H), 3.40 (dd, *J* = 4.2, 1.2 Hz, 1H), 2.85 (dd, *J* = 3.9, 2.2 Hz, 1H), 2.40 (s, 3H), 1.46 (s, 3H). ^**13**^**C NMR (100 MHz, CDCl**_**3**_**)**: δ 193.6, 146.3, 145.4, 144.2, 138.3, 138.1, 135.5, 134.0, 129.4, 129.1, 128.7, 128.3, 128.0, 127.64, 127.5, 68.4, 61.3, 48.4, 47.4, 22.6, 21.6. **IR (neat)**ν_max_: 3608, 1677, 1312, 1146, 751. **HRMS (ESI-TOF)**: *m*/*z* calcd for C_29_H_27_O_4_SSe [M + H]^+^, 551.07726; found, 551.07898.

#### (4a*R*,5*R*,8a*R*)-8a-Methyl-5-(phenylselanyl)-4-(*p*-tolyl)-3-tosyl-4a,8a-dihydro-2*H*-chromen-6(5*H*)-one (**4b**)

The title compound was prepared according to the general procedure as described above (i) in a 90% (100 mg) yield; it was purified by column chromatography (20% EtOAc/hexane; R_*f*_ = 0.2) to afford **4b** as a white solid (mp: 177–182 °C). ^**1**^**H NMR (400 MHz, CDCl**_**3**_**)**: δ 7.38 (d, *J* = 8.0 Hz, 4H), 7.26–7.18 (m, 3H), 7.15 (d, *J* = 8.1 Hz, 2H), 7.00 (d, *J* = 7.9 Hz, 2H), 6.90 (s, 2H), 6.51 (d, *J* = 10.2 Hz, 1H), 6.07 (dd, *J* = 10.2, 0.8 Hz, 1H), 4.90 (d, *J* = 17.5 Hz, 1H), 4.62 (dd, *J* = 17.6, 2.3 Hz, 1H), 3.39 (d, *J* = 4.0 Hz, 1H), 2.83–2.81 (m, 1H), 2.40 (s, 3H), 2.33 (s, 3H), 1.43 (s, 3H). ^**13**^**C NMR (125 MHz, CDCl**_**3**_**)**: δ 193.6, 146.3, 145.7, 144.2, 138.7, 137.9, 134.0, 132.5, 130.3, 129.3, 129.0, 128.6, 128.2, 127.6, 127.4, 68.4, 61.3, 48.5, 47.5, 22.6, 21.6, 21.3. **IR (neat)**ν_max_: 3606, 3033, 2975, 1682, 1316, 1152, 1082, 755. **HRMS (ESI-TOF)**: *m*/*z* calcd for C_30_H_29_O_4_SSe [M + H]^+^, 565.0926; found, 565.0946.

#### (4a*R*,5*R*,8a*R*)-4-(4-Methoxyphenyl)-8a-methyl-5-(phenylselanyl)-3-tosyl-4a,8a-dihydro-2*H*-chromen-6(5*H*)-one (**4c**)

The title compound was prepared according to the general procedure as described above (i) in a 78% (84 mg) yield; it was purified by column chromatography (20% EtOAc/hexane; R_*f*_ = 0.2) to afford **4c** as a white solid (mp: 188–193 °C). ^**1**^**H NMR (400 MHz, CDCl**_**3**_**)**: δ 7.38 (t, *J* = 7.8 Hz, 4H), 7.25–7.20 (m, 3H), 7.16 (d, *J* = 8.1 Hz, 2H), 6.95 (s, 2H), 6.73 (d, 2H), 6.53 (d, *J* = 10.2 Hz, 1H), 6.08 (dd, *J* = 10.2, 0.8 Hz, 1H), 4.94 (d, *J* = 17.6 Hz, 1H), 4.63 (dd, *J* = 17.6, 2.3 Hz, 1H), 3.81 (s, 3H), 3.37 (d, *J* = 3.3 Hz, 1H), 2.83 (d, *J* = 2.1 Hz, 1H), 2.40 (s, 3H), 1.44 (s, 3H). ^**13**^**C NMR (100 MHz, CDCl**_**3**_**)**: δ 193.64, 160.0, 146.3, 145.5, 144.1, 138.2, 138.2, 134.0, 130.3, 129.3, 129.1, 128.2, 127.7, 127.4, 113.4, 68.5, 61.4, 55.4, 48.5, 47.6, 22.6, 21.6. **IR (neat)**ν_max_: 3021, 2921, 1681, 1248, 753. **HRMS (ESI-TOF)**: *m*/*z* calcd for C_30_H_29_O_5_SSe [M + H]^+^, 581.0879; found, 581.0895.

#### Methyl 4-((4a*R*,5*R*,8a*R*)-8a-methyl-6-oxo-5-(phenylselanyl)-3-tosyl-4a,5,6,8a-tetrahydro-2*H*-chromen-4-yl)benzoate (**4d**)

The title compound was prepared according to the general procedure as described above (i) in a 93% (95 mg) yield; it was purified by column chromatography (20% EtOAc/hexane; R_*f*_ = 0.2) to afford **4d** as a white solid (mp:193–198 °C). ^**1**^**H NMR (500 MHz, CDCl**_**3**_**)**: δ 7.88 (d, *J* = 8.0 Hz, 2H), 7.42 (d, *J* = 8.2 Hz, 2H), 7.38–7.37(m, 2H), 7.28 (d, *J* = 7.2 Hz, 2H), 7.24 (d, *J* = 6.9 Hz, 2H), 7.21 (d, *J* = 8.1 Hz, 3H), 6.54 (d, *J* = 10.2 Hz, 1H), 6.10 (dd, *J* = 10.2, 1.0 Hz, 1H), 4.91 (d, *J* = 17.8 Hz, 1H), 4.65 (dd, *J* = 17.8, 2.4 Hz, 1H), 3.94 (s, 3H), 3.36 (d, *J* = 3.3 Hz, 1H), 2.85 (dd, *J* = 3.5, 2.5 Hz, 1H), 2.43 (s, 3H), 1.47 (s, 3H). ^**13**^**C NMR (100 MHz, CDCl**_**3**_**)**: δ 193.4, 166.4, 146.3, 144.8, 144.3, 140.4, 138.9, 137.8, 133.8, 130.3, 130.1, 129.6, 129.2, 128.4, 127.7, 127.5, 68.3, 61.3, 52.3, 48.2, 47.2, 22.6, 21.6. **IR (neat)**ν_max_: 3605, 1723, 1684, 1286, 1152, 760. **HRMS (ESI-TOF)**: *m*/*z* calcd for C_31_H_29_O_6_SSe [M + H]^+^, 609.0825; found, 609.0844.

#### (4a*R*,5*R*,8a*R*)-8a-Methyl-5-(phenylselanyl)-3-tosyl-4-(3-(trifluoromethyl)phenyl)-4a,8a-dihydro-2*H*-chromen-6(5*H*)-one (**4e**)

The title compound was prepared according to the general procedure(i) as described above (i) in an 86% (86 mg) yield; it was purified by column chromatography (20% EtOAc/hexane; R_*f*_ = 0.2) to afford **4e** as a white solid (mp: 203–208 °C). ^**1**^**H NMR (400 MHz, CDCl**_**3**_**)**: δ 7.56 (d, *J* = 7.6 Hz, 2H), 7.40 (dd, *J* = 18.8, 6.8 Hz, 5H), 7.29 (dt, *J* = 5.7, 1.9 Hz, 1H), 7.24 (d, *J* = 7.4 Hz, 2H), 7.23–7.17 (m, 3H), 6.56 (d, *J* = 10.2 Hz, 1H), 6.11 (dd, *J* = 10.2, 1.2 Hz, 1H), 4.98 (d, *J* = 17.9 Hz, 1H), 4.72 (d, *J* = 17.9 Hz, 1H), 3.33 (d, *J* = 3.5 Hz, 1H), 2.83–2.81 (m, 1H), 2.40 (s, 3H), 1.51 (s, 3H). ^**13**^**C NMR (100 MHz, CDCl**_**3**_**)**: δ 193.4, 146.2, 144.9, 143.5, 140.0, 137.8, 136.3, 134.0, 129.6, 129.2, 128.5, 127.6, 127.3, 125.6, 125.5, 68.3, 61.3, 48.2, 47.2, 22.7, 21.5. **IR (neat)**ν_max_: 3604, 1683, 1329, 1158, 1083, 756. **HRMS (ESI-TOF)**: *m*/*z* calcd for C_30_H_26_F_3_O_4_SSe [M + H]^+^, 619.0646; found, 619.0663.

#### (4a*R*,5*R*,8a*R*)-8a-Methyl-5-(phenylselanyl)-4-(thiophen-2-yl)-3-tosyl-4a,8a-dihydro-2*H*-chromen-6(5*H*)-one (**4f**)

The title compound was prepared according to the general procedure as described above (i) in an 83% (94 mg) yield; it was purified by column chromatography (20% EtOAc/hexane; R_*f*_ = 0.2) to afford **4f** as a white solid (mp: 198–203 °C). ^**1**^**H NMR (400 MHz, CDCl**_**3**_**)**: δ 7.37–7.31 (m, 4H), 7.24 (dd, *J* = 5.1, 1.1 Hz, 1H), 7.21–7.16 (m, 2H), 7.15–7.08 (m, 4H), 6.88 (dd, *J* = 5.0, 3.6 Hz, 1H), 6.46 (d, *J* = 10.2 Hz, 1H), 6.03 (dd, *J* = 10.2, 1.2 Hz, 1H), 4.87 (dd, *J* = 25.8, 13.5 Hz, 1H), 4.64–4.59 (m, 1H), 3.38 (d, *J* = 5.5 Hz, 1H), 2.19–2.78 (m, 1H), 2.32 (s, 3H), 1.37 (s, 3H). ^**13**^**C NMR (100 MHz, CDCl**_**3**_**)**: δ 193.4, 146.2, 144.3, 141.0, 138.9, 137.6, 135.1, 134.5, 131.4, 130.2, 129.3, 129.1, 128.4, 128.1, 127.7, 127.4, 126.8, 68.6, 61.8, 49.2, 48.2, 22.7, 21.6. **IR (neat)**ν_max_: 3615, 1682, 1315, 1152, 1082, 755. **HRMS (ESI-TOF)**: *m*/*z* calcd for C_27_H_25_O_4_S_2_Se [M + H]^+^, 557.0334; found, 557.0354.

#### (4a*R*,5*R*,8a*R*)-4,8a-Dimethyl-5-(phenylselanyl)-3-tosyl-4a,8a-dihydro-2*H*-chromen-6(5*H*)-one (**4g**)

The title compound was prepared according to the general procedure as described above (i) in an 88% (118 mg) yield; it was purified by column chromatography (20% EtOAc/hexane; R_*f*_ = 0.2) to afford **4g** as a white solid (mp: 184–188 °C). ^**1**^**H NMR (500 MHz, CDCl**_**3**_**)**: δ 7.89 (t, *J* = 6.5 Hz, 2H), 7.36 (d, *J* = 8.0 Hz, 2H), 7.30–7.26 (m, 1H), 7.22–7.18 (m, 4H), 6.50 (d, *J* = 10.2 Hz, 1H), 6.10 (dd, *J* = 10.2, 1.2 Hz, 1H), 4.64 (dd, *J* = 17.1, 1.8 Hz, 1H), 4.58–4.54 (m, 1H), 3.61 (dd, *J* = 4.3, 1.2 Hz, 1H), 2.64 (dd, *J* = 4.1, 1.7 Hz, 1H), 2.42 (s, 3H), 2.18 (t, *J* = 2.1 Hz, 3H), 1.38 (s, 3H). ^**13**^**C NMR (100 MHz, CDCl**_**3**_**)**: δ 193.77, 147.11, 144.69, 143.73, 138.37, 134.71, 129.89, 129.12, 128.58, 127.51, 127.40, 68.30, 61.50, 48.92, 47.43, 22.76, 21.66, 18.52. **IR (neat)**ν_max_: 3019, 2349, 1680, 1214, 1146, 743. **HRMS (ESI-TOF)**: *m*/*z* calcd for C_24_H_25_O_4_SSe [M + H]^+^, 489.0617; found, 489.0633.

#### (4a*R*,5*R*,8a*R*)-8a-Isopropyl-4-phenyl-5-(phenylselanyl)-3-tosyl-4a,8a-dihydro-2*H*-chromen-6(5*H*)-one (**4h**)

The title compound was prepared according to the general procedure as described above (i) in 91% (93 mg) yield; it was purified by column chromatography (20% EtOAc/hexane; R_*f*_ = 0.2) to afford **4h** as a white solid (mp: 188–192 °C). ^**1**^**H NMR (400 MHz, CDCl**_**3**_**)**: δ 7.41–7.37 (m, 4H), 7.30–7.23 (m, 3H), 7.21–7.15 (m, 5H), 6.89 (s, 2H), 6.63 (d, *J* = 10.5 Hz, 1H), 6.23 (dd, *J* = 10.5, 0.9 Hz, 1H), 4.88 (d, *J* = 17.7 Hz, 1H), 4.65 (dd, *J* = 17.7, 2.2 Hz, 1H), 3.47 (dd, *J* = 4.2, 1.2 Hz, 1H), 3.13 (dd, *J* = 3.9, 1.9 Hz, 1H), 2.40 (s, 3H), 2.33 (dd, *J* = 13.7, 6.8 Hz, 1H), 1.17 (d, *J* = 6.7 Hz, 3H), 0.85 (d, *J* = 6.9 Hz, 3H). ^**13**^**C NMR (125 MHz, CDCl**_**3**_**)**: δ 193.6, 144.9, 144.2, 141.7, 138.2, 138.1, 135.8, 133.8,130.4, 129.3, 129.0, 128.5, 128.2, 128.0, 127.5, 73.1, 61.2, 48.4, 45.0, 29.3, 21.6, 18.5, 15.7. **IR (neat)**ν_max_: 3357, 2925, 1677, 1145, 1022, 748. **HRMS (ESI-TOF)**: *m*/*z* calcd for C_31_H_31_O_4_SSe [M + H]^+^, 579.1082; found, 579.1102.

#### (4a*R*,5*R*,8a*R*)-4,8a-Diphenyl-5-(phenylselanyl)-3-tosyl-4a,8a-dihydro-2H-chromen-6(5*H*)-one (**4i**)

The title compound was prepared according to the general procedure as described above (i) in an 85% (86 mg) yield; It was purified by column chromatography (20% EtOAc/hexane; R_*f*_ = 0.2) to afford **4i** as a white solid (mp: 228–233 °C). ^**1**^**H NMR (500 MHz, CDCl**_**3**_**)**: δ 7.49–7.42(m, 8H), 7.33–7.21 (m, 7H), 6.93 (d, *J* = 8.2 Hz, 2H), 6.86 (d, *J* = 8.2 Hz, 2H), 6.63 (d, *J* = 10.2 Hz, 1H), 6.05 (d, *J* = 10.2 Hz, 1H), 4.94 (t, *J* = 15.1 Hz, 1H), 4.38–4.34 (m, 1H), 3.81 (s, 1H), 3.62 (d, *J* = 3.9 Hz, 1H), 2.32 (d, *J* = 24.0 Hz, 3H). ^**13**^**C NMR (100 MHz, CDCl**_**3**_**)**: δ 193.6, 147.1, 145.7, 143.7, 139.0, 138.7, 137.8, 135.3, 134.3, 129.4, 129.1, 129.0, 128.9, 128.8, 128.4, 128.1, 127.1, 126.9, 126.2, 72.9, 62.1, 48.3, 45.0, 21.5. **IR (neat)**ν_max_: 3452, 2980, 1680, 1316, 1150, 761. **HRMS (ESI-TOF)**: *m/z* calcd for C_34_H_29_O_4_SSe [M + H]^+^, 613.0929; found, 613.0946.

#### (4a*R*,5*R*,8a*R*)-7,8a-Dimethyl-4-phenyl-5-(phenylselanyl)-3-tosyl-4a,8a-dihydro-2*H*-chromen-6(5*H*)-one (**4j**)

The title compound was prepared according to the general procedure as described above (i) in an 86% (96 mg) yield; it was purified by column chromatography (20% EtOAc/hexane; R_*f*_ = 0.2) to afford **4j** as a white solid (mp: 118–122 °C). ^**1**^**H NMR (500 MHz, CDCl**_**3**_**)**: δ 7.30 (dd, *J* = 17.0, 7.4 Hz, 4H), 7.23–7.19 (m, 3H), 7.16 (d, *J* = 7.1 Hz, 2H), 7.15–7.10 (m, 3H), 7.07 (d, *J* = 7.7 Hz, 2H), 6.23 (s, 1H), 4.86 (d, *J* = 17.6 Hz, 1H), 4.56 (d, *J* = 17.5 Hz, 1H), 3.35 (d, *J* = 3.2 Hz, 1H), 2.79 (s, 1H), 2.33 (s, 3H), 1.80 (s, 3H), 1.37 (s, 3H). ^**13**^**C NMR (125 MHz, CDCl**_**3**_**)**: δ 194.1, 145.5, 144.2, 141.6, 138.3, 138.2, 135.7, 134.5, 134.0, 130.4, 129.3, 129.1, 128.7, 128.2,127.5, 68.9, 61.3, 48.6, 47.7, 22.9, 21.6, 16.3. **IR (neat) ν**_**max**_: 3340, 2950, 1676, 1315, 1151, 1097, 752. **HRMS (ESI-TOF)**: *m*/*z* calcd for C_30_H_29_O_4_SSe [M + H]^+^, 565.0927; found, 565.0946.

#### (4a*R*,5*R*,8a*R*)-8a-Methyl-4-phenyl-5-(*p*-tolylselanyl)-3-tosyl-4a,8a-dihydro-2*H*-chromen-6(5*H*)-one (**4k**)

The title compound was prepared according to the general procedure as described above (i) in an 88% (98 mg) yield; it was purified by column chromatography (20% EtOAc/hexane; R_*f*_ = 0.2) to afford **4k** as a white solid (mp:175–178 °C). ^**1**^**H NMR (400 MHz, CDCl**_**3**_**)**: δ 7.29 (d, *J* = 8.3 Hz, 2H), 7.21 (dt, *J* = 6.6, 5.2 Hz, 4H), 7.13 (d, *J* = 7.5 Hz, 2H), 7.07 (d, *J* = 8.0 Hz, 3H), 6.96 (d, *J* = 7.8 Hz, 2H), 6.45 (d, *J* = 10.2 Hz, 1H), 6.00 (dd, *J* = 10.2, 1.3 Hz, 1H), 4.86 (d, *J* = 17.6 Hz, 1H), 4.57 (dd, *J* = 17.6, 2.5 Hz, 1H), 3.26 (dd, *J* = 4.2, 1.2 Hz, 1H), 2.76 (dd, *J* = 3.9, 2.3 Hz, 1H), 2.32 (s, 3H), 2.23 (s, 3H), 1.38 (s, 3H). ^**13**^**C NMR (125 MHz, CDCl**_**3**_**)**: δ 210.8, 197.8, 193.3, 146.1, 145.4, 144.2, 138.5, 138.2, 138.0, 135.5, 134.5, 129.9, 129.3, 128.7, 127.9, 127.6, 127.4, 68.4, 61.2, 48.3, 47.3, 22.6, 21.6, 21.2. **IR (neat)**ν_max_: 3352, 2944, 2831, 1024, 750. **HRMS (ESI-TOF)**: *m*/*z* calcd for C_30_H_29_O_4_SSe [M + H]^+^, 565.0926; found, 565.0946.

#### (4a*R*,5*R*,8a*R*)-5-((4-Methoxyphenyl)selanyl)-8a-methyl-4-phenyl-3-(phenylsulfonyl)-4a,8a-dihydro-2*H*-chromen-6(5*H*)-one (**4l**)

The title compound was prepared according to the general procedure as described above (i) in an 85% (93 mg) yield; it was purified by column chromatography (20% EtOAc/hexane; R_*f*_ = 0.2) to afford **4l** as a white solid (mp: 180–185 °C). ^**1**^**H NMR (500 MHz, CDCl**_**3**_**)**: δ 7.51 (t, *J* = 7.4 Hz, 1H), 7.47 (d, *J* = 7.5 Hz, 2H), 7.36–7.28 (m, 6H), 7.22 (t, *J* = 7.1 Hz, 3H), 6.76 (d, *J* = 8.6 Hz, 2H), 6.53 (d, *J* = 10.2 Hz, 1H), 6.08 (d, *J* = 10.1 Hz, 1H), 4.97 (d, *J* = 17.7 Hz, 1H), 4.67 (dd, *J* = 17.7, 2.1 Hz, 1H), 3.76 (s, 3H), 3.26 (d, *J* = 3.4 Hz, 1H), 2.83 (s, 1H), 1.45 (s, 3H). ^**13**^**C NMR (100 MHz, CDCl**_**3**_**)**: δ 193.2, 160.1, 146.0, 145.9, 141.0, 138.1, 136.7, 135.3, 133.2, 128.7, 128.0, 127.6, 127.3, 120.2, 114.8, 68.4, 61.2, 55.2, 48.3, 48.1, 22.6. **IR (neat)**ν_max_: 3458, 2962, 1674, 1584, 1244, 1148, 692. **HRMS (ESI-TOF)**: *m*/*z* calcd for C_29_H_27_O_5_SSe [M + H]^+^, 567.0723; found, 567.0738.

#### (4a*R*,5*R*,8a*R*)-5-((3-Fluorophenyl)selanyl)-8a-methyl-4-phenyl-3-tosyl-4a,8a-dihydro-2*H*-chromen-6(5*H*)-one (**4m**)

The title compound was prepared according to the general procedure as described above (i) in a 90% (99 mg) yield; it was purified by column chromatography (20% EtOAc/hexane; R_*f*_ = 0.2) to afford **4m** as a white solid (mp: 222–228 °C). ^**1**^**H NMR (400 MHz, CDCl**_**3**_**)**: δ 7.35 (d, *J* = 8.3 Hz, 2H), 7.29 (d, *J* = 7.5 Hz, 1H), 7.21–7.14 (m, 7H), 7.12–7.09 (m, 1H), 6.98–6.93 (m, 2H), 6.55 (d, *J* = 10.2 Hz, 1H), 6.09 (dd, *J* = 10.2, 1.2 Hz, 1H), 4.93 (d, *J* = 17.7 Hz, 1H), 4.66 (dd, *J* = 17.7, 2.4 Hz, 1H), 3.41 (dd, *J* = 4.2, 1.1 Hz, 1H), 2.86 (dd, *J* = 3.9, 2.3 Hz, 1H), 2.40 (s, 3H), 1.47 (s, 3H). ^**13**^**C NMR (100 MHz, CDCl**_**3**_**)**: δ 193.6, 163.5, 161.0, 146.6, 145.2, 144.4, 129.4, 129.1, 129.1, 128.8, 128.0, 127.6, 127.5, 120.4, 120.1, 115.3, 115.1, 68.4, 61.3, 48.3, 47.4, 22.5, 21.6. **IR (neat)**ν_max_: 3462, 2968, 1670, 1582, 1240, 1142, 720. **HRMS (ESI-TOF)**: *m*/*z* calcd for C_29_H_26_FO_4_SSe [M + H]^+^, 569.0681; found, 569.0695.

#### (4a*R*,5*R*,8a*R*)-5-((2-Fluorophenyl)selanyl)-8a-methyl-4-phenyl-3-tosyl-4a,8a-dihydro-2*H*-chromen-6(5*H*)-one (**4n**)

The title compound was prepared according to the general procedure as described above (i) in an 80% (88 mg) yield; it was purified by column chromatography (20% EtOAc/hexane; R_*f*_ = 0.2) to afford **4n** as a white solid (mp: 178–183 °C). ^**1**^**H NMR (500 MHz, CDCl**_**3**_**)**: δ 7.43–7.40 (m, 1H), 7.37 (d, *J* = 8.2 Hz, 2H), 7.32–7.27 (m, 2H), 7.23 (t, *J* = 7.7 Hz, 3H), 7.14 (d, *J* = 8.1 Hz, 2H), 7.05–7.00 (m, 3H), 6.55 (d, *J* = 10.2 Hz, 1H), 6.06 (dd, *J* = 10.2, 1.0 Hz, 1H), 4.92 (d, *J* = 17.7 Hz, 1H), 4.67 (dd, *J* = 17.7, 2.4 Hz, 1H), 3.55 (dd, *J* = 4.0, 0.9 Hz, 1H), 2.86 (dd, *J* = 3.5, 2.5 Hz, 1H), 2.38 (s, 3H), 1.48 (s, 3H). ^**13**^**C NMR (100 MHz, CDCl**_**3**_**)**: δ. 193.0, 163.3, 160.9, 146.2, 145.2, 144.2, 138.2, 137.8, 136.4, 135.3, 130.7, 130.7, 129.4, 128.6, 127.9, 127.4, 127.3, 124.8, 124.8, 116.5, 116.2, 115.5, 115.3, 68.5, 61.3, 48.4, 45.6, 22.5, 21.5. **IR (neat) ν**_**max**_: 3022, 2976, 1674, 1443, 1146, 745. **HRMS (ESI-TOF)**: *m*/*z* calcd for C_29_H_26_FO_4_SSe [M + H]^+:^ 569.0681; found, 569.0695.

#### (4a*R*,5*R*,8a*R*)-5-((4-Methoxyphenyl)selanyl)-8a-methyl-4-phenyl-3-tosyl-4a,8a-dihydro-2*H*-chromen-6(5*H*)-one (**4o**)

The title compound was prepared according to the general procedure as described above (i) in a 95% (107 mg) yield; it was purified by column chromatography (20% EtOAc/hexane; R_*f*_ = 0.2) to afford **4o** as a white solid (mp: 205–209 °C). ^**1**^**H NMR (400 MHz, CDCl**_**3**_**)**: δ 7.37–7.29 (m, *J* = 15.1, 7.4 Hz, 5H), 7.26–7.23 (m, 3H), 7.14 (d, *J* = 8.0 Hz, 3H), 6.76 (d, *J* = 8.6 Hz, 2H), 6.52 (d, *J* = 10.2 Hz, 1H), 6.07 (d, *J* = 10.2 Hz, 1H), 4.94 (d, *J* = 17.6 Hz, 1H), 4.64 (dd, *J* = 17.6, 2.1 Hz, 1H), 3.76 (s, 3H), 3.27 (d, *J* = 3.5 Hz, 1H), 2.83 (s, 1H), 2.39 (s, 3H), 1.44 (s, 3H). ^**13**^**C NMR (100 MHz, CDCl**_**3**_**)**: δ 193.3, 160.1, 146.1, 145.4, 144.2, 138.2, 138.0, 136.7, 135.5, 129.3, 128.7, 127.9, 127.6, 127.4, 120.3, 114.7, 68.4, 61.2, 55.2, 48.3, 48.2, 22.6, 21.6. **IR (neat)**ν_max_: 3420, 2948, 1680, 1495, 1248, 1151, 763. **HRMS (ESI-TOF)**: *m*/*z* calcd for C_30_H_29_O_5_SSe [M + H]^+^, 581.0885; found, 581.0895.

#### (4a*R*,5*R*,8a*R*)-8a-Methyl-4-phenyl-5-(thiophen-2-ylselanyl)-3-tosyl-4a,8a-dihydro-2*H*-chromen-6(5*H*)-one (**4p**)

The title compound was prepared according to the general procedure as described above (i) in an 85% (92 mg) yield; it was purified by column chromatography (20% EtOAc/hexane; R_*f*_ = 0.2) to afford **4p** as a white solid (mp: 180–185 °C). ^**1**^**H NMR (400 MHz, CDCl**_**3**_**)**: δ 7.37 (d, *J* = 7.5 Hz, 3H), 7.33–7.26 (m, 4H), 7.15 (d, *J* = 8.0 Hz, 3H), 7.08 (d, *J* = 2.8 Hz, 1H), 6.94 (dd, *J* = 5.1, 3.6 Hz, 1H), 6.53 (d, *J* = 10.2 Hz, 1H), 6.09 (d, *J* = 10.2 Hz, 1H), 4.92 (d, *J* = 17.6 Hz, 1H), 4.65 (dd, *J* = 17.7, 2.2 Hz, 1H), 3.29 (d, *J* = 3.2 Hz, 1H), 2.80 (s, 1H), 2.39 (s, 3H), 1.46 (s, 3H). ^**13**^**C NMR (101 MHz, CDCl**_**3**_**)**: δ 192.9, 146.1, 145.3, 144.3, 142.4, 138.2, 137.9, 137.12, 135.4, 132.1, 129.4, 128.8, 128.1, 128.0, 127.5, 127.4, 124.1, 68.5, 61.3, 50.4, 48.4, 40.2, 22.3, 21.6. **IR (neat)**ν_max_: 3428, 2938, 1677, 1312, 1146, 703. **HRMS (ESI-TOF)**: *m*/*z* calcd for C_27_H_25_O_4_S_2_Se [M + H]^+^, 557.0339; found, 557.0354.

#### (4a*R*,5*R*,8a*R*)-8a-Methyl-4-phenyl-3-(phenylsulfonyl)-5-(*p*-tolylselanyl)-4a,8a-dihydro-2*H*-chromen-6(5*H*)-one (**4q**)

The title compound was prepared according to the general procedure as described above (i) in an 80% (85 mg) yield; it was purified by column chromatography (20% EtOAc/hexane; R_*f*_ = 0.2) to afford **4q** as a white solid (mp:140–145 °C). ^**1**^**H NMR (400 MHz, CDCl**_**3**_**)**: δ 7.46–7.39 (m, 3H), 7.27 (dd, *J* = 14.6, 6.5 Hz, 3H), 7.24–7.19 (m, 3H), 7.13 (t, *J* = 7.6 Hz, 2H), 6.97 (d, *J* = 7.9 Hz, 3H), 6.46 (d, *J* = 10.2 Hz, 1H), 6.01 (dd, *J* = 10.2, 1.1 Hz, 1H), 4.89 (d, *J* = 17.7 Hz, 1H), 4.61 (dd, *J* = 17.7, 2.4 Hz, 1H), 3.26 (dd, *J* = 4.1, 1.0 Hz, 1H), 2.78–2.76 (m, 1H), 2.23 (s, 3H), 1.39 (s, 3H). ^**13**^**C NMR (125 MHz, CDCl**_**3**_**)**: δ 193.3, 146.1, 145.9, 141.0, 138.6, 138.1, 135.3, 134.6, 133.2, 130.0, 128.8, 128.7, 128.0, 127.6, 127.3, 126.3, 68.4, 61.3, 48.3, 47.6, 22.6, 21.2. **IR (neat)**ν_max_: 3450, 2980, 1678, 1313, 1151, 1082, 728. **HRMS (ESI-TOF)**: *m*/*z* calcd for C_29_H_27_O_4_SSe [M + H]^+^, 551.07726; found, 551.07898.

#### (4a*R*,5*R*,8a*R*)-3-((4-Chlorophenyl)sulfonyl)-8a-methyl-4-phenyl-5-(phenylselanyl)-4a,8a-dihydro-2*H*-chromen-6(5*H*)-one (**4r**)

The title compound was prepared according to the general procedure as described above (i) in an 86% (95 mg) yield; it was purified by column chromatography (20% EtOAc/hexane; R_*f*_ = 0.2) to afford **4r** as a white solid (mp:155–160 °C). ^**1**^**H NMR (500 MHz, CDCl**_**3**_**)**: δ 7.41–7.39 (m, 2H), 7.37–7.34 (m, 2H), 7.32–7.29 (m, 3H), 7.28–7.21 (m, 4H), 7.20 (t, *J* = 7.9 Hz, 2H), 6.98 (s, 1H), 6.54 (d, *J* = 10.2 Hz, 1H), 6.10 (dd, *J* = 10.2, 1.2 Hz, 1H), 4.96 (d, *J* = 17.7 Hz, 1H), 4.68 (dd, *J* = 17.7, 2.5 Hz, 1H), 3.39 (dd, *J* = 4.2, 1.2 Hz, 1H), 2.86 (dd, *J* = 3.8, 2.4 Hz, 1H), 1.47 (s, 3H). ^**13**^**C NMR (100 MHz, CDCl**_**3**_**)**: δ 193.4, 146.2, 139.9, 139.4, 138.1, 135.1, 133.9, 130.1, 129.1, 129.0, 128.8, 128.3, 128.1, 127.7, 68.4, 61.1, 48.4, 47.3, 22.6. **IR (neat)**ν_max_: 3020, 2852, 1673, 1313, 1148, 752. **HRMS (ESI-TOF)**: *m*/*z* calcd for C_28_H_24_O_4_ClSSe [M + H]^+^, 571.0222; found, 571.0246.

#### (4a*R*,5*R*,8a*R*)-3-(Cyclopropylsulfonyl)-8a-methyl-4-phenyl-5-(phenylselanyl)-4a,8a-dihydro-2*H*-chromen-6(5*H*)-one (**4s**)

The title compound was prepared according to the general procedure as described above (i) in an 82% (79 mg) yield; it was purified by column chromatography (20% EtOAc/hexane; R_*f*_ = 0.2) to afford **4s** as a white solid (mp: 144–148 °C). ^**1**^**H NMR (500 MHz, CDCl**_**3**_**)**: δ 7.50–7.47 (m, 2H), 7.38 (s, 4H), 7.31–7.24 (m, 4H), 6.58 (d, *J* = 10.2 Hz, 1H), 6.12 (dd, *J* = 10.2, 1.3 Hz, 1H), 4.83 (d, *J* = 17.6 Hz, 1H), 4.58 (dd, *J* = 17.6, 2.5 Hz, 1H), 3.51 (dd, *J* = 4.3, 1.2 Hz, 1H), 3.03 (dd, *J* = 3.9, 2.4 Hz, 1H), 2.04 (dq, *J* = 8.0, 4.9 Hz, 1H), 1.57 (s, 3H), 1.21 (ddt, *J* = 10.0, 6.8, 4.9 Hz, 1H), 1.12–1.06 (m, 1H), 0.97–0.92 (m, 1H), 0.89–0.84 (m, 1H). ^**13**^**C NMR (100 MHz, CDCl**_**3**_**)**: δ 193.5, 146.3, 144.3, 137.8, 136.1, 134.0, 130.1, 129.2, 129.2, 128.4, 128.3, 127.6, 68.4, 61.3, 48.2, 47.1, 32.5, 22.6, 5.8, 5.3. **IR (neat)**ν_max_: 3051, 2976, 1672, 1300, 1144, 701. **HRMS (ESI-TOF)**: *m*/*z* calcd for C_25_H_25_O_4_SSe [M + H]^+^, 501.0621; found, 501.0633.

#### (4a*R*,5*R*,8a*R*)-4-(2-Fluorophenyl)-8a-methyl-5-(phenylselanyl)-3-(phenylsulfonyl)-4a,8a-dihydro-2*H*-chromen-6(5*H*)-one (**4t**)

The title compound was prepared according to the general procedure as described above (i) in a 75% (85 mg) yield; it was purified by column chromatography (20% EtOAc/hexane; R_*f*_ = 0.2) to afford **4t** as a white solid (mp: 154–158 ^ο^C) ^**1**^**H NMR (400 MHz, CDCl**_**3**_**)**: δ 7.50–7.42 (m, 4H), 7.38–7.29 (m, 3H), 7.27–7.20 (m, 4H), 7.05–6.96 (m, 3H), 6.79–6.75 (m, 1H), 6.49 (d, *J* = 10.2 Hz, 1H), 6.00 (dd, *J* = 10.2, 1.3 Hz, 1H), 4.89 (d, *J* = 17.9 Hz, 1H), 4.65 (dd, *J* = 17.9, 2.4 Hz, 1H), 3.52 (dd, *J* = 4.2, 1.1 Hz, 1H), 2.88 (dd, *J* = 3.9, 2.3 Hz, 1H), 1.42 (s, 3H). ^**13**^**C NMR (100 MHz, CDCl**_**3**_**)**: δ 192.9, 146.3, 140.8, 140.3, 140.0, 136.8, 133.4, 132.1, 131.0, 131.0, 128.9, 127.2, 124.9, 124.8, 123.6, 123.6, 115.6, 115.4, 115.3, 68.7, 61.5, 46.3, 45.8, 22.2**.IR (neat)**ν_max_: 3022, 2976, 1674, 1443, 1146, 745. **HRMS (ESI-TOF)**: *m*/*z* calcd for C_28_H_24_FO_4_SSe [M + H]^+^, 554.0466; found, 554.0476.

#### (3a*S*,4*R*,7a*S*,*Z*)-7a-Methyl-4-(phenylselanyl)-3-(tosylmethylene)-2,3,3a,7a-tetrahydrobenzofuran-5(4*H*)-one (**6a**)

The title compound was prepared according to the general procedure as described above (ii) in an 88% (140 mg) yield; it was purified by column chromatography (20% EtOAc/hexane; R_*f*_ = 0.2) to afford **6a** as a white solid(155–160 °C). ^**1**^**H NMR (400 MHz, CDCl**_**3**_**)**: δ 7.65 (d, *J* = 8.0 Hz, 2H), 7.57 (d, *J* = 7.0 Hz, 2H), 7.39–7.29 (m, 5H), 6.44 (d, *J* = 10.2 Hz, 1H), 6.07 (d, *J* = 2.0 Hz, 1H), 5.98 (d, *J* = 10.4 Hz, 1H), 5.12 (dd, *J* = 17.4, 1.3 Hz, 1H), 4.56 (d, *J* = 17.4 Hz, 1H), 3.85 (s, 1H), 3.29 (s, 1H), 2.41 (s, 3H), 1.72 (s, 3H). ^**13**^**C NMR (100 MHz, CDCl**_**3**_**)**: δ 191.2, 158.5, 148.5, 144.9, 137.6, 135.3, 130.0, 129.5, 129.3, 128.4, 127.2, 123.7, 78.7, 68.2, 53.8, 45.4, 25.0, 21.6. **IR (neat)**ν_max_: 3405, 2940, 1683, 1444, 1303, 1145, 742. **HRMS (ESI-TOF)**: *m*/*z* calcd for C_23_H_23_O_4_SSe [M + H]^+^, 475.0466, Found 475.0476.

#### (3a*S*,4*R*,7a*S*,*Z*)-7a-Methyl-4-(*p*-tolylselanyl)-3-(tosylmethylene)-2,3,3a,7a-tetrahydrobenzofuran-5(4*H*)-one (**6b**)

The title compound was prepared according to the general procedure as described above (iii) in an 87% (143 mg) yield; it was purified by column chromatography (20% EtOAc/hexane; R_*f*_ = 0.2) to afford **6b** as a white solid (mp: 175–180 °C). ^**1**^**H NMR (500 MHz, CDCl**_**3**_**)**: δ 7.64 (d, *J* = 8.2 Hz, 2H), 7.44 (d, *J* = 8.0 Hz, 2H), 7.29 (d, *J* = 8.0 Hz, 2H), 7.13 (d, *J* = 7.8 Hz, 2H), 6.43 (dd, *J* = 10.4, 1.7 Hz, 1H), 6.04 (d, *J* = 2.4 Hz, 1H), 5.97 (dd, *J* = 10.4, 0.9 Hz, 1H), 5.12 (dd, *J* = 17.4, 2.2 Hz, 1H), 4.58–4.53 (m, 1H), 3.80 (s, 1H), 3.28 (s, 1H), 2.41 (s, 3H), 2.35 (s, 3H), 1.72 (s, 3H). ^**13**^**C NMR (100 MHz, CDCl**_**3**_**)**: δ 191.1, 158.6, 148.4, 144.8, 139.7, 137.6, 135.7, 130.4, 130.0, 128.4, 127.2, 124.4, 123.6, 78.7, 68.2, 53.7, 45.6, 25.0, 21.6, 21.3. **IR (neat)**ν_max_:3482, 2926, 1675, 1306, 1148, 809. **HRMS (ESI-TOF)**: *m*/*z* calcd for C_24_H_25_O_4_SSe [M + H]^+^, 489.0621; found, 489. 0633

#### (3a*S*,4*R*,7a*S,Z*)-4-((4-Methoxyphenyl)selanyl)-7a-methyl-3-(tosylmethylene)-2,3,3a,7a-tetrahydrobenzofuran-5(4*H*)-one (**6c**)

The title compound was prepared according to the general procedure as described above (ii) in a 92% (156 mg) yield; it was purified by column chromatography (20% EtOAc/hexane; R_*f*_ = 0.2) to afford **6c** as a white solid (mp:153–156 °C). ^**1**^**H NMR (500 MHz, CDCl**_**3**_**)**: δ 7.64 (d, *J* = 7.9 Hz, 2H), 7.47 (d, *J* = 8.3 Hz, 2H), 7.29 (d, *J* = 7.8 Hz, 2H), 6.84 (d, *J* = 8.3 Hz, 2H), 6.43 (d, *J* = 10.3 Hz, 1H), 6.03 (s, 1H), 5.97 (d, *J* = 10.4 Hz, 1H), 5.12 (d, *J* = 17.4 Hz, 1H), 4.56 (d, *J* = 17.4 Hz, 1H), 3.80 (s, 3H), 3.74 (s, 1H), 3.27 (s, 1H), 2.41 (s, 3H), 1.72 (s, 3H). ^**13**^**C NMR (100 MHz, CDCl**_**3**_**)**: δ 191.1, 160.8, 158.6, 148.4, 144.9, 137.8, 137.6, 130.0, 128.4, 127.2, 123.5, 118.3, 115.2, 78.7, 68.2, 55.3, 53.6, 46.0, 25.0, 21.6. **IR (neat)**ν_max_: 3498, 2979, 1676, 1491, 1246, 1146, 819. **HRMS (ESI-TOF)**: *m*/*z* calcd for C_24_H_25_O_5_SSe [M + H]^+^, 505.0569; found, 505.0582.

#### (3a*S*,4*R*,7a*S,Z*)-4-((3-Fluorophenyl)selanyl)-7a-methyl-3-(tosylmethylene)-2,3,3a,7a-tetrahydrobenzofuran-5(4*H*)-one (**6d**)

The title compound was prepared according to the general procedure as described above (ii) in a 78% (118 mg) yield; it was purified by column chromatography (20% EtOAc/hexane; R_*f*_ = 0.2) to afford **6d** as a white solid (mp:133–146 °C). ^**1**^**H NMR (500 MHz, CDCl**_**3**_**)**: δ 7.76 (d, *J* = 8.2 Hz, 0.7H), 7.66 (d, *J* = 8.3 Hz, 2H), 7.35–7.26 (m, 6H), 7.22–6.98 (m, 2.2.H), 6.92 (d, *J* = 1.9 Hz, 0.35H), 6.54 (d, *J* = 10.2 Hz, 0.35H), 6.45 (dd, *J* = 10.4, 1.7 Hz, 1H), 6.09 (dd, *J* = 5.0, 2.4 Hz, 1H), 6.04 (d, *J* = 10.2 Hz, 0.35H), 5.99 (dd, *J* = 10.4, 1.1 Hz, 1H), 5.12 (dd, *J* = 17.4, 2.2 Hz, 1H), 5.00 (dd, *J* = 17.4, 2.3 Hz, 0.35H), 4.93–4.89 (m, 0.35H), 4.57 (dt, *J* = 17.4, 2.4 Hz, 1H), 4.03 (d, *J* = 4.9 Hz, 0.35H), 3.87–3.87 (m, 1H), 3.30–3.30 (m, 1H), 3.14–3.17 (m, 0.35H), 2.43 (s, 1H), 2.42 (s, 3H), 1.70 (s, 3H), 1.39 (s, 1H). ^**13**^**C NMR (100 MHz, CDCl**_**3**_**)**: δ 192.3, 191.0, 163.6, 161.1, 158.2, 156.9, 148.7, 148.0, 144.9, 137.9, 137.5, 130.8, 130.7, 130.6, 130.6, 130.4, 130.3, 130.0, 129.5, 129.4, 128.5, 128.3, 127.2, 127.1, 123.8, 123.7, 122.0, 121.8, 121.5, 121.3, 116.5, 116.3, 116.0, 115.8, 79.5, 78.6, 69.1, 68.2, 53.8, 52.3, 49.7, 45.3, 25.0, 22.7, 21.6 **IR (neat)**ν_max_: 3049, 2929, 1672, 1475, 1280, 1148, 811. **HRMS (ESI-TOF)**: *m*/*z* calcd for C_23_H_22_O_4_FSSe [M + H]^+^, 493.0367; found, 493.0382.

#### (3a*S*,4*R*,7a*S,Z*)-7a-Methyl-3-((phenylsulfonyl)methylene)-4-(*p*-tolylselanyl)-2,3,3a,7a-tetrahydrobenzofuran-5(4*H*)-one (**6e**)

The title compound was prepared according to the general procedure as described above (ii) in a 91% (145 mg) yield; it was purified by column chromatography (20% EtOAc/hexane; R_*f*_ = 0.2) to afford **6e** as a white solid (mp: 158–163 °C). ^**1**^**H NMR (400 MHz, CDCl**_**3**_**)**: δ 7.78–7.76(m, 2H), 7.61–7.53 (m, 1H), 7.51–7.44 (m, 2H), 7.26–7.72 (m, 2H), 7.13 (d, *J* = 7.8 Hz, 2H), 6.43 (dd, *J* = 10.4, 1.8 Hz, 1H), 6.43 (dd, *J* = 10.4, 1.8 Hz, 1H), 6.07 (q, *J* = 2.5 Hz, 1H), 5.97 (dd, *J* = 10.4, 1.2 Hz, 1H), 5.13 (dd, *J* = 17.5, 2.3 Hz, 1H), 4.56 (dt, *J* = 17.5, 2.5 Hz, 1H), 3.80 (dd, *J* = 2.3, 1.3 Hz, 1H), 3.29–3.28 (m, 1H), 2.35 (s, 3H), 1.73 (s, 3H). ^**13**^**C NMR (100 MHz, CDCl**_**3**_**)**: δ 191.10, 159.29, 148.38, 140.51, 139.71, 135.68, 133.77, 130.38, 129.35, 128.42, 127.13, 124.39, 123.30, 78.72, 77.00, 68.17, 53.75, 45.60, 24.96, 21.27. **IR (neat) ν**_**max**_: 3492, 2974, 1677, 1305, 1147, 1082, 802. **HRMS (ESI-TOF)**: *m*/*z* calcd for C_23_H_23_O_4_SSe [M + H]^+^, 475.0466; found, 475.0476.

#### (3a*S*,4*R*,7a*S*,*Z*)-4-((4-Methoxyphenyl)selanyl)-7a-methyl-3-((phenylsulfonyl)methylene)-2,3,3a,7a-tetrahydrobenzofuran-5(4*H*)-one (**6f**)

The title compound was prepared according to the general procedure as described above (ii) in an 86% (142 mg) yield; it was purified by column chromatography (20% EtOAc/hexane; R_*f*_ = 0.2) to afford **6f** as a white solid (mp: 160–165 °C). ^**1**^**H NMR (400 MHz, CDCl**_**3**_**)**: δ 7.76 (dd, *J* = 5.2, 3.3 Hz, 2H), 7.62–7.58 (m, 1H), 7.52–7.45 (m, 4H), 6.86–6.82 (m, 2H), 6.43 (dd, *J* = 10.4, 1.8 Hz, 1H), 6.06 (q, *J* = 2.5 Hz, 1H), 5.97 (dd, *J* = 10.4, 1.2 Hz, 1H), 5.12 (dd, *J* = 17.5, 2.3 Hz, 1H), 4.56 (dt, *J* = 17.5, 2.5 Hz, 1H), 3.79 (s, 3H), 3.75 (dd, *J* = 2.2, 1.3 Hz, 1H), 3.33–3.23 (m, 1H), 1.72 (s, 3H). ^**13**^**C NMR (100 MHz, CDCl**_**3**_**)**: δ 191.1, 160.8, 159.3, 148.3, 140.5, 137.7, 133.8, 129.3, 128.4, 127.1, 123.3, 118.2, 115.2, 78.7, 68.2, 55.3, 53.7, 45.9, 25.0. **IR (neat)**ν_max_:3499, 2478, 1678, 1298, 1249, 1150, 755 **HRMS (ESI-TOF)**: *m*/*z* calcd for C_23_H_23_O_5_SSe [M + H]^+^, 491.0415; found, 491.0421.

#### (3a*S*,4*R*,7a*S*,*Z*)-3-(((4-Chlorophenyl)sulfonyl)methylene)-7a-methyl-4-(phenylselanyl)-2,3,3a,7a-tetrahydrobenzofuran-5(4*H*)-one (**6g**)

The title compound was prepared according to the general procedure as described above (ii) in an 80% (133 mg) yield; it was purified by column chromatography (20% EtOAc/hexane; R_*f*_ = 0.2) to afford **6g** as a white solid (mp: 194–198 °C). ^**1**^**H NMR (500 MHz, CDCl**_**3**_**)**: δ 7.70 (d, *J* = 8.5 Hz, 2H), 7.57 (d, *J* = 7.0 Hz, 2H), 7.48 (d, *J* = 8.4 Hz, 2H), 7.35 (dt, *J* = 24.7, 7.2 Hz, 3H), 6.44 (dd, *J* = 10.4, 1.4 Hz, 1H), 6.07 (d, *J* = 0.9 Hz, 1H), 5.99 (d, *J* = 10.4 Hz, 1H), 5.11 (dd, *J* = 17.6, 1.8 Hz, 1H), 4.55 (dt, *J* = 17.6, 2.4 Hz, 1H), 3.86 (d, *J* = 0.7 Hz, 1H), 3.31 (s, 1H), 1.73 (s, 3H). ^**13**^**C NMR (100 MHz, CDCl**_**3**_**)**: δ 191.18, 159.90, 148.42, 140.56, 138.93, 135.32, 129.70, 129.55, 129.32, 128.61, 128.45, 128.03, 122.99, 78.74, 77.00, 68.13, 53.92, 45.34, 24.96. **IR (neat)**ν_max_;3049, 2929, 1672, 1313, 1148, 1086, 752. **HRMS (ESI-TOF)**: *m*/*z* calcd for C_22_H_20_O_4_ClSSe [M + H]^+^, 494.9913; found, 494.9930.

#### (3a*S*,4*R*,7a*S*,*Z*)-3-((Cyclopropylsulfonyl)methylene)-7a-methyl-4-(phenylselanyl)-2,3,3a,7a-tetrahydrobenzofuran-5(4*H*)-one (**6h**)

The title compound was prepared according to the general procedure as described above (ii) in a 79% (112 mg) yield; it was purified by column chromatography (20% EtOAc/hexane; R_*f*_ = 0.2) to afford **6h** as a white solid (mp: 172–177 °C). ^**1**^**H NMR (500 MHz, CDCl**_**3**_**)**: δ 7.64–7.62 (m, 2H), 7.42–7.36 (m, 3H), 6.46 (dd, *J* = 10.4, 1.8 Hz, 1H), 6.09 (q, *J* = 2.6 Hz, 1H), 6.04 (dd, *J* = 10.4, 1.3 Hz, 1H), 5.02 (dd, *J* = 17.4, 2.3 Hz, 1H), 4.48 (dt, *J* = 17.4, 2.5 Hz, 1H), 3.95 (dd, *J* = 2.3, 1.3 Hz, 1H), 3.35 (dt, *J* = 4.2, 2.0 Hz, 1H), 2.30 (tt, *J* = 8.0, 4.8 Hz, 1H), 1.75 (s, 3H), 1.22–1.10 (m, 2H), 1.04–0.93 (m, 2H). ^**13**^**C NMR (100 MHz, CDCl**_**3**_**)**: δ 191.4, 159.3, 148.6, 135.3, 129.6, 129.3, 128.4, 128.2, 121.6, 78.6, 68.1, 53.8, 45.6, 31.6, 25.0, 5.2, 4.9. **IR (neat)**ν_max_: 3047, 2930, 1671, 1285, 1131, 1048, 746. **HRMS (ESI-TOF)**: *m*/*z* calcd for C_19_H_21_O_4_SSe [M + H]^+^, 425.0305; found, 425.0320.

#### (3a*S*,4*R*,7a*S*,*Z*)-7a-Methyl-4-(phenylselanyl)-3-((phenylsulfonyl)methylene)-2,3,3a,7a-tetrahydrobenzofuran-5(4*H*)-one (**6i**)

The title compound was prepared according to the general procedure as described above (ii) in a 93% (144 mg) yield; it was purified by column chromatography (20% EtOAc/hexane; R_*f*_ = 0.2) to afford **6i** as a white solid (mp: 162–168 °C). ^**1**^**H NMR (400 MHz, CDCl**_**3**_**)**: δ 7.79–7.71 (m, 2H), 7.61–7.51 (m, 6H), 7.36–7.3 (m, 2H), 6.44 (dd, *J* = 10.4, 1.8 Hz, 1H), 6.09 (q, *J* = 2.5 Hz, 1H), 5.98 (dd, *J* = 10.4, 1.2 Hz, 1H), 5.13 (dd, *J* = 17.5, 2.3 Hz, 1H), 4.57 (dt, *J* = 17.5, 2.5 Hz, 1H), 3.86 (dd, *J* = 2.3, 1.3 Hz, 1H), 3.32–3.31 (m, 1H), 1.73 (s, 3H). ^**13**^**C NMR (100 MHz, CDCl**_**3**_**)**: δ 191.2, 159.2, 148.5, 135.3, 133.8, 129.6, 129.4, 129.3, 128.5, 127.2, 123.4, 78.7, 68.2, 53.9, 45.4, 25.0. **IR (neat)**ν_max_: 3492, 2974, 1677, 1305, 1147, 1082, 802. **HRMS (ESI-TOF)**: *m*/*z* calcd for C_22_H_21_O_4_SSe [M + H]^+^, 461.0302; found, 461.0320.

#### (3aS,4R,7aS,*Z*)-6,7a-Dimethyl-4-(phenylselanyl)-3-(tosylmethylene)-2,3,3a,7a-tetrahydrobenzofuran-5(4*H*)-one (**6j**)

The title compound was prepared according to the general procedure as described above (ii) in an 85% (127 mg) yield; it was purified by column chromatography (20% EtOAc/hexane; R_*f*_ = 0.2) to afford **6j** as a white solid (mp:143–146 °C). ^**1**^**H NMR (400 MHz, CDCl**_**3**_**)**: δ 7.57 (d, *J* = 8.0 Hz, 2H), 7.48 (d, *J* = 6.9 Hz, 2H), 7.31–7.18 (m, 5H), 6.12 (s, = 1H), 5.99 (d, *J* = 1.9 Hz, 1H), 5.00 (d, *J* = 17.4 Hz, 1H), 4.44 (d, *J* = 17.4 Hz, 1H), 3.80 (d, *J* = 1.9 Hz, 1H), 3.20 (s, 1H), 2.34 (s, 3H), 1.69 (s, 3H), 1.62 (s, 3H). ^**13**^**C NMR (100 MHz, CDCl**_**3**_**)**: δ 191.8, 159.2, 144.8, 143.6, 137.7, 135.2, 129.9, 129.5, 129.1, 127.1, 123.5, 79.1, 68.0, 54.1, 45.8, 25.4, 21.6, 16.3. **IR (neat)**ν_max_: 3478, 2977, 1672, 1302, 1145, 1047, 741. **HRMS (ESI-TOF)**: *m*/*z* calcd for C_24_H_25_O_4_SSe [M + H]^+^, 489.0621; found, 457.0633.

#### (3a*S*,4*R*,7a*S*,*Z*)-4-((2-Fluorophenyl)selanyl)-7a-methyl-3-((phenylsulfonyl)methylene)-2,3,3a,7a-tetrahydrobenzofuran-5(4*H*)-one (**6k**)

The title compound was prepared according to the general procedure as described above (ii) in a 78% (125 mg) yield; it was purified by column chromatography (20% EtOAc/hexane; R_*f*_ = 0.2) to afford **6k** as a white solid (mp:133–136 ^ο^C). ^**1**^**H NMR (400 MHz, CDCl**_**3**_**)**δ 7.73–7.71 (m, 2H), 7.55–7.53 (m, 1H), 7.50–7.43 (m, 3H), 7.32 (tdd, *J* = 7.2, 5.3, 1.7 Hz, 1H), 7.08–7.02 (m, 2H), 6.39 (dd, *J* = 10.4, 1.8 Hz, 1H), 6.05 (q, *J* = 2.5 Hz, 1H), 5.90 (dd, *J* = 10.4, 1.3 Hz, 1H), 5.06 (dd, *J* = 17.5, 2.3 Hz, 1H), 4.50 (dt, *J* = 17.4, 2.5 Hz, 1H), 3.93 (dd, *J* = 2.2, 1.3 Hz, 1H), 3.24–3.22 (m, 1H), 1.67 (s, 3H). ^**13**^**C NMR (100 MHz, CDCl**_**3**_**)**: δ 190.62, 159.12, 148.57, 140.48, 137.26, 133.81, 131.93, 131.85, 129.38, 128.29, 127.13, 125.15, 123.48, 116.08, 115.85, 114.64, 114.42, 78.59, 68.21, 53.45, 43.70, 24.92. **IR (neat)**ν_max_: 3049, 2929, 1672, 1475, 1280, 1148, 811. **HRMS (ESI-TOF)**:*m*/*z* calcd for C_22_H_20_FO_4_SSe [M + H]^+^, 478.0153; found, 478.0158.

#### 8a-Methyl-4-phenyl-3-*tosyl*-2*H*-chromen-6(8a*H*)-one (**7**)

The title compound was prepared according to the general procedure as described above in a 75% (25 mg) yield; it was purified by column chromatography (30% EtOAc/hexane; R_*f*_ = 0.2) to afford 7 as a colorless sticky solid. ^**1**^**H NMR (500 MHz, CDCl3)**: δ 7.24 (ddd, *J* = 7.6, 2.5, 1.2 Hz, 1H), 7.13 (t, *J* = 7.9 Hz, 4H), 7.03–7.01 (m, 2H), 6.99 (s, 1H), 6.88 (d, *J* = 7.2 Hz, 2H), 6.48 (s, 1H), 6.26 (d, *J* = 10.0 Hz, 1H), 4.93 (d, *J* = 18.9 Hz, 1H), 4.79 (d, *J* = 18.9 Hz, 1H), 2.31 (s, 3H), 1.50 (s, 3H). ^**13**^**C NMR (100 MHz, CDCl3)**: δ 181.2, 153.8, 144.5, 144.2, 142.3, 138.1, 134.7, 133.8, 129.4, 128.0, 127.4, 123.9, 70.6, 62.0, 23.8, 21.6. **IR (neat)**ν_max_: 1655,1485,1070,958,751. **HRMS (ESI-TOF)**: *m*/*z* calcd for C_23_ H_19_ O _4_ S = 391.10112[M + H]^+^, 391.0998; found, 391.0998.

#### 7a-Methyl-3-((phenylsulfonyl)methylene)-2,3-dihydrobenzofuran-5(7a*H*)-one (**8**)

The title compound was prepared according to the general procedure as described above in an 80% (26 mg) yield; it was purified by column chromatography (EtOAc/hexane) to afford 8 as a colorless sticky solid. **1H NMR (400 MHz, CDCl3)**: δ 7.95–7.92 (m, 2H), 7.72–7.68 (m, 1H), 7.62–7.59 (m, 2H), 7.18 (d, *J* = 9.9 Hz, 1H), 6.72 (t, *J* = 2.7 Hz, 1H), 6.27 (d, *J* = 1.4 Hz, 1H), 6.19–6.16 (m, 1H), 5.26 (dd, *J* = 17.4, 2.6 Hz, 1H), 5.11 (dd, *J* = 17.4, 2.7 Hz, 1H), 1.40 (s, 3H). **13C NMR (100 MHz, CDCl3)**: δ 185., 159.4, 148.1, 147.9, 140.1, 134.3, 129.7, 128.0, 127.6, 123.6, 120.1, 78.7, 68.7, 25.6. **IR (neat)**ν_max_: 1669,1303,1076,672 **HRMS (ESI-TOF)**:*m*/*z* calcd for C_16_H_15_O_4_S [M – H]^+^, 303.0696; found, 303.06856.

## Supporting Information Available

Copies of ^1^H and ^13^C NMR spectra for all new compounds (PDF)
