# Supplementary material for: Regioselective Radical Cascade Cyclizations of Alkyne-Tethered Cyclohexadienones with Chalcogenides under Visible-Light Catalysis
Source: ACS Omega. 2023 Sep 20;8(39):35809–21. doi: 10.1021/acsomega.3c03362 (PMC10552108; doi:10.1021/acsomega.3c03362)

Electronic Supplementary Information

Regioselective Radical Cascade Cyclizations of Alkyne Tethered Cyclohexadienones with Chalcogenides under Visible Light Catalysis

Vadla Shiva Prasad,[a,c] Vadithya Ranga Rao,[a,c] Maram Gangadhar,[a,c] Sunil Kumar Nechipadappu,[b,c] Praveen Reddy Adiyala *[a,c]

[a]Department of Organic Synthesis and Process Chemistry, CSIR-Indian Institute of Chemical Technology (CSIR-IICT), Hyderabad-500007, India.

[b]Department of Analytical Chemistry, CSIR-Indian Institute of Chemical Technology (CSIR-IICT), Hyderabad 500007, India.

[c]Academy of Scientific and Innovative Research (AcSIR), Ghaziabad 201002, India.

Phone: +914027191815; E-mail: praveenreddy@iict.res.in

Table of contents:

1) General Information……………………………………………………….…S2

2) Experimental Procedures……………………………………………………. S2-S7

3) X-ray Crystallography…………………………………………….………S8-S9

4) Reference…………………………………………………………….………S10

5) NMR copies of all synthesized compounds…………….………….………... S11-S43

1. General information:

Unless otherwise noted, all the commercial materials were used without further purification. All reactions were performed under an inert atmosphere and in oven-dried glassware with magnetic stirring. All solvents were dried before use following the standard procedures. Reactions were monitored by TLC on pre-coated silica gel 60 F-254. TLC plates were visualized with UV light (254 nm), iodine treatment, or using *p*-anisaldehyde stain. Column chromatography was carried out using silica gel (60-120 mesh & 100-200 mesh) packed in glass columns. NMR spectra were recorded at 300, 400, 500 MHz (H) and at 75, 100, 125 MHz (C), respectively. Chemical shifts (δ) are reported in ppm, using the residual solvent peak in $CDCl_3$ (H: δ = 7.26 and C: δ = 77.00 ppm) as internal standard, and coupling constants (J) are given in Hz. HRMS were recorded using ESI-TOF techniques.

2. Experimental Procedures:

a) General procedure for alkyl tethered cyclohexadienones.

All cyclohexadienones used were prepared according to following procedure. [1]

STEP-1

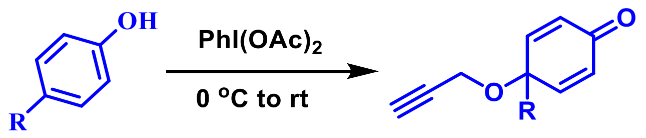

To a stirred solution of 4-substituted phenol (1.0 mmol, 1.0 equiv.) in 1 mL of propargyl alcohol was added phenyl iodine(III)diacetate (1.5 mmol, 1.5 equiv.) in several portions at 0 °C. The resulting reaction mixture was stirred at room temperature for overnight. Then the reaction mixture was diluted with water (10 mL) and extracted with ethyl acetate (3 x 15 mL). The combined organic solvent was washed with brine (15 mL), dried (Na_2SO_4), filtered and concentrated under reduced pressure.The crude reaction mixture was purified by column chromatography (20% EtOAc in hexanes) to give O-tethered alkyne.

STEP-2

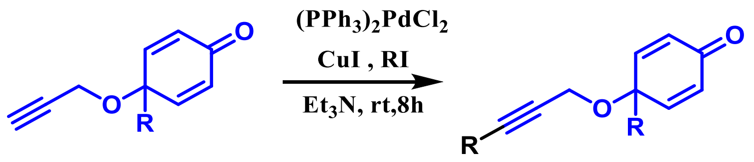

1

To the above *O*-tethered alkyne (10.0 mmol, 1.0 equiv.) in degassed Et_3N (1 M, 10 mL), was added $Pd(PPh_3)_2Cl_2$ (3 mol%), CuI (1.5 mol%) and aryl iodide (12 mmol, 1.2 equiv.). The mixture was stirred at room temperature for 8 h. The reaction was cooled to room temperature, water (50 mL) was added and the mixture was extracted with EtOAc (2 x 40 mL). The combined organic layer was dried over anhydrous Na_2SO_4, filtered, and concentrated in vacuo. The mixture was purified by column chromatography (EtOAc/hexane) to give aryl substituted alkynes in good yields.

Optimization of reaction conditions:

Table1:

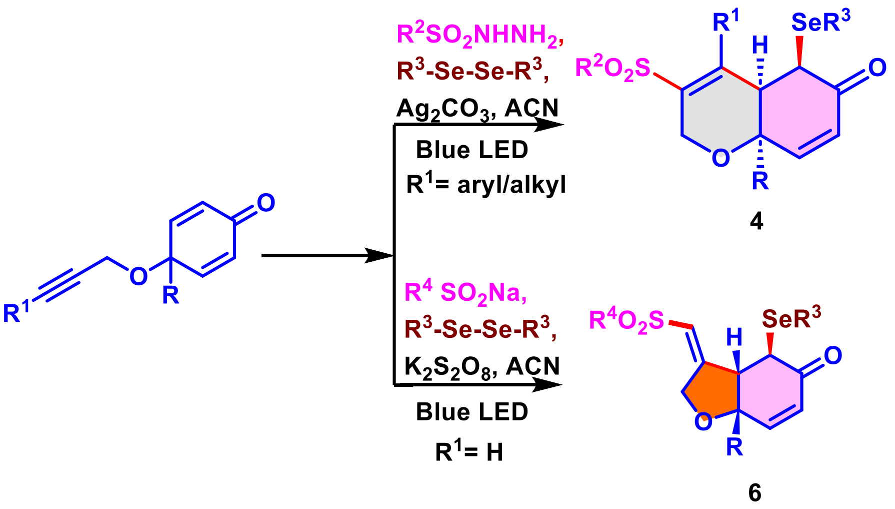

S.No.	Sulfonyl source	Photo Catalyst	R^1	Additive	$(PhSe)_2$ (mmol)	Oxidant	solvent	Yield	
								4a	6a
1	4-Me-Ph-SO_2NHNH_2	rose bengal	Ph	NaOAc (2.0eq)	0.5	-	ACN	29	-
2	4-Me-Ph-SO_2NHNH_2	Acr-Mes$^+$ClO$_4^-$	Ph	NaOAc (2.0eq)	0.5	-	ACN	35	-
3	4-Me-Ph-SO_2NHNH_2	eosin-y	Ph	NaOAc (2.0eq)	0.5	-	ACN	45	-
4	4-Me-Ph-SO_2NHNH_2	eosin-y	Ph	AgOAc (2.0eq)	0.5	-	ACN	51	-
5	4-Me-Ph-SO_2NHNH_2	-	Ph	AgOAc (2.0eq)	0.5	-	ACN	52	-
6	4-Me-Ph-SO_2NHNH_2	-	Ph	K_2CO_3 (2.0eq)	0.5	-	ACN	-	-
7	4-Me-Ph-SO_2NHNH_2	-	Ph	CsOAc (2.0eq)	0.5	-	ACN	10	-
8	4-Me-Ph-SO_2NHNH_2	-	Ph	Ag_2CO_3 (2.0eq)	0.5	-	ACN	90	-
9	4-Me-Ph-SO_2NHNH_2	-	Ph	Ag_2O (2.0eq)	0.5	-	ACN	88	-

10	4-Me-Ph-SO_2NHNH_2	-	Ph	DIPEA (2.0eq)	0.5	-	ACN	Trace	-
11	4-Me-Ph-SO_2NHNH_2	-	Ph	Cs_2CO_3 (2.0eq)	0.5	-	ACN	75	-
12	4-Me-Ph-SO_2NHNH_2	-	Ph	Ag_2CO_3 (0.1 eq)	0.5	-	ACN	Trace	-
13	4-Me-Ph-SO_2NHNH_2	-	Ph	Ag_2CO_3 (0.5 eq)	0.5	-	ACN	60	-
**14**	**4-Me-Ph-SO_2NHNH_2(1.0 eq)**	**-**	**Ph**	**Ag_2CO_3 (1.0 eq)**	**0.5**	**-**	**ACN**	**89**	**-**
15	4-Me-Ph-SO_2NHNH_2 (1.5 eq)	-	Ph	Ag_2CO_3 (1.0 eq)	0.5	-	ACN	80	-
16[a]	4-Me-Ph-SO_2NHNH_2	-	Ph	Ag_2CO_3 (1.0 eq)	0.5	-	ACN	-	-
17[b]	4-Me-Ph-SO_2NHNH_2	-	Ph	Ag_2CO_3 (1.0 eq)	0.5	-	ACN	65	-
18[c]	4-Me-Ph-SO_2NHNH_2	-	Ph	Ag_2CO_3 (1.0 eq)	0.5	-	ACN	Trace	-
19	4-Me-Ph-SO_2NHNH_2	-	Ph	-	0.5	-	ACN	Trace	-
20	4-Me-Ph-SO_2NHNH_2	-	Ph	Ag_2CO_3 (1.0 eq)	0.5	-	DMSO	35	-
21	4-Me-Ph-SO_2NHNH_2	-	Ph	Ag_2CO_3 (1.0 eq)	0.5	-	DMF	45	-
22	4-Me-Ph-SO_2NHNH_2	-	Ph	Ag_2CO_3 (1.0 eq)	0.5	-	Acetone	60	-
23	4-Me-Ph-SO_2NHNH_2	-	Ph	Ag_2CO_3 (1.0 eq)	0.5	-	ACN+H_2O	16	-
24	4-Me-Ph-SO_2NHNH_2	-	Ph	Ag_2CO_3 (1.0 eq)	0.5	-	2-Methyl THF	20	-
25	4-Me-Ph-SO_2NHNH_2	-	H	Ag_2CO_3 (1.0 eq)	0.5	-	ACN	-	-
26	4-Me-Ph-SO_2NHNH_2	-	H	Ag_2CO_3 (1.0 eq)	0.5	TBHP	ACN	-	-
27	4-Me-Ph-SO_2Na	-	H	-	0.5	TBHP	ACN	-	40
28	4-Me-Ph-SO_2Na	-	H	-	0.5	$(NH_4)_2S_2O_8$	ACN	-	75
**29**	**4-Me-Ph-SO_2Na(3.0eq)**	-	**H**	-	0.5	**$K_2S_2O_8$**	**ACN**	-	**88**
30	4-Me-Ph-SO_2Na	-	H	-	0.5	$Na_2S_2O_8$	ACN	-	62
31[a]	4-Me-Ph-SO_2Na	-	H	-	0.5	$K_2S_2O_8$	ACN	-	-
32[b]	4-Me-Ph-SO_2Na	-	H	-	0.5	$K_2S_2O_8$	ACN	-	55
33[c]	4-Me-Ph-SO_2Na	-	H	-	0.5	$K_2S_2O_8$	ACN	-	Trace
34	4-Me-Ph-SO_2Na	-	H	-	0.5	-	ACN	-	Trace
35	4-Me-Ph-SO_2Na(2.0eq)	-	H	-	0.5	$K_2S_2O_8$	ACN	-	75
36	4-Me-Ph-SO_2Na(1.0eq)	-	H	-	0.5	$K_2S_2O_8$	ACN	-	50

Standard condition for 6-exo trig cyclisation: Internal alkyne (1.0 eq), hydrazide (1.0 eq), diphenyldiselenide (0.5 eq) and Ag_2CO_3(1.0 eq) in 2 ml ACN under inert armosphere with 5 W Blue LED irrradiation;

Standard condition for 5-exo trigcyclisation: Terminal alkyne (1.0 eq), sodium phenyl sulfinate(3.0eq), diphenyldiselenide (0.5eq) and $K_2S_2O_8$(2.0 eq) in 2 ml ACN under inert armospherewith blue 5 W Blue LED irrradiation;

[a]Dark condition; [b]White LED's; [c]In sunlight

The results were summarized in the **Table-1**. At the outset of our preliminary investigation, the reaction of alkyne tethered cyclohexadienone**1a**, diphenyl diselenide and a sulfonyl reagent i.e, 4-methylbenzenesulfonyl hydrazide in the presence of photocatalyst (rose bengal), additive (NaOAc) and blue light emitting diode (LED) as the light source in acetonitrile. Pleasingly, this reaction affording the desired product in 29% yield **(Table1, entry-1)**. Encouraged by this result, other photocatalysts such as eosin-y, Acr-$Mes^+ClO_4^-$ **(Table1, entry-2&3)** were applied, and there is little bit improvement in the yield of the reaction. So, we further examined the reaction conditions with other additive AgOAc**(Table1, entry-4)**, which gave only 51% yield. Next, the reaction was performed without photocatalyst under visible light in the presence of AgOAc **(Table1, entry-5)** surprisingly, yield of the reaction was unchanged as compared to previous photocatalyst condition. It indicates there is no need of photocatalyst in this reaction. With this result, we further explored the reaction conditions with only additives such as K_2CO_3, CsOAc, Ag_2CO_3, Ag_2O, DIPEA, Cs_2CO_3**(Table1, entries 6-11)** and resulted Ag_2CO_3 was proven to be superior, affording the desired product **4a** in 89% yield. Then the loading of hydrazideand Ag_2CO_3 was investigated **(Table1, entries 12- 15)** and only 1.0 eq.of hydrazideand 1.0 eq. of Ag_2CO_3, furnishing the best yield. When other light source was applied, this reaction was complicated and provided **4a** in lower yield and no desired product was observed in the absence of either light or additive (**Table1, entries16-19).** Deep investigation of other solvents is also done and no other solvent gave the better yield than acetonitrile **(Table1, entries 20-24).** Therefore, we optimized the reaction conditions and found that entry number **14** is giving the best yield to access the 6- exo trig cyclized product. Surprisingly, the optimised condition failed to give the desired product with terminal alkyne **(Table1, entry 25).** The reaction conditions were then further explored with regard to the terminal alkyne with additional additives and oxidants with 4-methylbenzenesulfonyl hydrazide, but no desired product was appeared **(Table1**, **entry 26).** To our zeal, we changed the sulfonyl reagent from 4-methylbenzenesulfonyl

hydrazide to sodium *p*-toluene sulfinate and further investigated the reaction with oxidants **(Table1, entries 27-30).** To our delight, $K_2S_2O_8$underwent 5-exo trig cyclization and gave the desired 5,6 fused carbocycle with 88% yield (**Table1**, **entries 29)**. We further examined the reaction conditions with changing light source, in dark and without oxidant but we did not find any condition desirable (**Table1**, **entries 31-34).** Then the loading ofsulfinate is also investigated (**Table1**, **entries 35-36).**and 3.0 eqof sulfinate is furnishing the best yield.

Gram scale reaction and Synthetic Utility of 6-Exo Trig Radical Cascade Cyclization of Cyclohexadienones:

To further showcase the synthetic utility a gram scale reaction for synthesis of **4a** is conducted as shown in **Scheme 4.** The Internal alkyne (0.500 g,1.0 equiv), hydrazide (0.391g, 1.0 equiv.), diphenyldiselenide (0.327 g, 0.5 equiv) and Ag_2CO_3 (0.579 g,1.0 equiv) were weighed in to an oven dried 25 mL RBF followed by addition of ACN solvent under inert atmosphere. The reaction mixture was allowed to stir at room temperature for 6h under irradiation with 5 W blue LED’s and further purification was done by column chromatography to give the **4a** with 85% (0.75 g) yield. To the stirring solution of chalcogenated dihydrochromenones **4a** (0.050 g,1.0 equiv) in DCM add 3-chloroperbenzoic acid (0.018 g, 1.0 equiv) under inert atmosphere and the resulting mixture was stirred for 3h at room temperature then the reaction was diluted with 5 ml for water and organic layer was separated and washed with 5% sodium bicarbonate solution, the organic layer was dried (Na2SO4), filtered and concentrated under reduced pressure. The crude reaction mixture was purified by column chromatography to give deselenated product **7** with 75% (0.025 g) yield as shown in **Scheme 4**.

Gram scale reaction and Synthetic Utility of 5-Exo Trig Radical Cascade Cyclization of Cyclohexadienones:

To further showcase the synthetic utility a gram scale reactionfor synthesis of **6i** is conducted as shown in **Scheme 4**. The terminal alkyne (0.5 g, 1.0 equiv), sodium arylsulfinates (1.52 g, 3.0 equiv),diphenyldiselenide (0.481 g, 0.5 equiv) and $K_2S_2O_8$ (0.269 g, 2.0 equiv) were weighed into an oven dried 25 ml RBF followed by addition of ACN solvent under inert atmosphere. The reaction mixture was allowed to stir at room temperature for 6h under irradiation with 5 W blue LED’s and further purification was done by column chromatography to give the **6i** with 91% (1.2 g) yield. To the stirring solution of chalcogenated dihydrochromenones **6i** (0.05 g, 1.0equiv) in DCM add 3-chloroperbenzoic acid (0.019 g, 1.0 equiv) under inert atmosphere and the resulting

mixture was stirred for 3h at room temperature then the reaction was diluted with 5 mL for water and organic layer was separated and washed with 5% sodium bicarbonate solution, the organic layer was dried (Na_2SO_4), filtered and concentrated under reduced pressure. The crude reaction mixture was purified by column chromatography to give deselenated product **8** in 80% (0.026 g) yield as depicted in **Scheme 4**.

X-ray Crystallography.

X-ray data for the compound KB359_0m was collected at room temperature on a Bruker D8 QUEST instrument with an IμS Mo microsource (λ = 0.7107 A) and a PHOTON-III detector. The raw data frames were reduced and corrected for absorption effects using the Bruker Apex 3 software suite programs [2]. The structure was solved using intrinsic phasing method [3] and further refined with the SHELXL [3] program and expanded using Fourier techniques. Anisotropic displacement parameters were included for all non-hydrogen atoms. All H atoms were positioned geometrically and treated as riding on their parent C atoms [C-H = 0.93-0.97 Å, O-H = 0.82 Å and $U_{iso}(H) = 1.5U_{eq}(C, O)$ for methyl H or $1.2U_{eq}(C, N)$ for other H atoms].

Crystal structure determination of [KB359_0m] (4a)

Crystal Data for $C_{29}H_{26}O_4SSe$ (M =549.52 g/mol): monoclinic, space group $P2_1/c$ (no. 14), a = 15.124(3) Å, b = 13.218(3) Å, c = 14.668(2) Å, β = 118.370(7)°, V = 2580.2(9) $Å^3$, Z = 4, T = 294(2) K, μ(MoKα) = 1.569 mm^{-1}, $Dcalc$ = 1.415 g/cm^3, 36359 reflections measured (4.41° ≤ 2Θ ≤ 50°), 4462 unique (R_{int} = 0.0556, R_{sigma} = 0.0310) which were used in all calculations. The final R_1 was 0.0282 (I > 2σ(I)) and wR_2 was 0.0756 (all data). CCDC 2240129 contains supplementary Crystallographic data for the structure. These data can be obtained free of charge at www.ccdc.cam.ac.uk/conts/retrieving.html [or from the Cambridge Crystallographic Data Centre (CCDC), 12 Union Road, Cambridge CB2 1EZ, UK; fax: +44(0) 1223 336 033; email: deposit@ccdc.cam.ac.uk].

Crystal structure determination of [KB402_0m] (4g)

Crystal Data for $C_{23}H_{22}O_4SSe$ (M =473.42 g/mol): monoclinic, space group $P2_1/c$ (no. 14), a = 9.886(4) Å, b = 18.236(8) Å, c = 12.143(5) Å, β = 100.541(7)°, V = 2152.2(16) $Å^3$, Z = 4, T = 294(2) K, μ(MoKα) = 1.868 mm^{-1}, $Dcalc$ = 1.461 g/cm^3, 32190 reflections measured (4.75° ≤ 2Θ ≤ 54.994°), 4936 unique (R_{int} = 0.0618, R_{sigma} = 0.0551) which were used in all calculations. The final R_1 was 0.0414 (I > 2σ(I)) and wR_2 was

0.0940 (all data). CCDC 2240130 contains supplementary Crystallographic data for the structure. These data can be obtained free of charge at www.ccdc.cam.ac.uk/conts/retrieving.html [or from the Cambridge Crystallographic Data Centre (CCDC), 12 Union Road, Cambridge CB2 1EZ, UK; fax: +44(0) 1223 336 033; email: deposit@ccdc.cam.ac.uk].

Crystal structure determination of [KB491_0m] (6a)

Crystal Data for $C_{24}H_{24}O_4SSe$ (M =487.45 g/mol): monoclinic, space group $P2_1/c$ (no. 14), a = 11.2466(2) Å, b = 14.9374(3) Å, c = 13.7299(3) Å, β = 98.8260(10)°, V = 2279.24(8) Å^3, Z = 4, T = 294(2) K, μ(MoKα) = 1.766 mm^{-1}, $Dcalc$ = 1.421 g/cm^3, 24572 reflections measured (5.148° ≤ 2Θ ≤ 49.998°), 4010 unique (R_{int} = 0.0572, R_{sigma} = 0.0492) which were used in all calculations. The final R_1 was 0.0454 (I > 2σ(I)) and wR_2 was 0.1142 (all data). CCDC 2240131 contains supplementary Crystallographic data for the structure. These data can be obtained free of charge at www.ccdc.cam.ac.uk/conts/retrieving.html [or from the Cambridge Crystallographic Data Centre (CCDC), 12 Union Road, Cambridge CB2 1EZ, UK; fax: +44(0) 1223 336 033; email: deposit@ccdc.cam.ac.uk].

Figure Captions

Fig.1. A view of **KB402**, showing the atom-labelling scheme. Displacement ellipsoids are drawn at the 30% probability level and H atoms are represented by circles of arbitrary radii(4a).

Fig.2. A view of **KB359**, showing the atom-labelling scheme. Displacement ellipsoids are drawn at the 30% probability level and H atoms are represented by circles of arbitrary radii(4g).

Fig.1. A view of **KB491**, showing the atom-labelling scheme. Displacement ellipsoids are drawn at the 30% probability level and H atoms are represented by circles of arbitrary radii(6a).

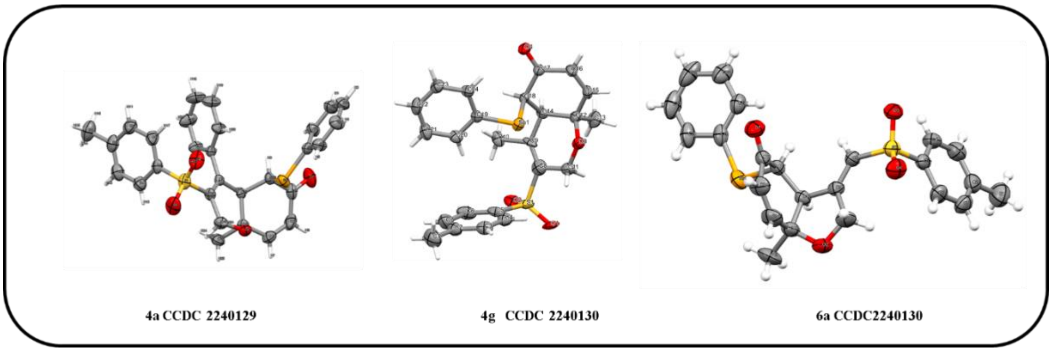

Note: Peaks corresponding 2xCH were found to be missing in the ^{13}C NMR of the products most probably due to them being broad. This could be due to the restricted rotation around the tetra substituted alkene and this type of observations was also reported by Volla[4] as well as Lam and co-workers.[5]

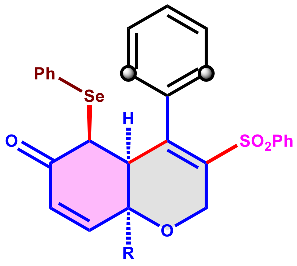

Signals corresponding to these carabons were missing due to being broad

Mechanism

In the intermediate **II**, there are two possible isomers (with respect to hydrogen at the radical), in which the hydrogen atom of one of isomer (**II-A**) is in the same plane with the phenyl group. This may cause the steric repulsion in this isomer and the formation of intermediate **II-A** may be disfavored. While in the case of other isomer (**II-B**), these repulsions are avoided because of hydrogen atom and phenyl group are in opposite plane. This favors the formation of intermediate **II-B**, which further helps in the stereo selective attack of aryl selenium radical and furnishes the final product **4**.

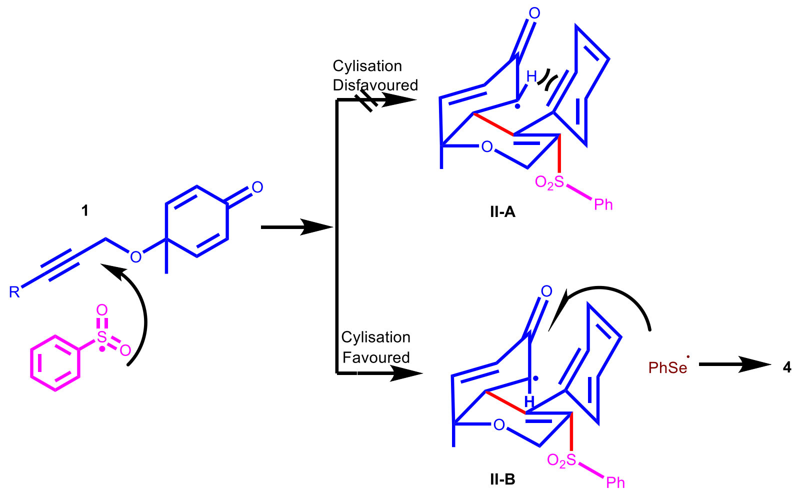

3. References:

1. (a) Hexum, J. K.; Tello-Aburto, T.; Struntz,N.B.; Harned A. M.;Harki, D. A.Bicyclic Cyclohexenones as Inhibitors of NF-κB Signaling.*ACS Med. Chem. Lett*. **2012**, *3*, 459-464. (b) Fukui, Y.; Liu, P.; Liu, Q.; He, Z.-T.; Wu, N.-Y.; Tian P.; G.-Q. Lin, Tunable Arylative Cyclization of 1,6-Enynes Triggered by Rhodium (III)-Catalyzed C–H Activation. *J. Am. Chem. Soc.*,**2014,***136*, 15607-15614.
2. Bruker (2016). APEX3, SAINT and SADABS. Bruker AXS, Inc., Madison, Wisconsin, USA.
3. Sheldrick G. M. (2015**)** ActaCrystallogr C71: 3-8.
4. Nair, A. M.; Halder, I.; Volla, C. M. R. A metal-free four-component sulfonylation, Giese cyclization, selenylation cascade via insertion of sulfur dioxide. *Chem. Commun.* **2022**, *58*, 6950-6953.
5. (a) Zhu, S.; Pathigoolla, A.; Lowe, G.; Walsh, D. A.; Cooper, M.; Lewis, W.; Lam, H. W. *Chem. Eur. J.* **2017**, *23*, 17598-17604.

^{1}H NMR (400 MHz, $CDCl_3$) of 4a

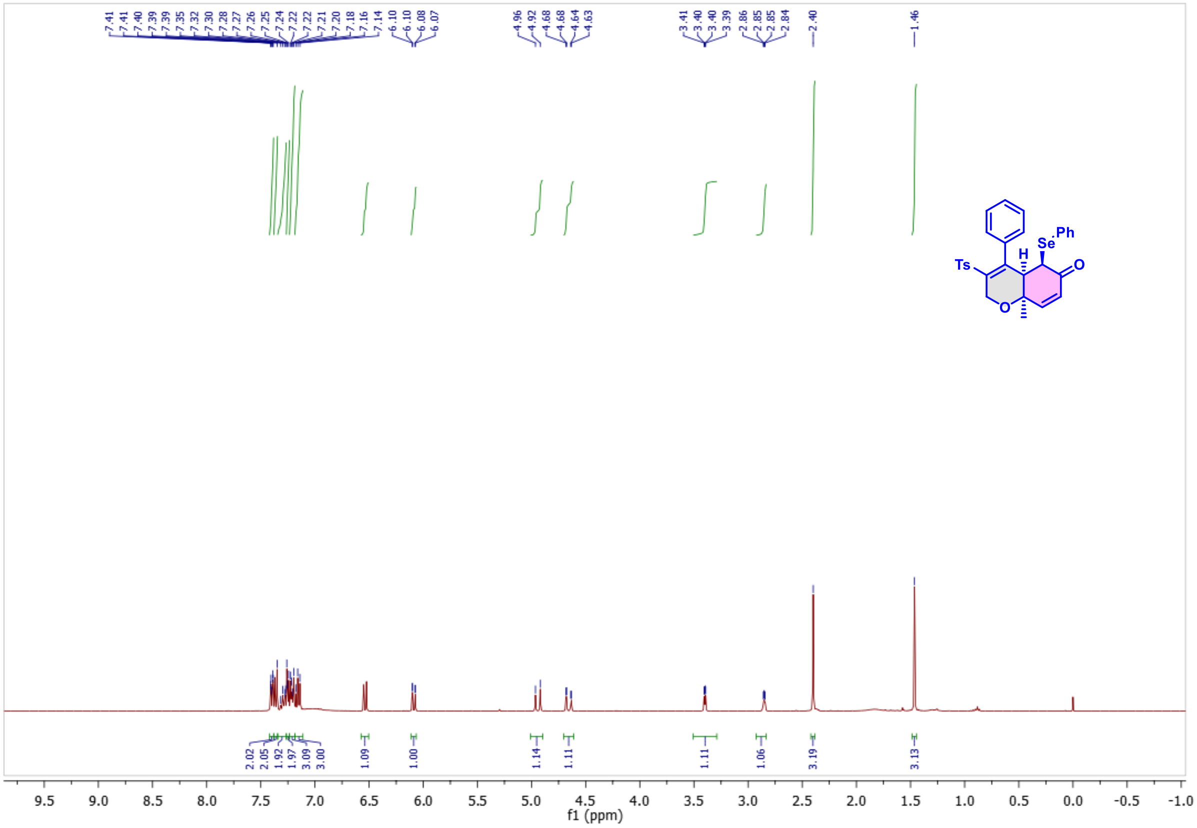

^{13}C{^{1}H} NMR (100 MHz, $CDCl_3$) of 4a

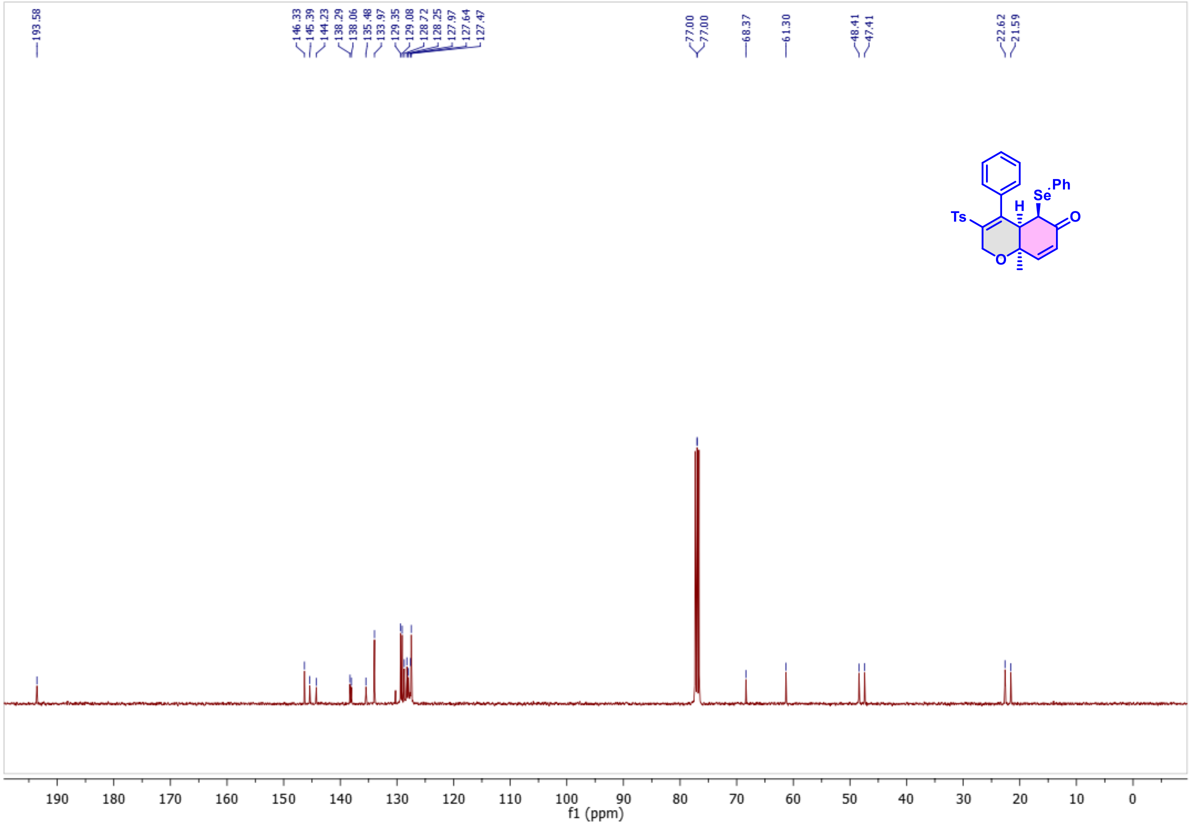

^{1}H NMR (400 MHz, CDCl$_3$) of 4b

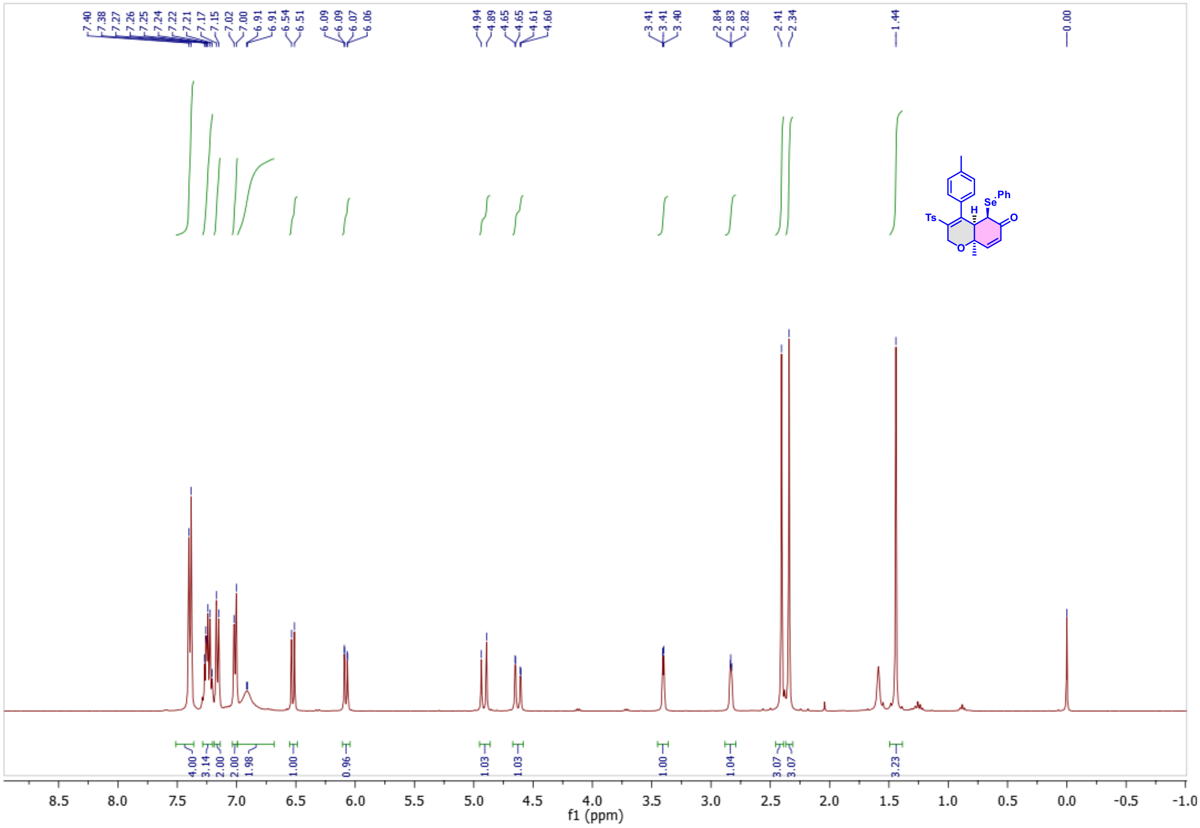

^{13}C{^{1}H} NMR (125 MHz, CDCl$_3$) of 4b

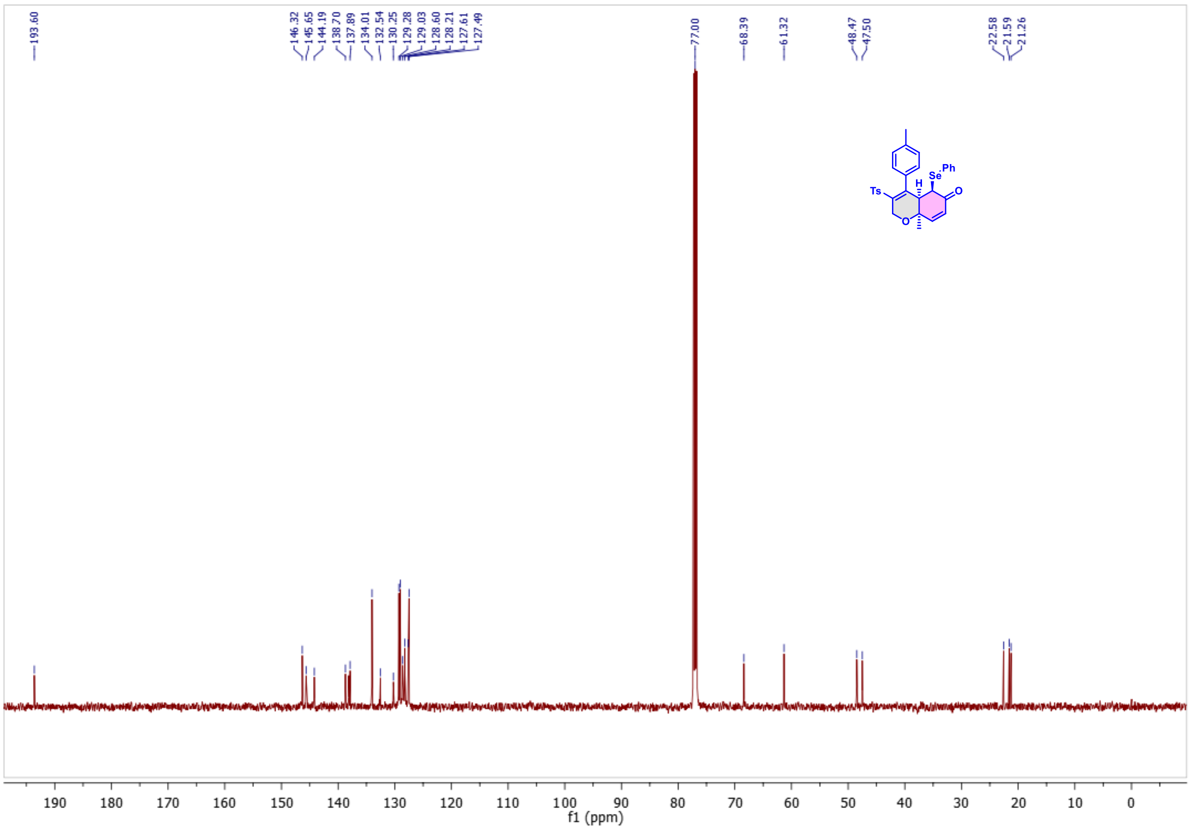

^{1}H NMR (400 MHz, $CDCl_3$) of 4c

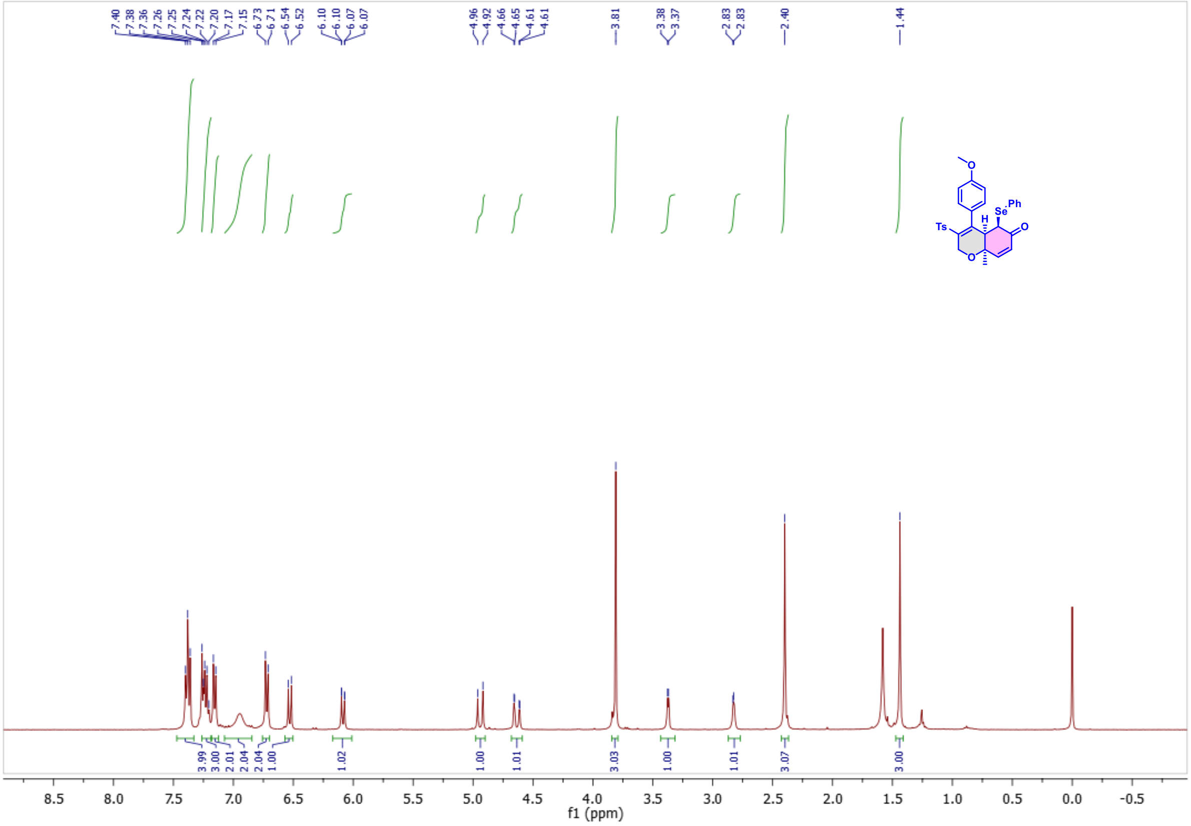

$^{13}C\{^{1}H\}$ NMR (100 MHz, $CDCl_3$) of 4c

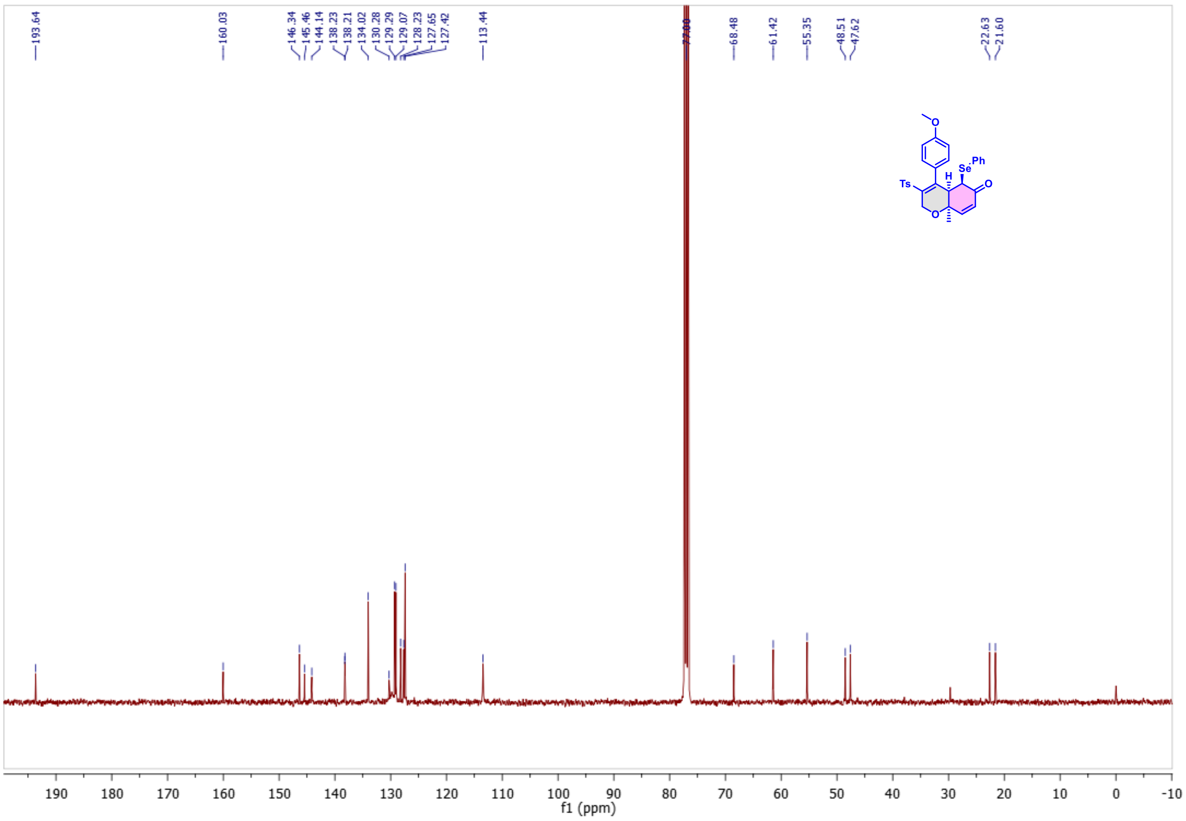

^{1}H NMR (500 MHz, $CDCl_3$) of 4d

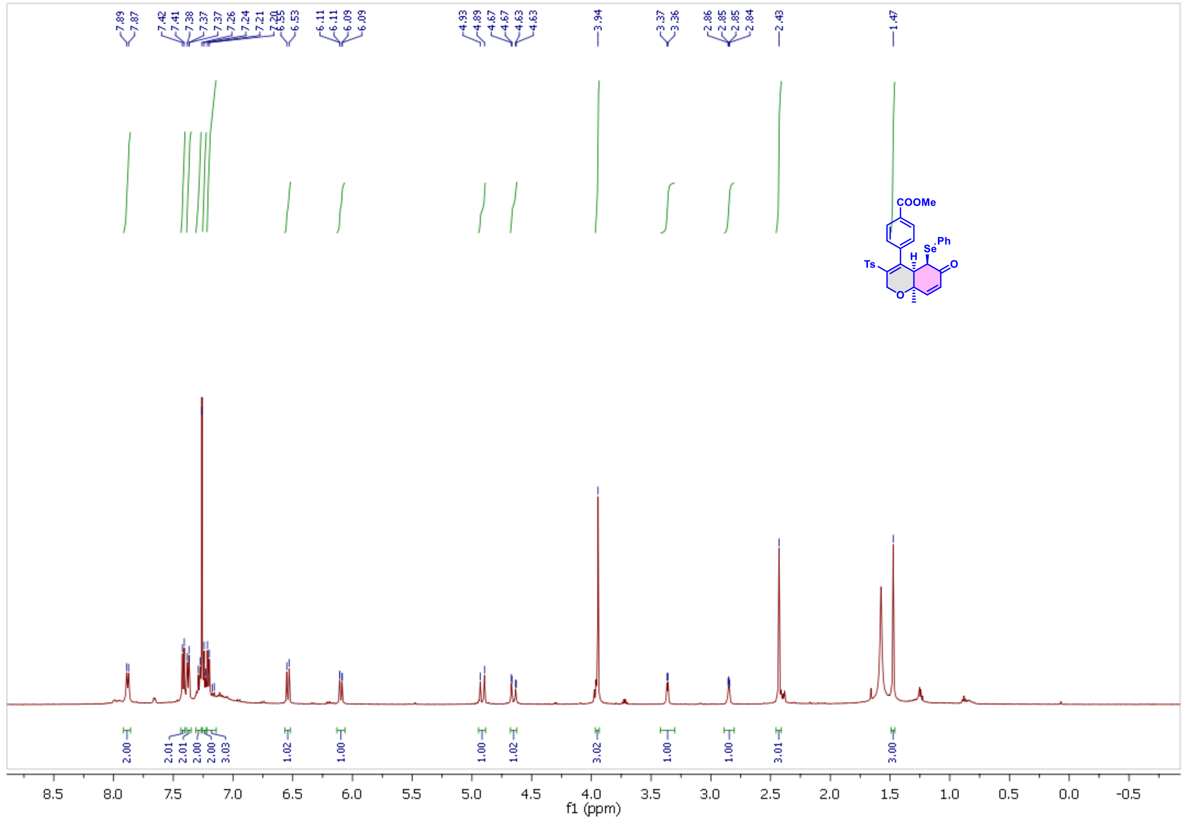

$^{13}C\{^{1}H\}$ NMR (100 MHz, $CDCl_3$) of 4d

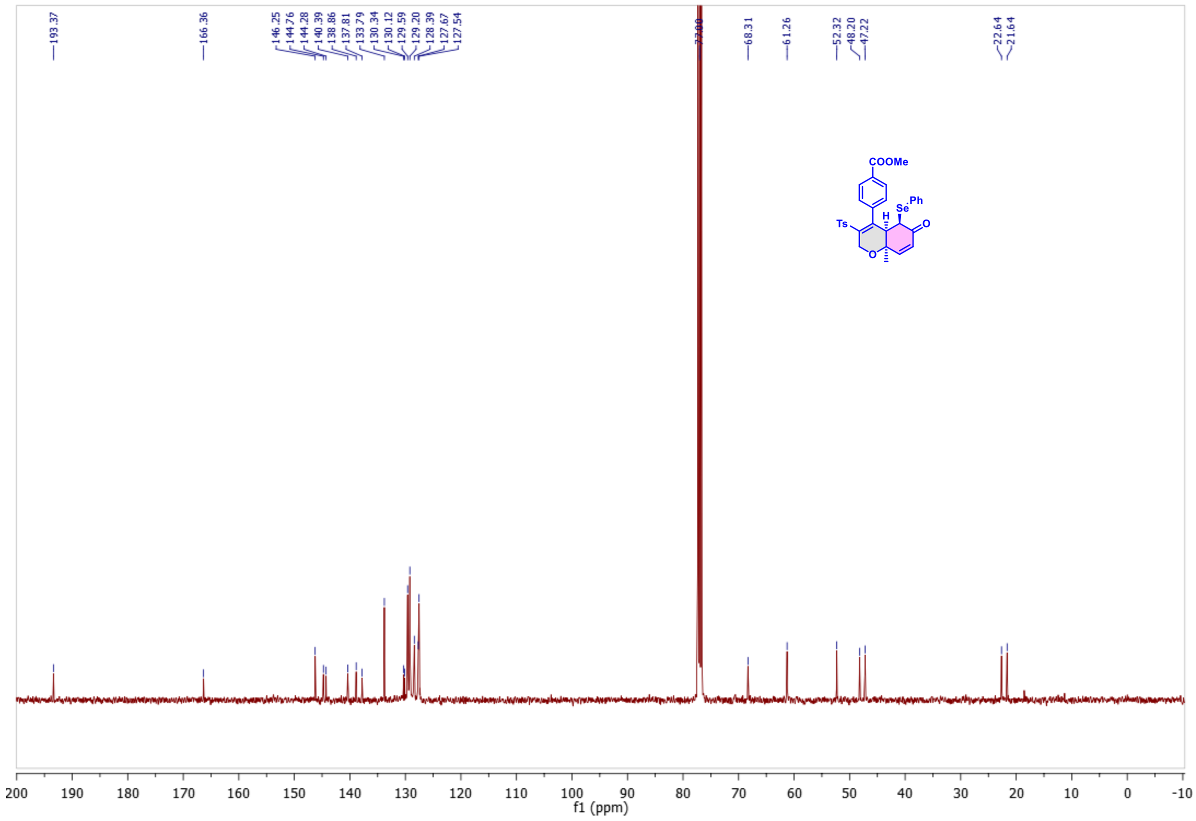

^{1}H NMR (400 MHz, $CDCl_3$) of 4e

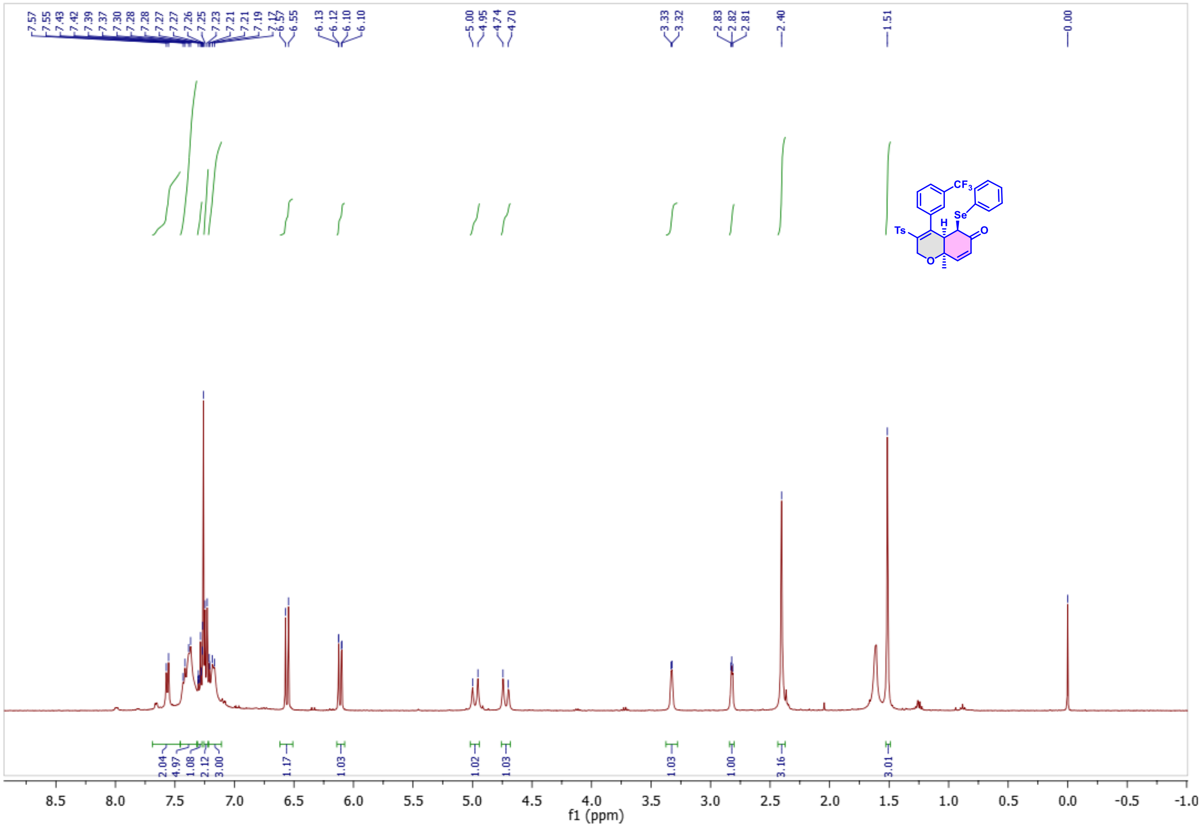

^{13}C{^{1}H} NMR (100 MHz, $CDCl_3$) of 4e

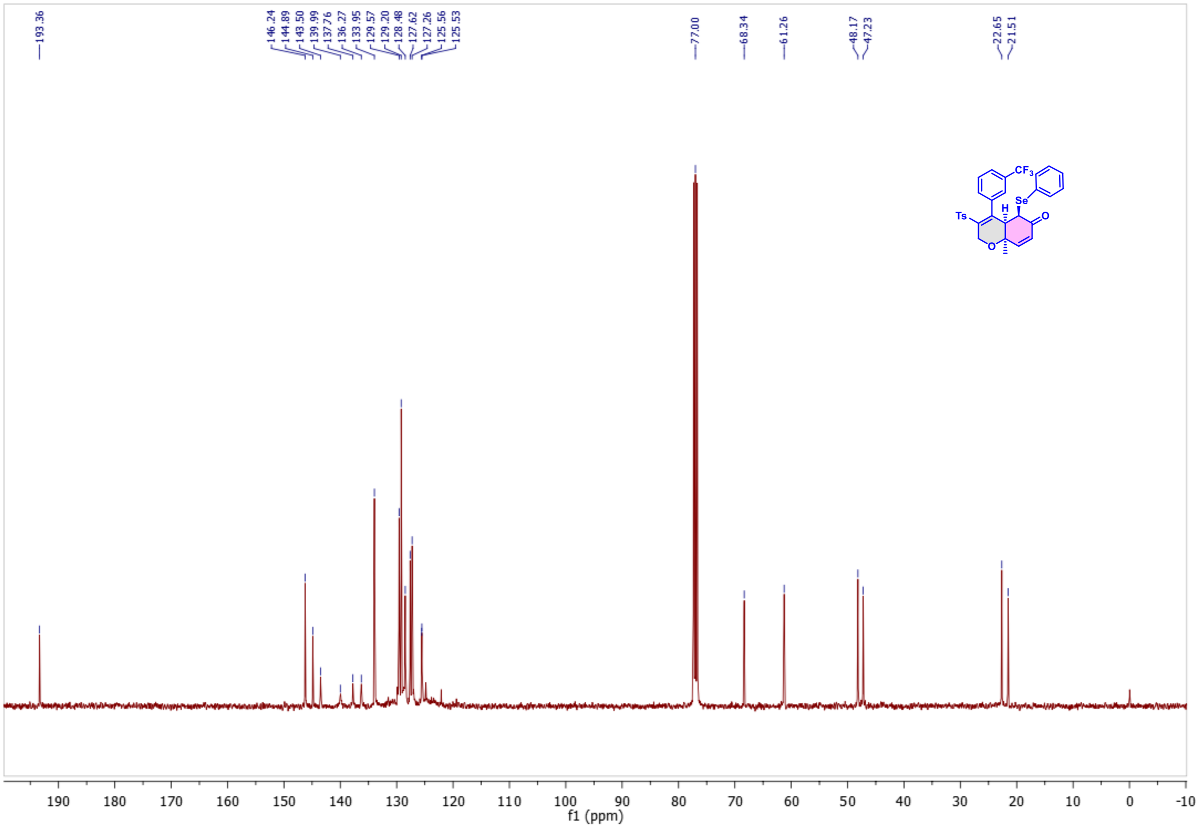

^{1}H NMR (400 MHz, $CDCl_3$) of 4f

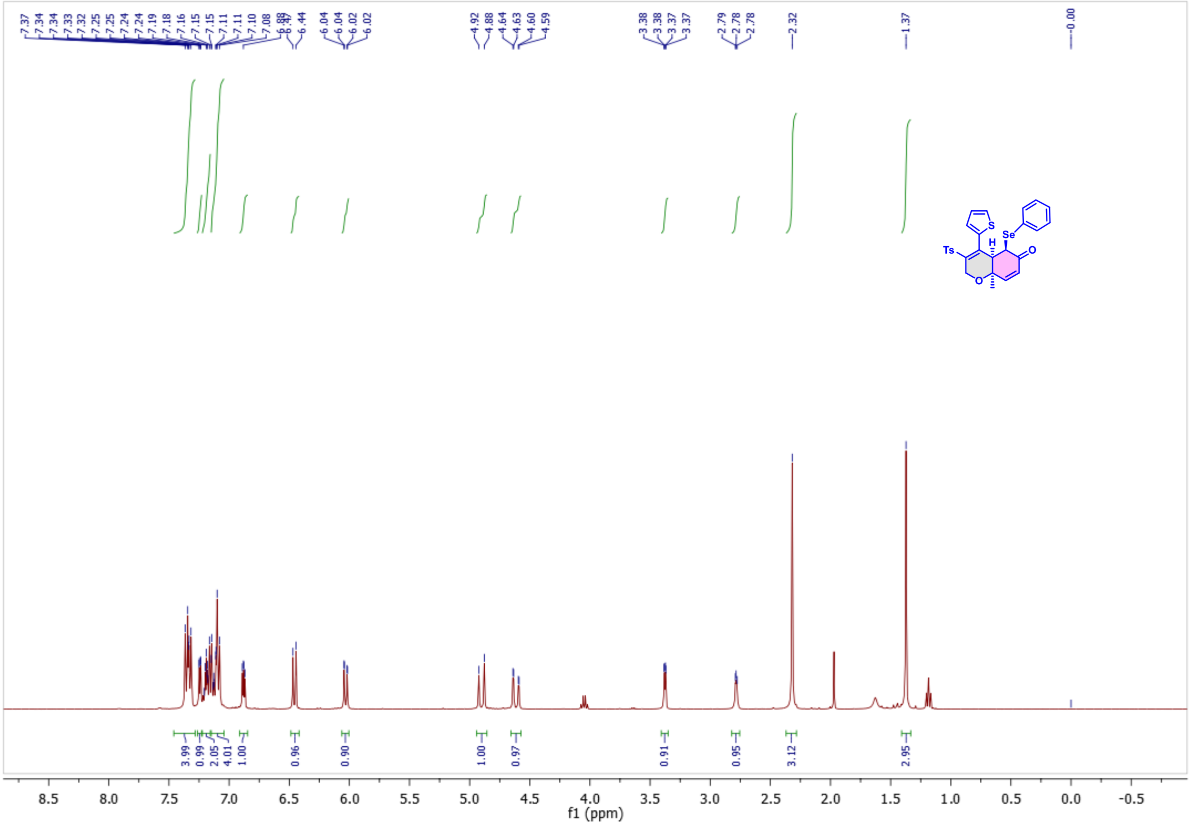

$^{13}C\{^{1}H\}$ NMR (100 MHz, $CDCl_3$) of4f

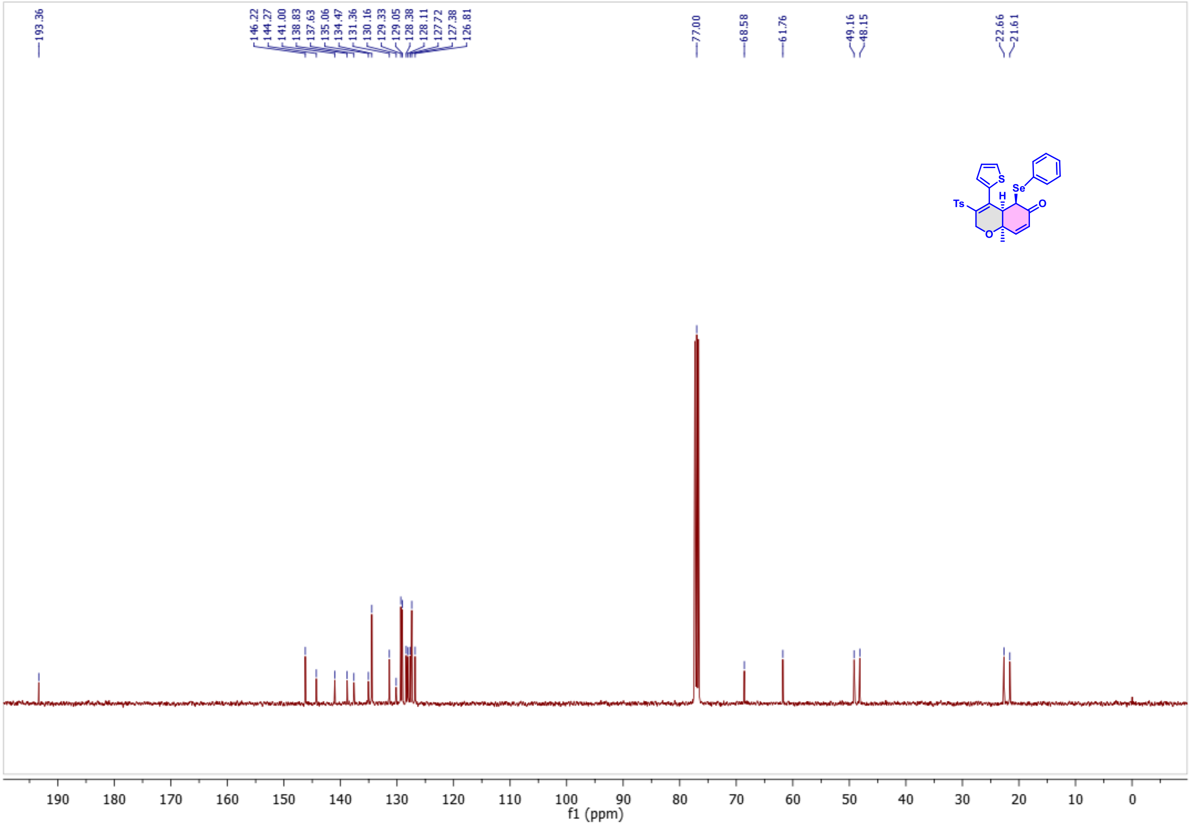

^{1}H NMR (500 MHz, $CDCl_3$) of 4g

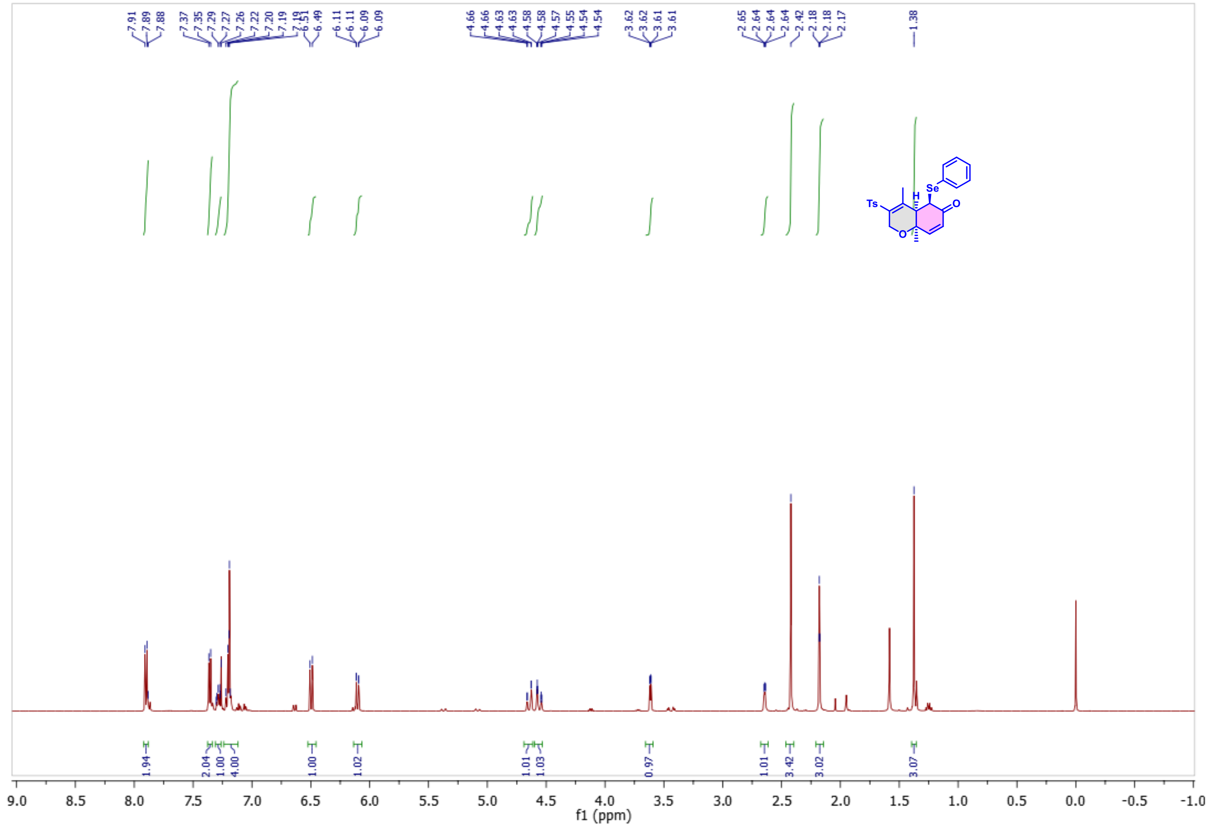

$^{13}C\{^{1}H\}$ NMR (100 MHz, $CDCl_3$) of 4g

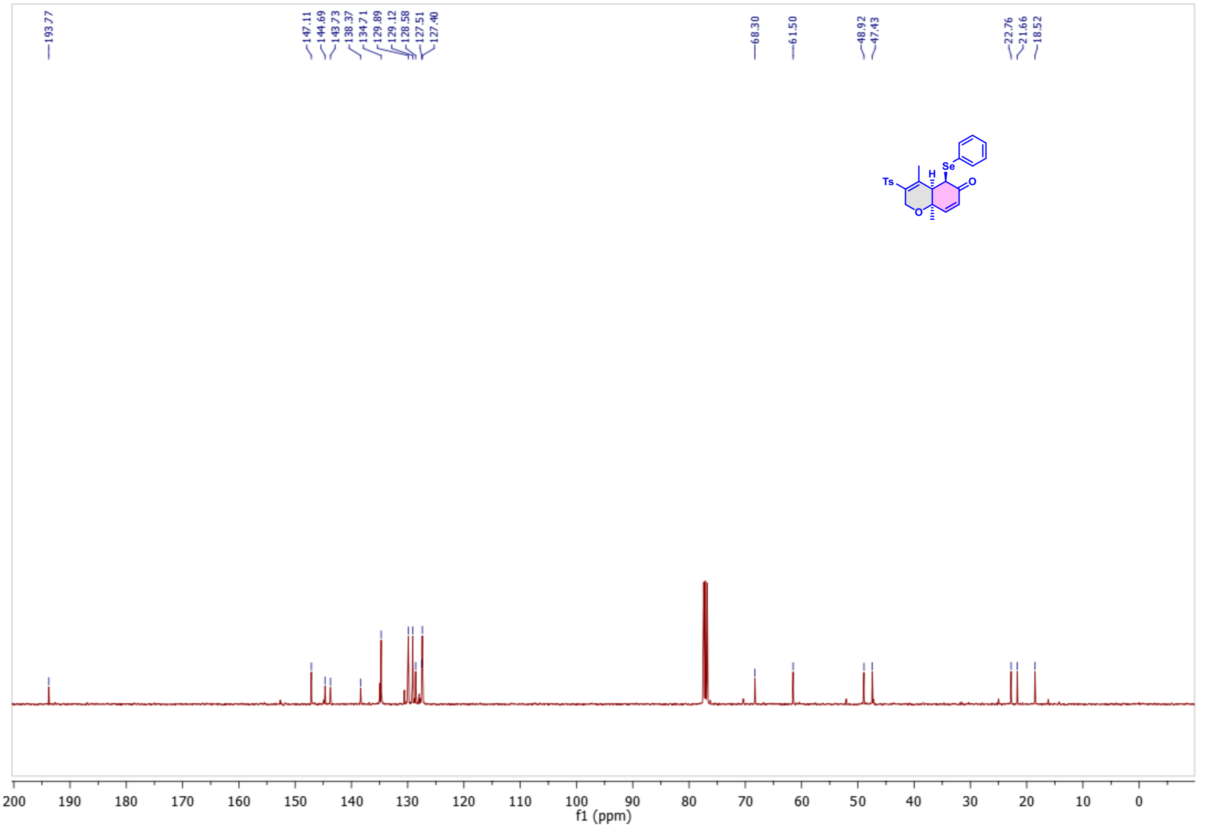

^{1}H NMR (400 MHz, CDCl3) of 4h

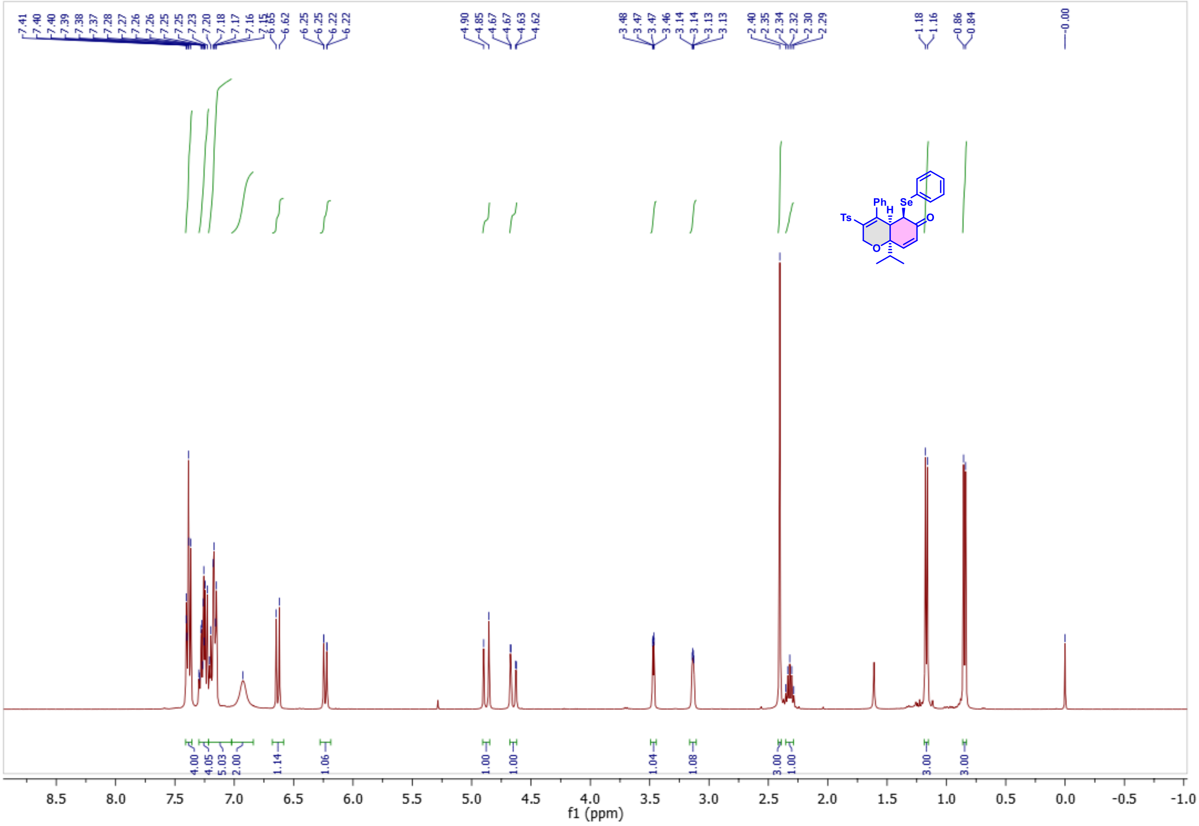

^{13}C{^{1}H} NMR (125 MHz, CDCl3) of 4h

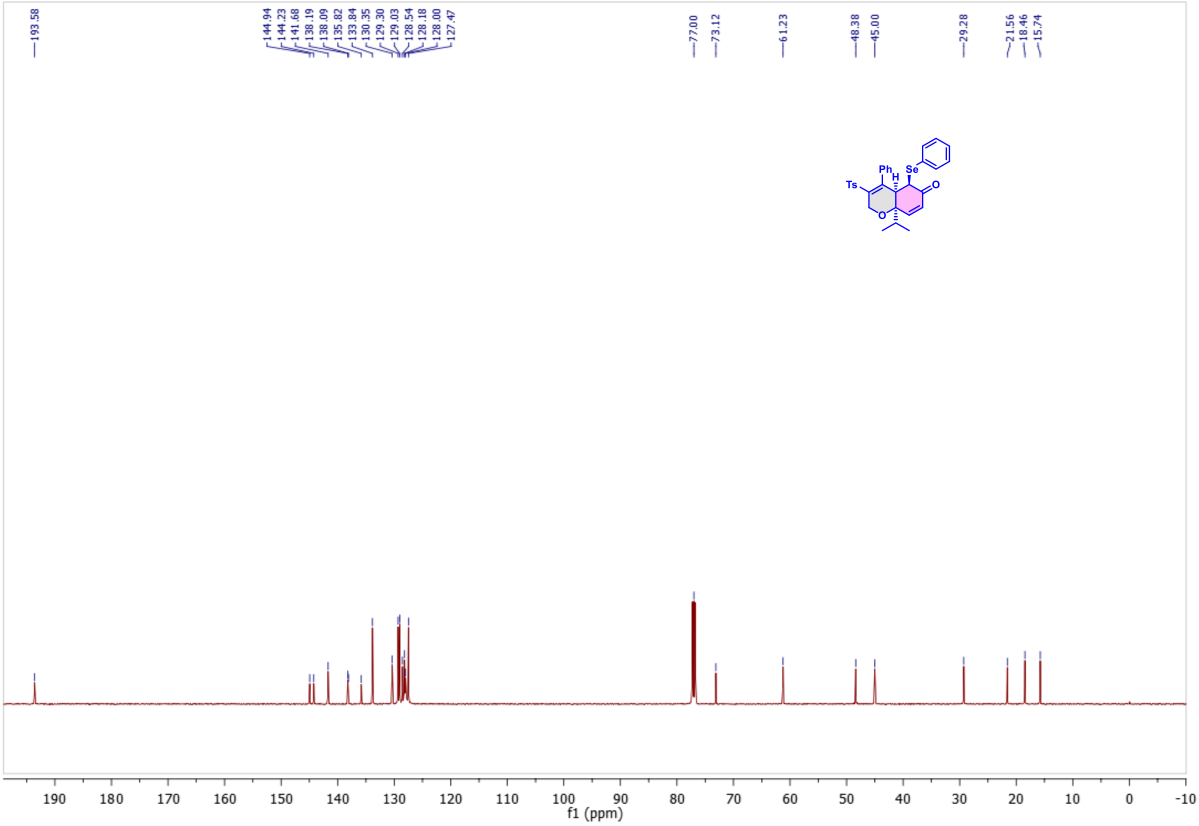

^{1}H NMR (500 MHz, $CDCl_3$) of 4i

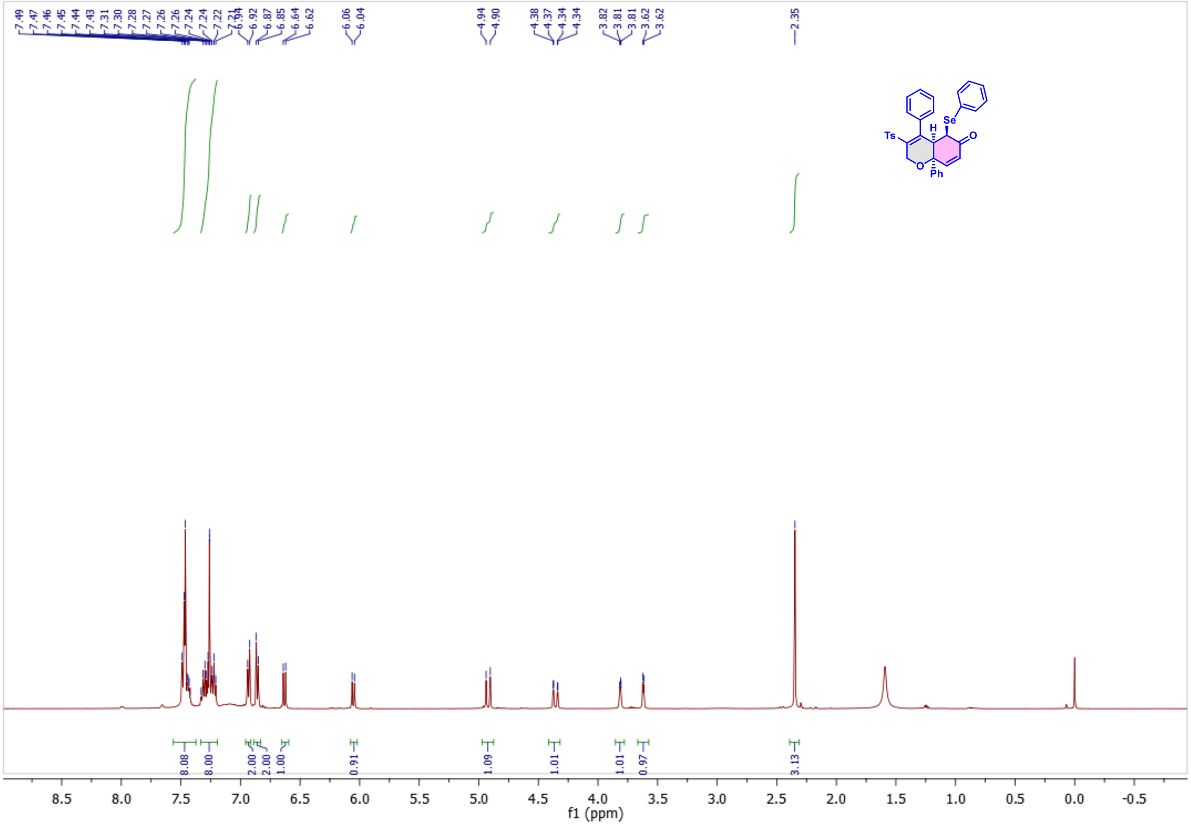

$^{13}C\{^{1}H\}$ NMR (100 MHz, $CDCl_3$) of 4i

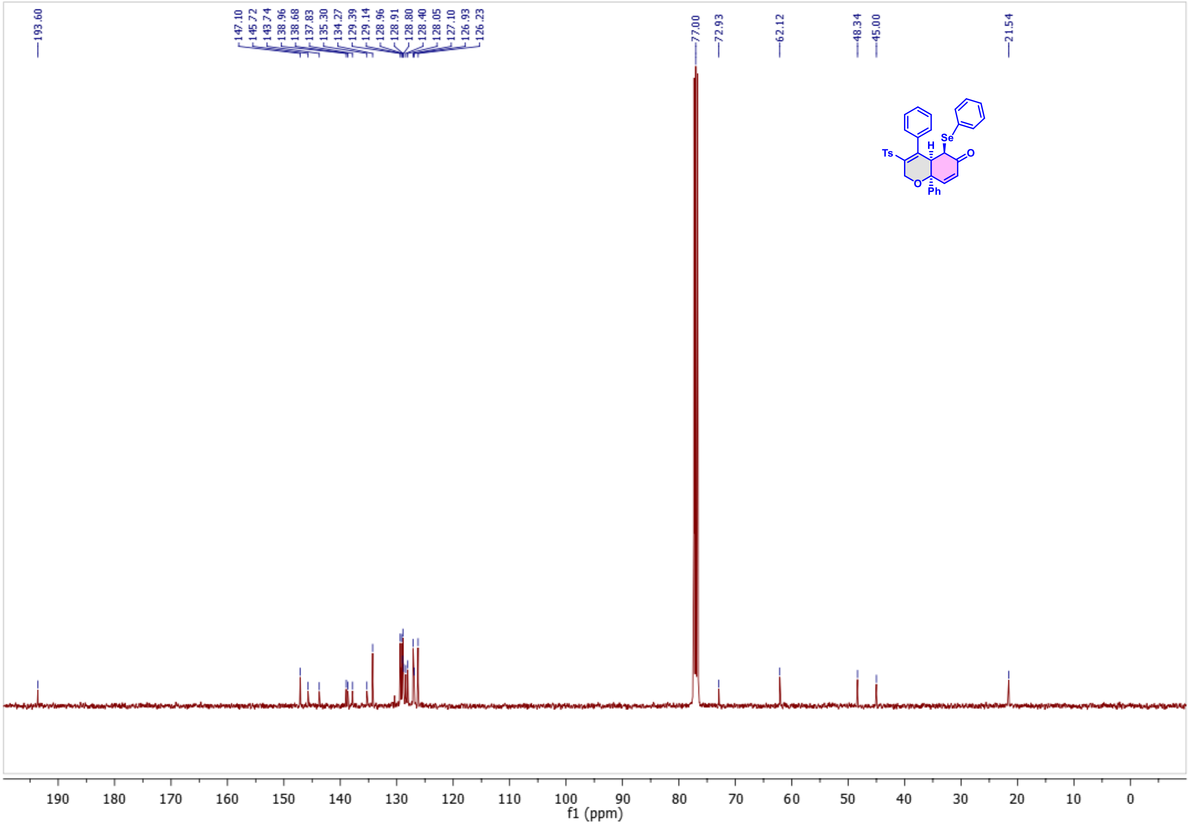

^{1}H NMR (500 MHz, $CDCl_3$) of 4j

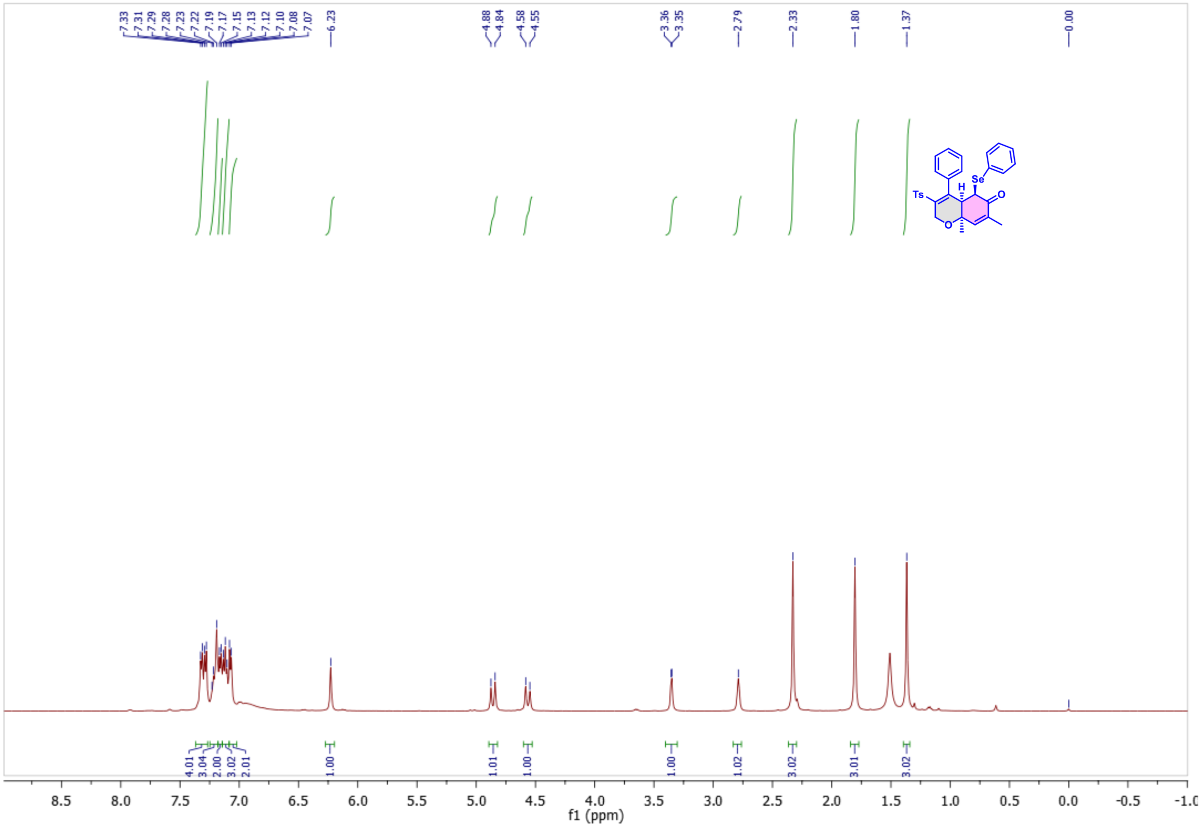

$^{13}C\{^{1}H\}$ NMR (125 MHz, $CDCl_3$) of 4j

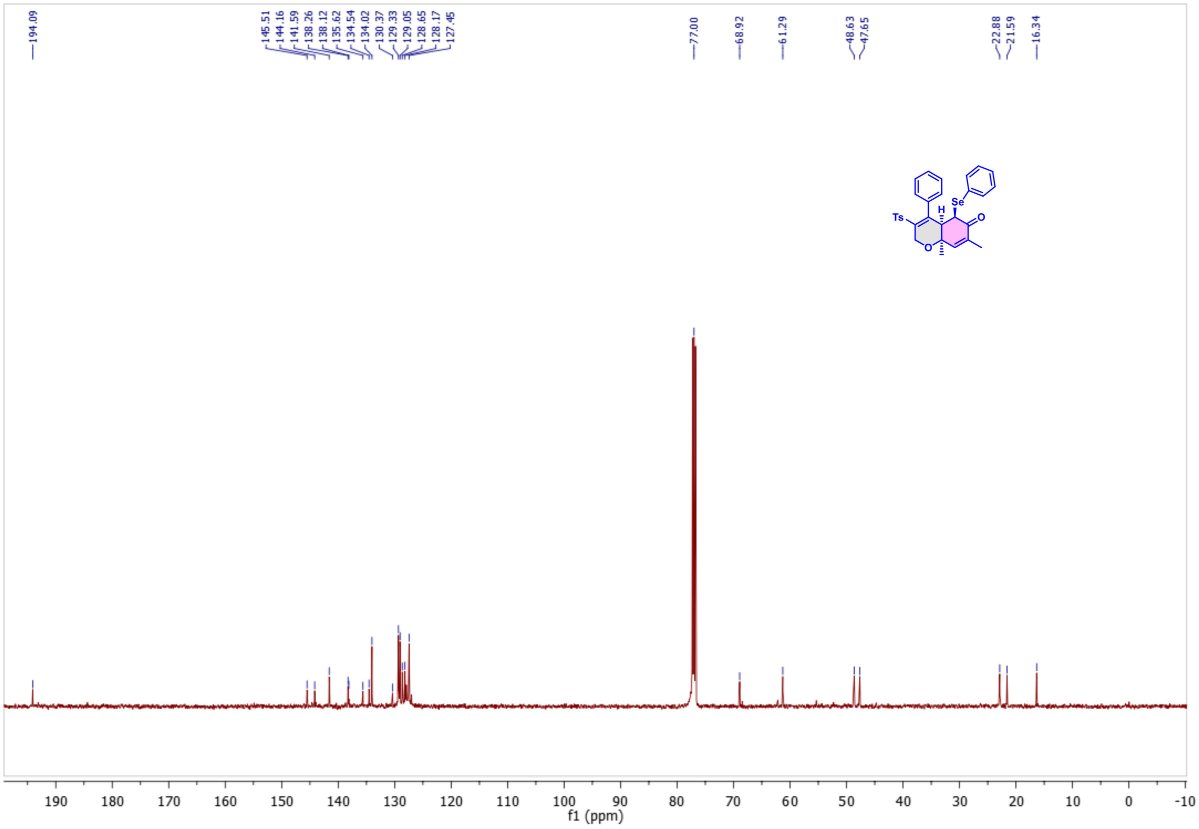

^{1}H NMR (400 MHz, $CDCl_3$) of 4k

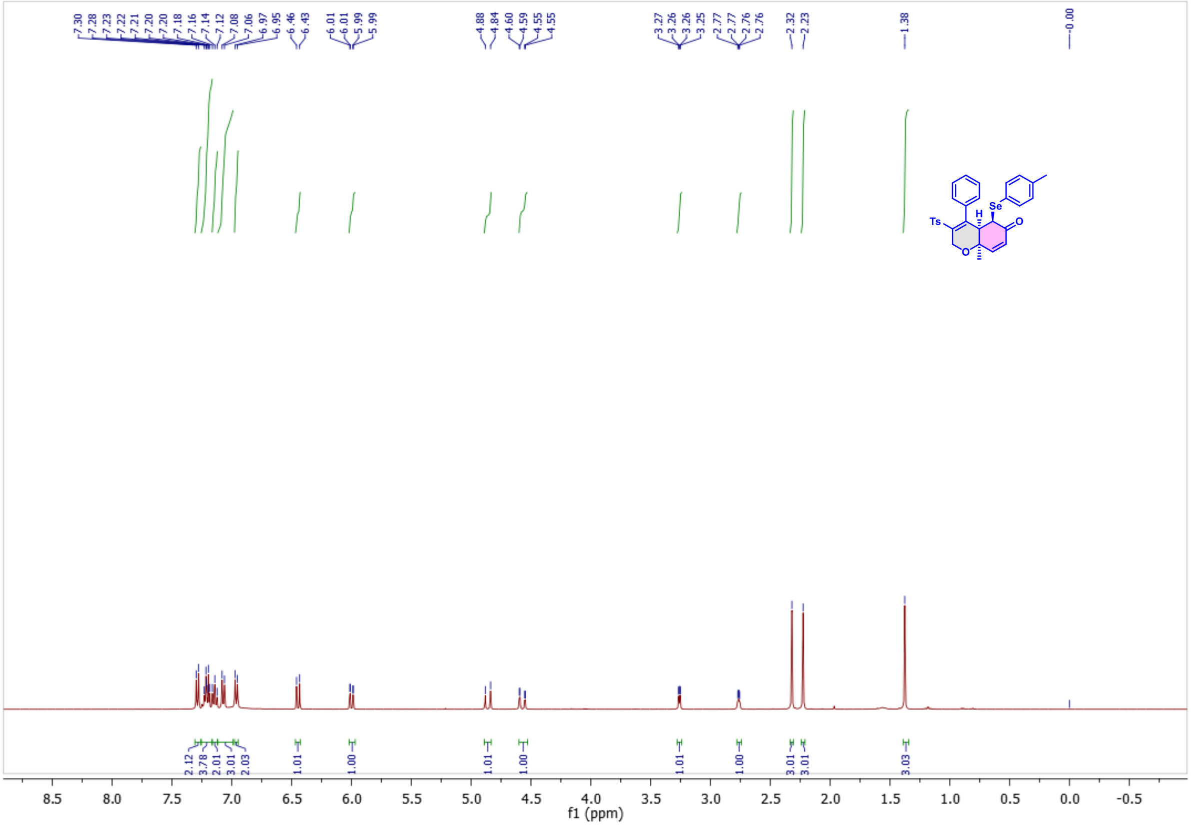

$^{13}C\{^{1}H\}$ NMR (125 MHz, $CDCl_3$) of 4k

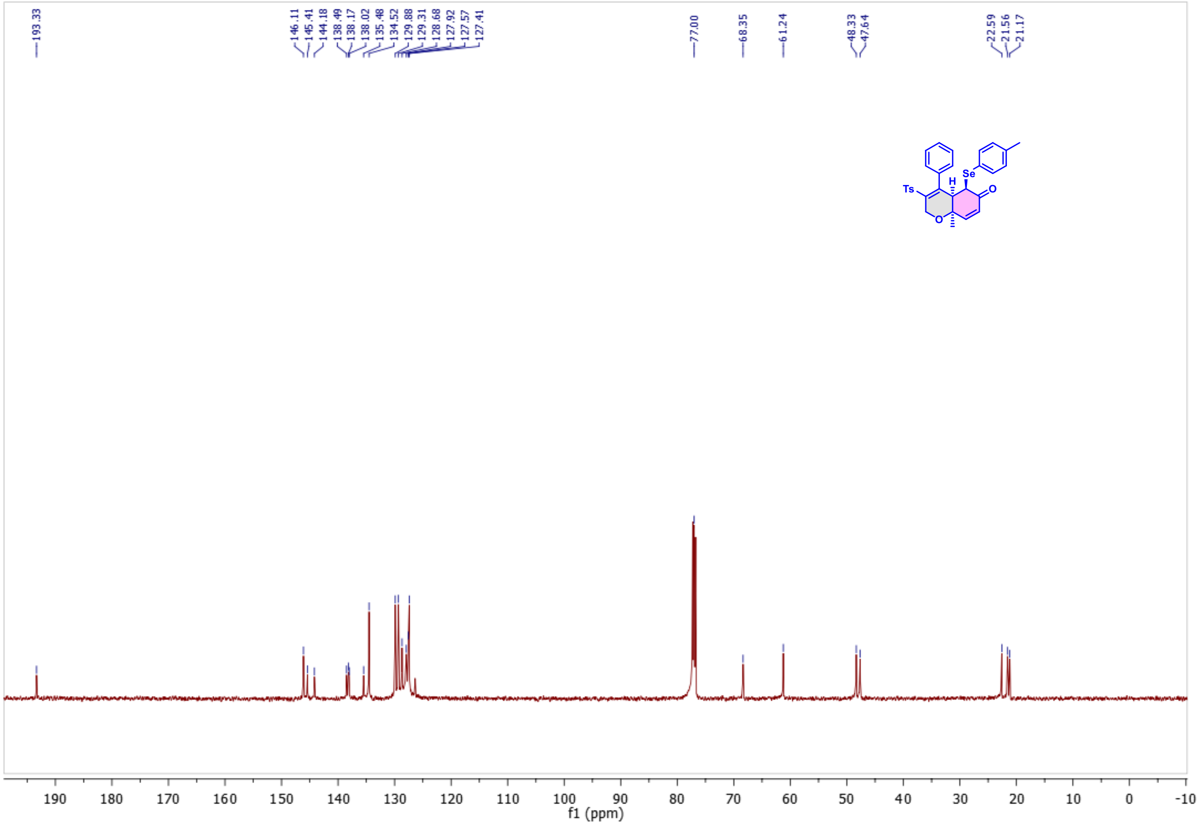

^{1}H NMR (500 MHz, $CDCl_3$) of 4l

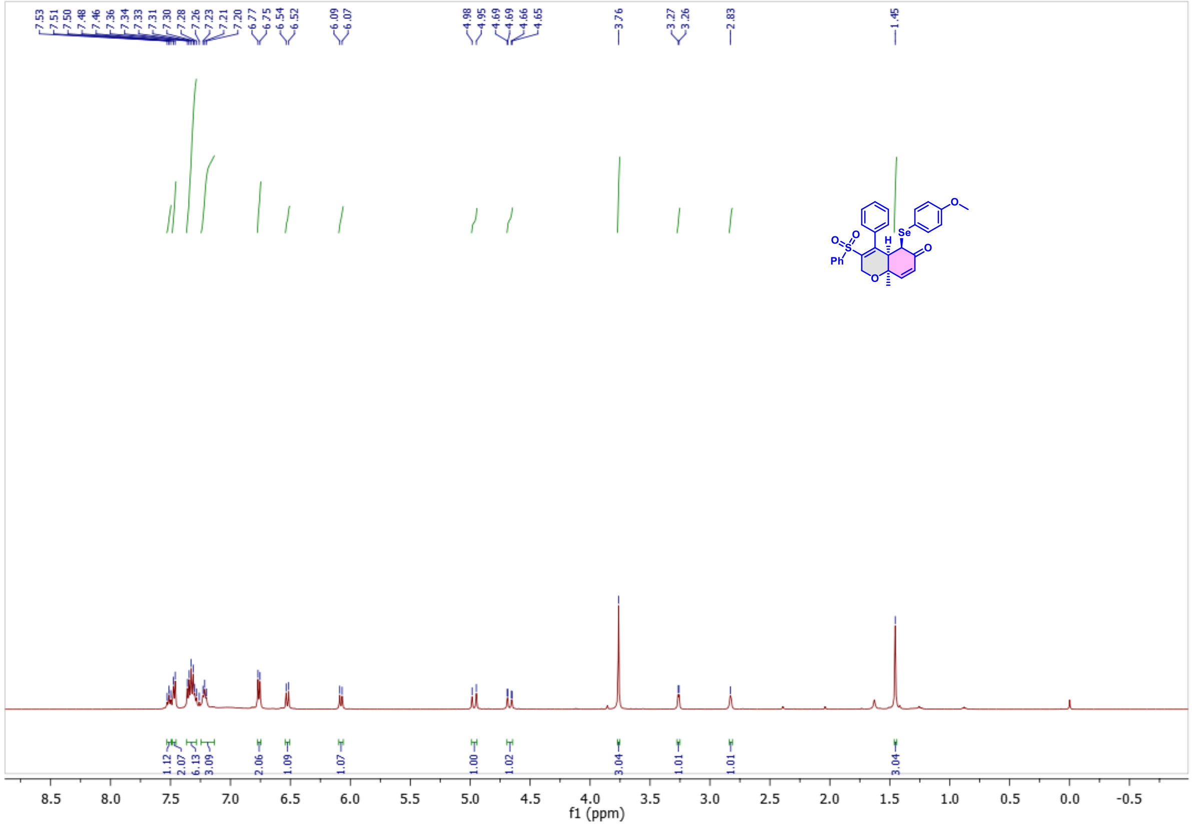

$^{13}C\{^{1}H\}$ NMR (100 MHz, $CDCl_3$) of 4l

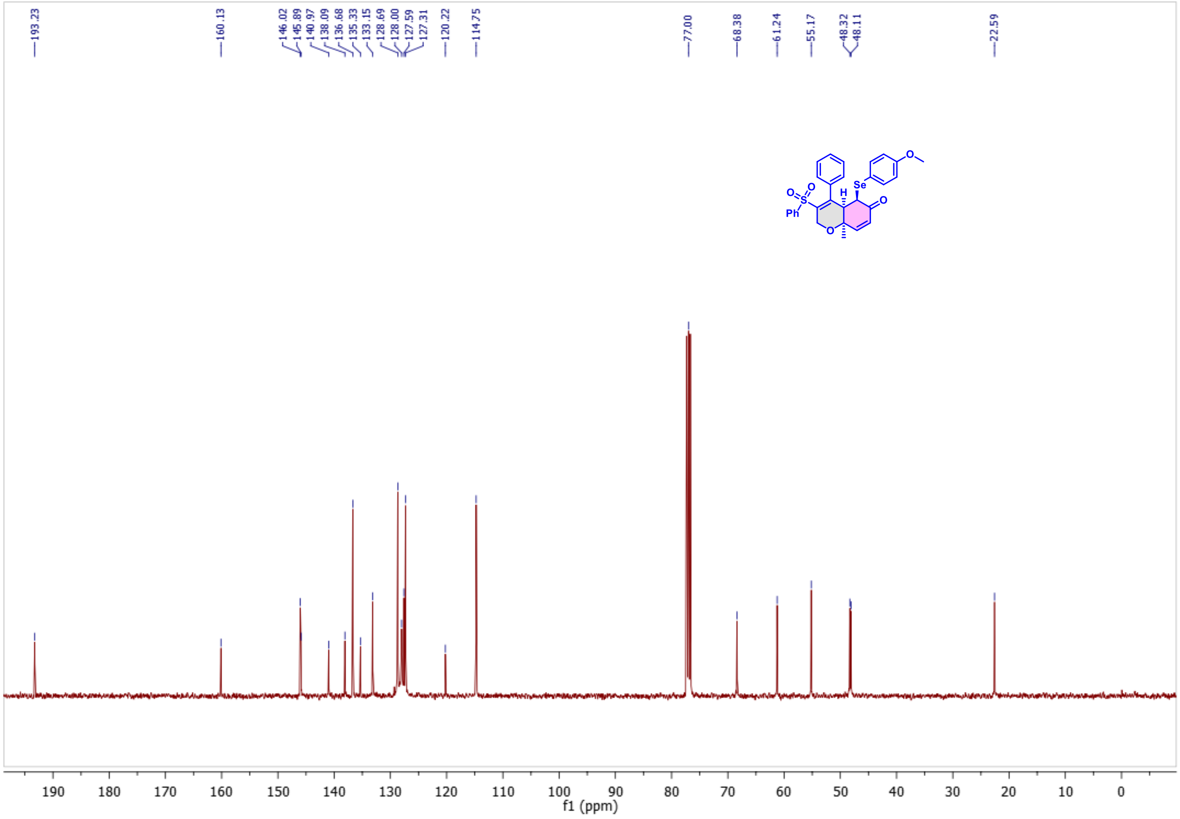

^{1}H NMR (400 MHz, $CDCl_3$) of 4m

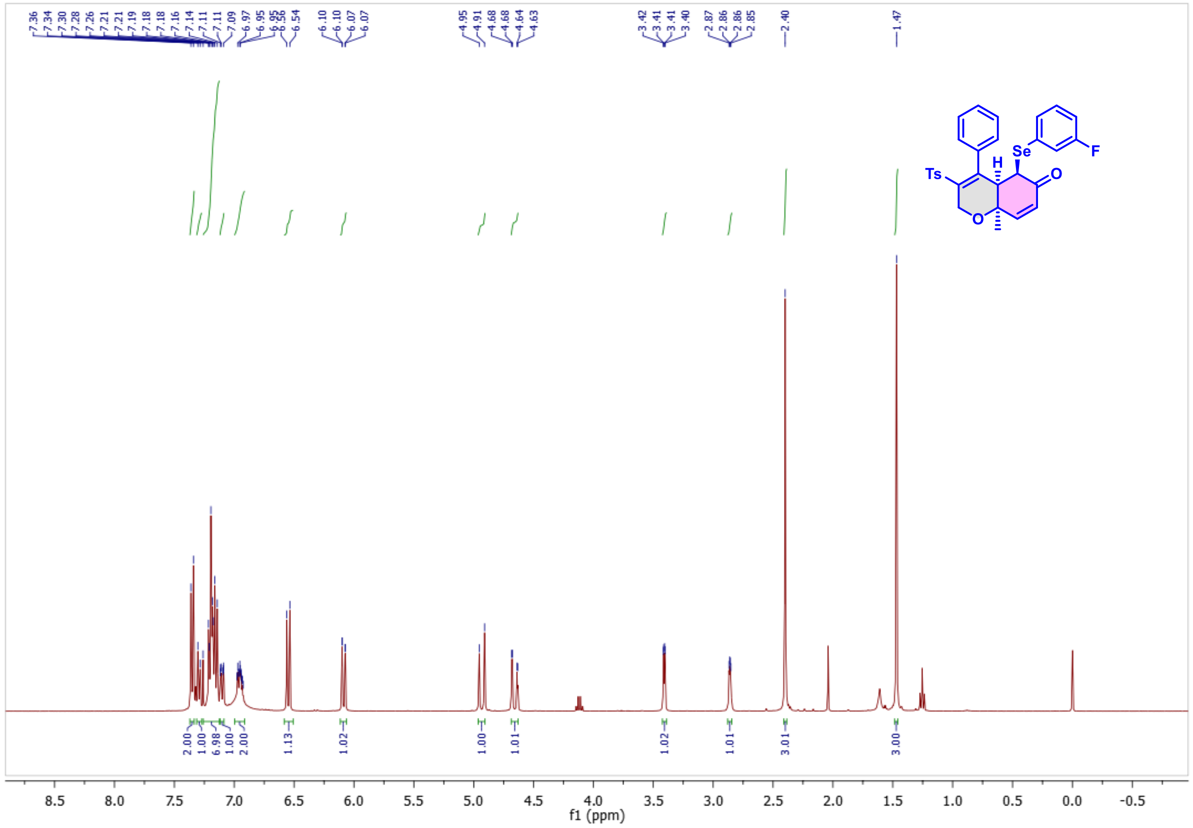

$^{13}C\{^{1}H\}$ NMR (100 MHz, $CDCl_3$) of 4m

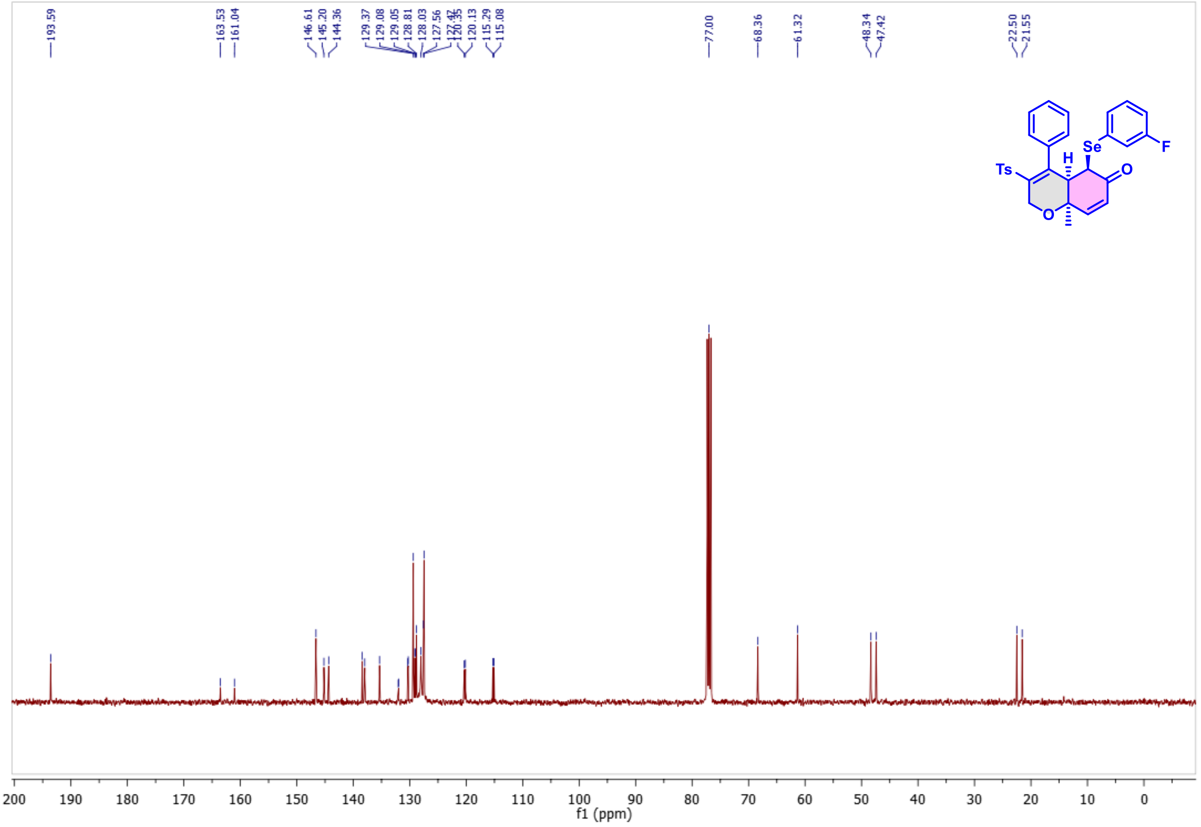

^{1}H NMR (500 MHz, $CDCl_3$) of 4n

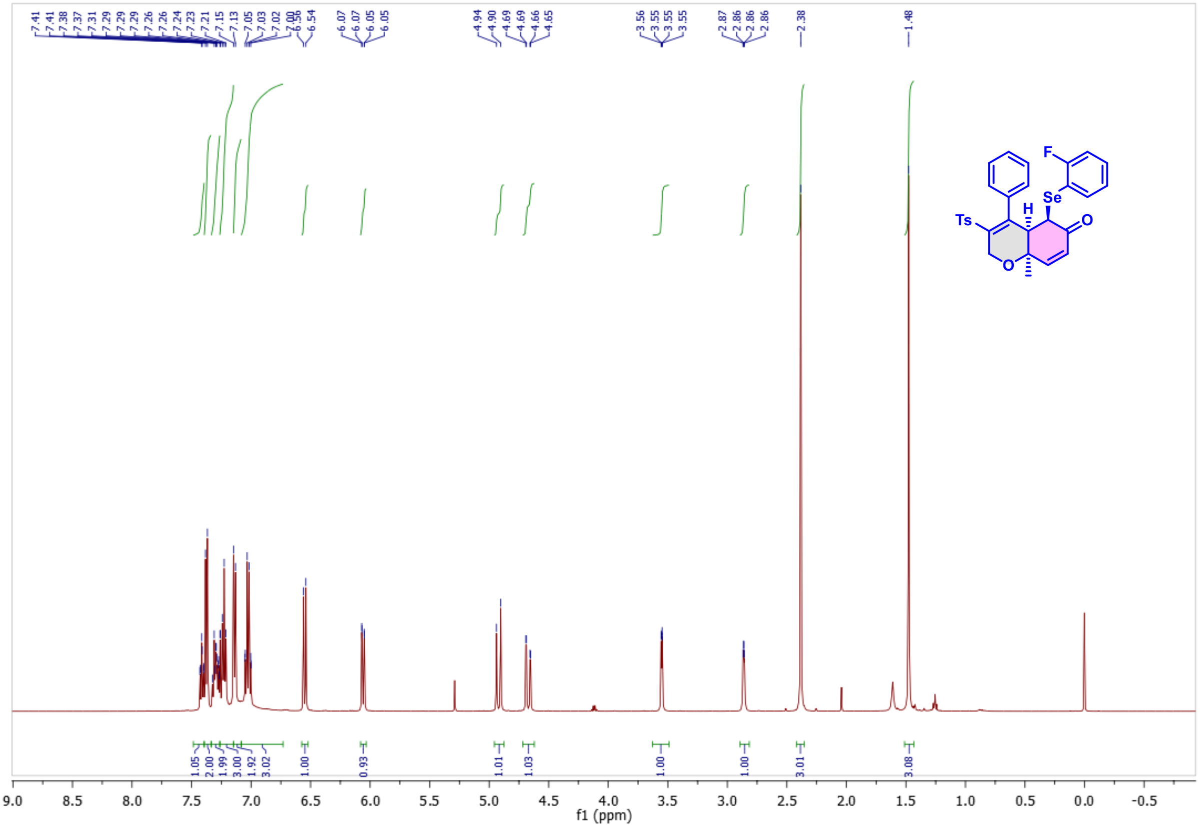

$^{13}C\{^{1}H\}$ NMR (100 MHz, $CDCl_3$) of 4n

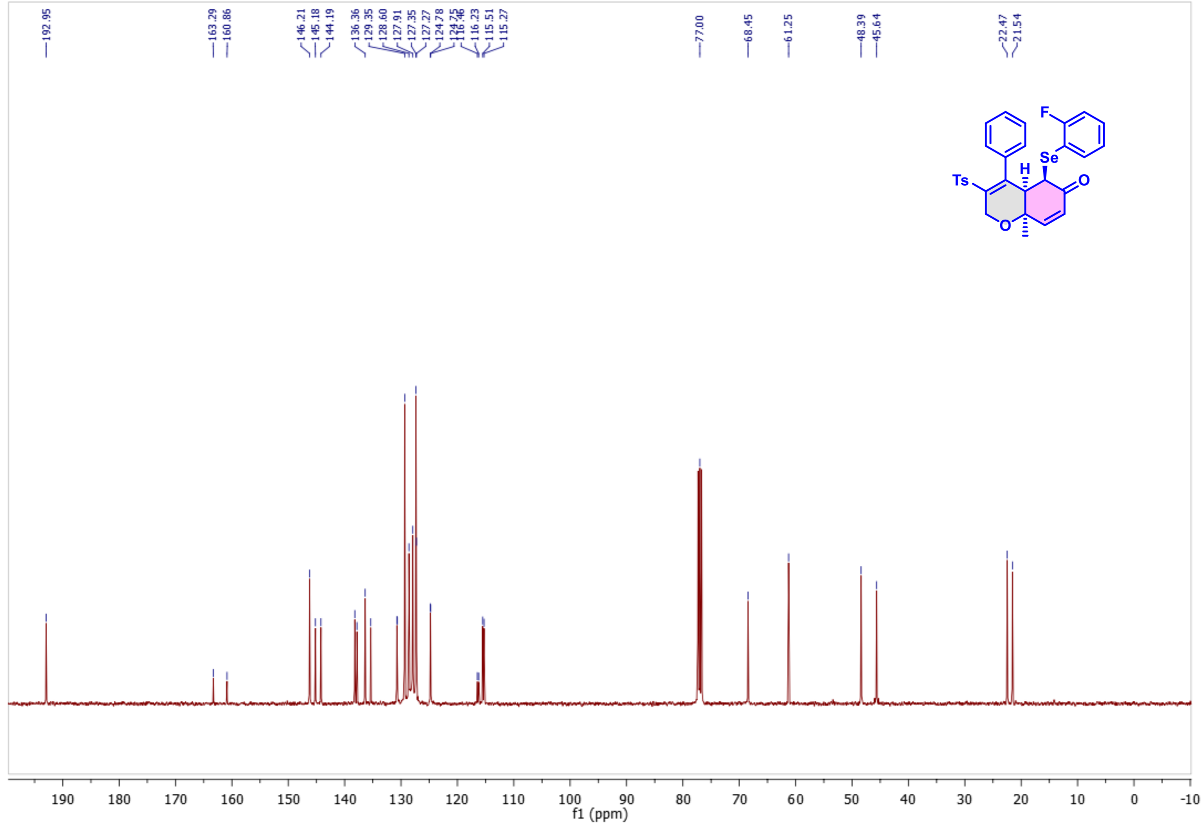

^{1}H NMR (400 MHz, $CDCl_3$) of 4o

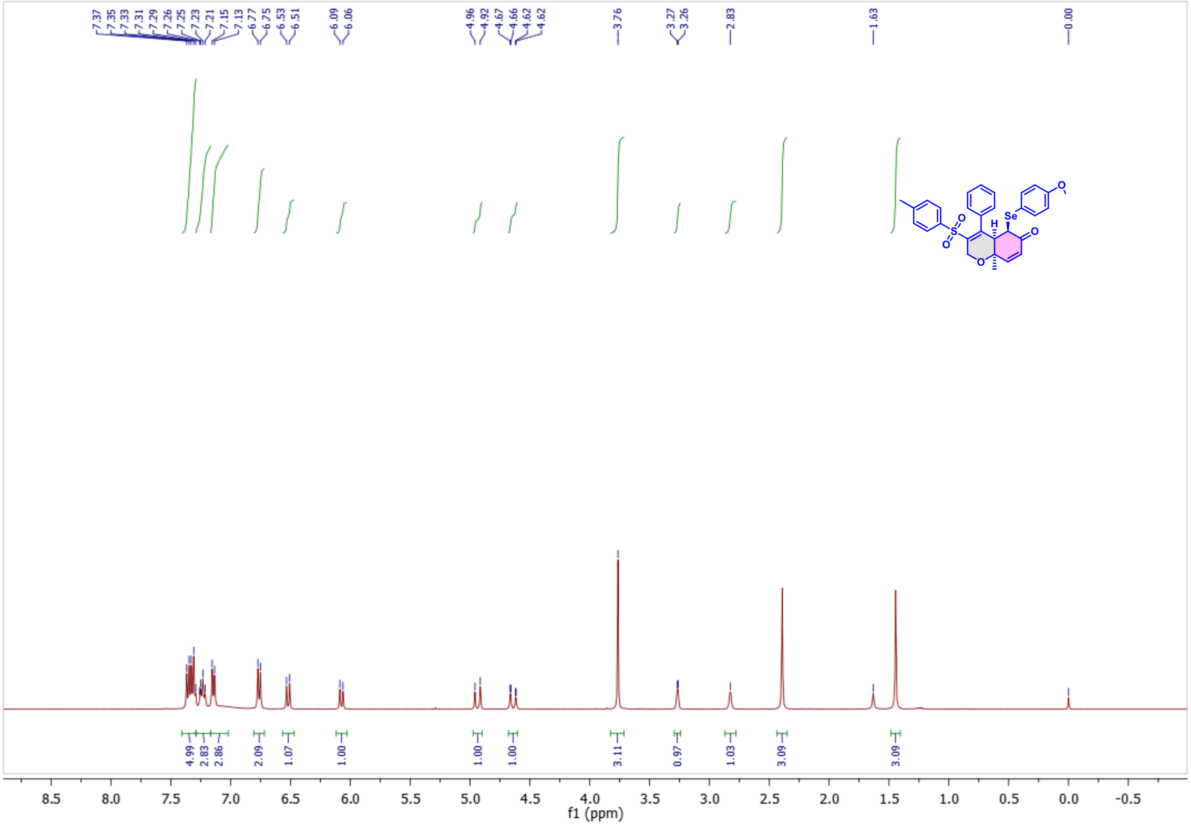

^{13}C{^{1}H} NMR (100 MHz, $CDCl_3$) of 4o

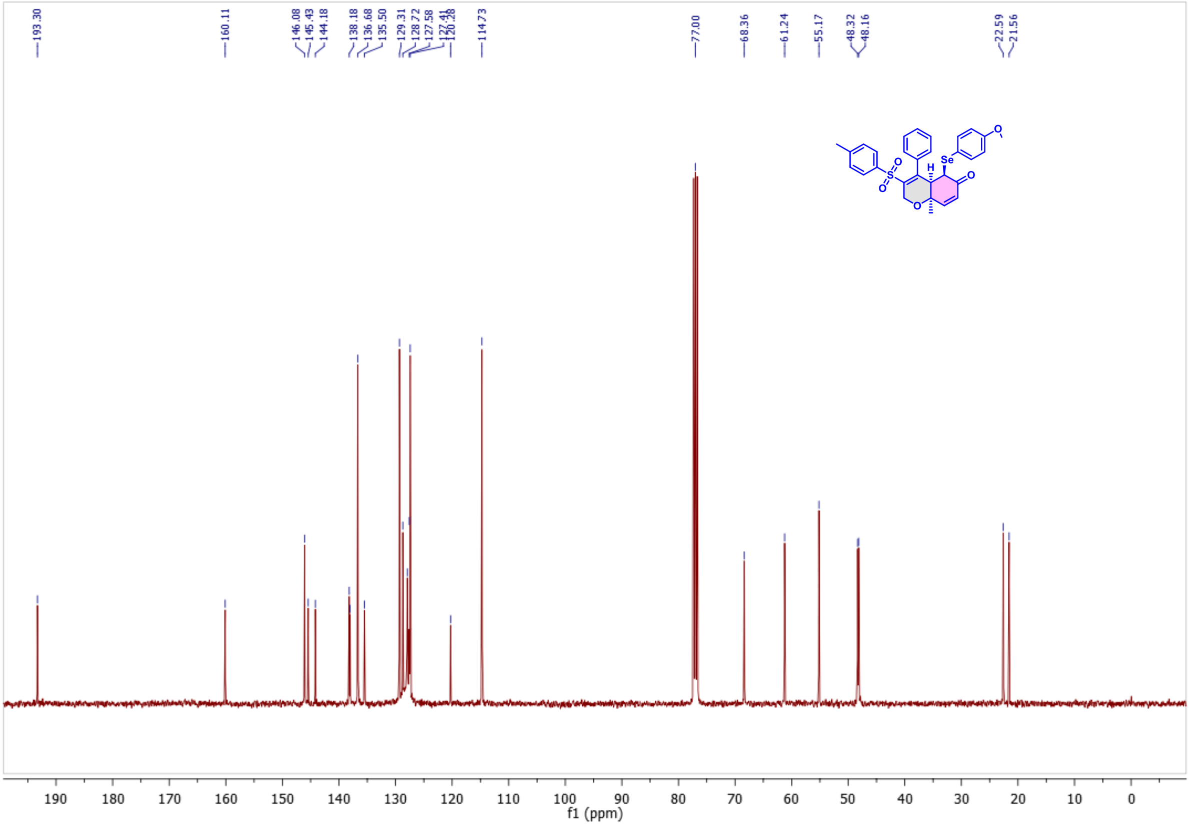

^{1}H NMR (400 MHz, $CDCl_3$) of 4p

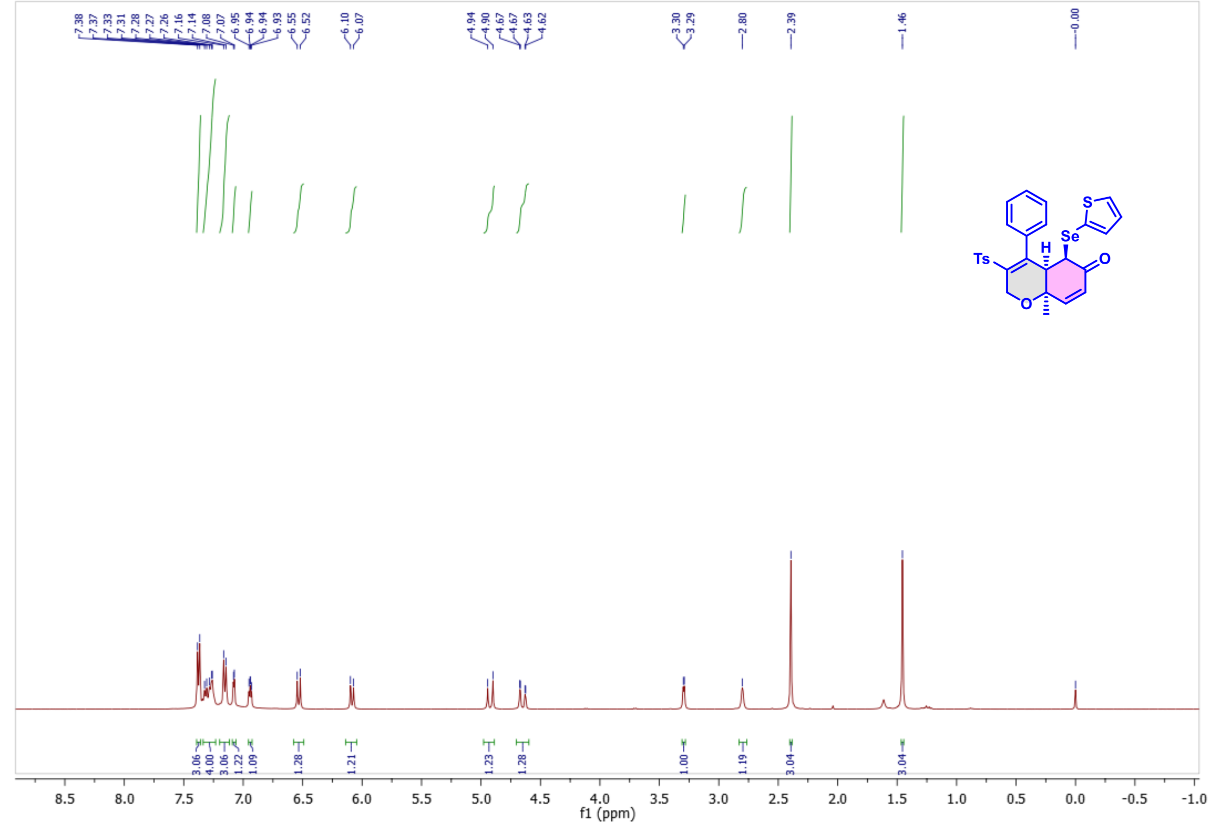

$^{13}C\{^{1}H\}$ NMR (100MHz, $CDCl_3$) of 4p

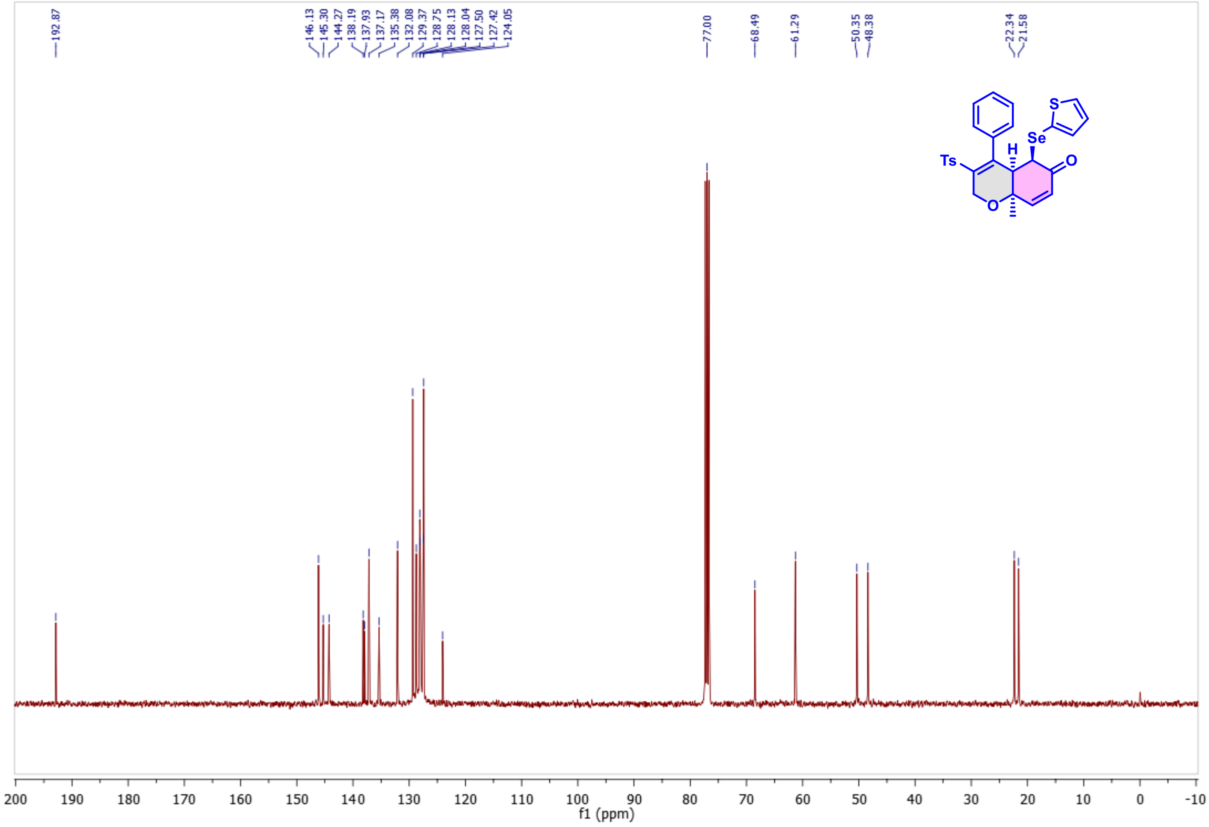

^{1}H NMR (400 MHz, $CDCl_3$) of 4q

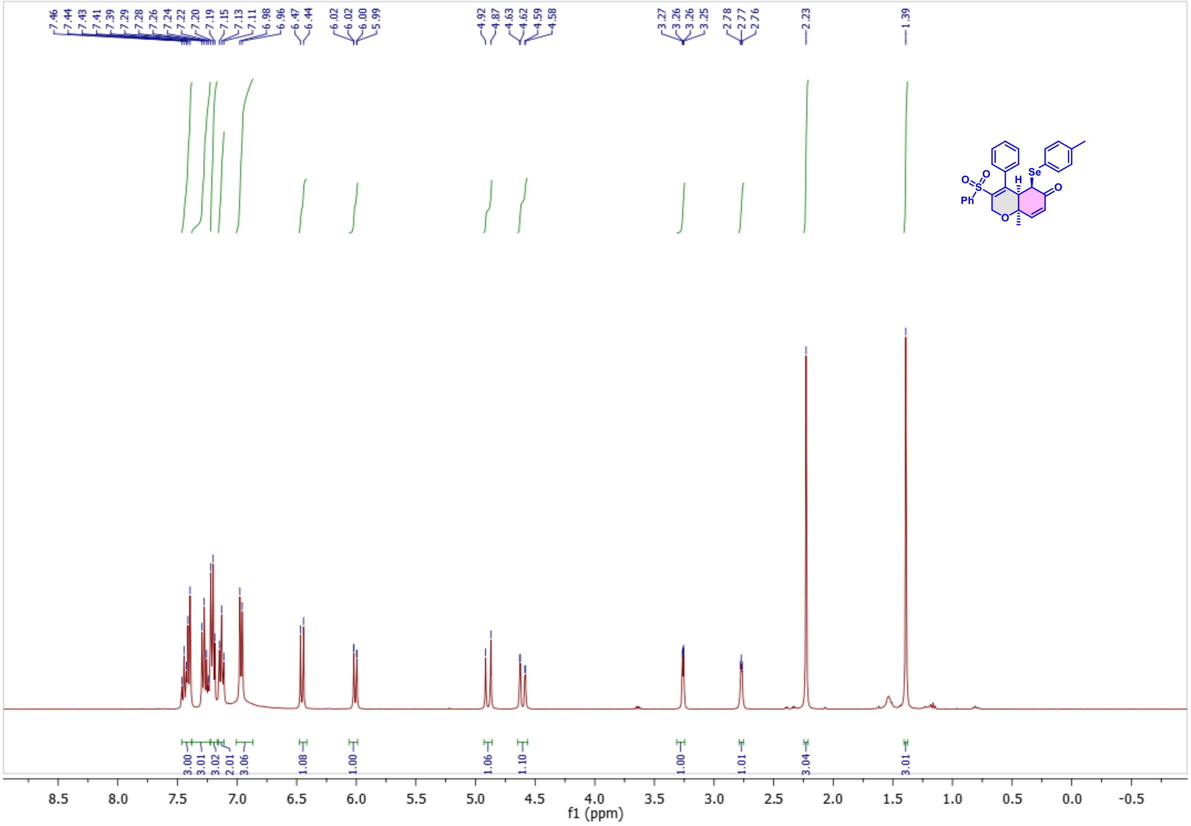

$^{13}C\{^{1}H\}$ NMR (125 MHz, $CDCl_3$) of 4q

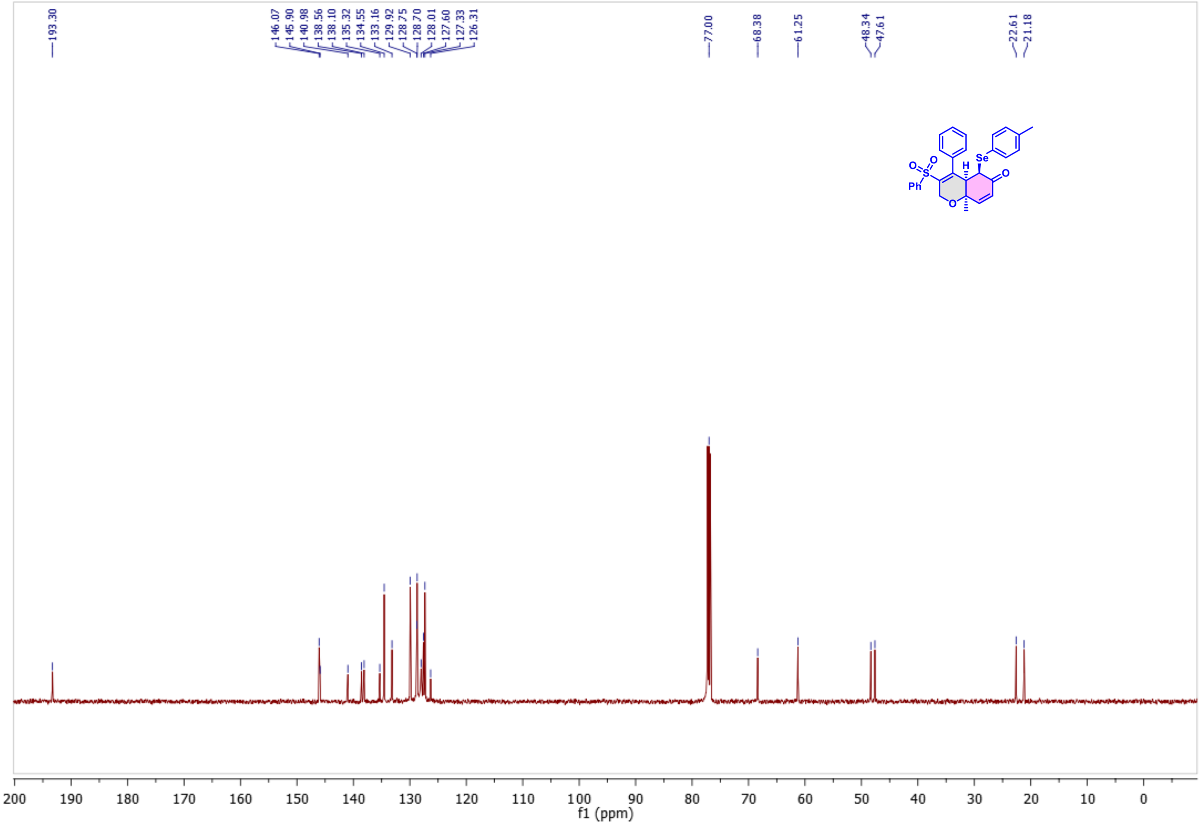

^{1}H NMR (500 MHz, $CDCl_3$) of 4r

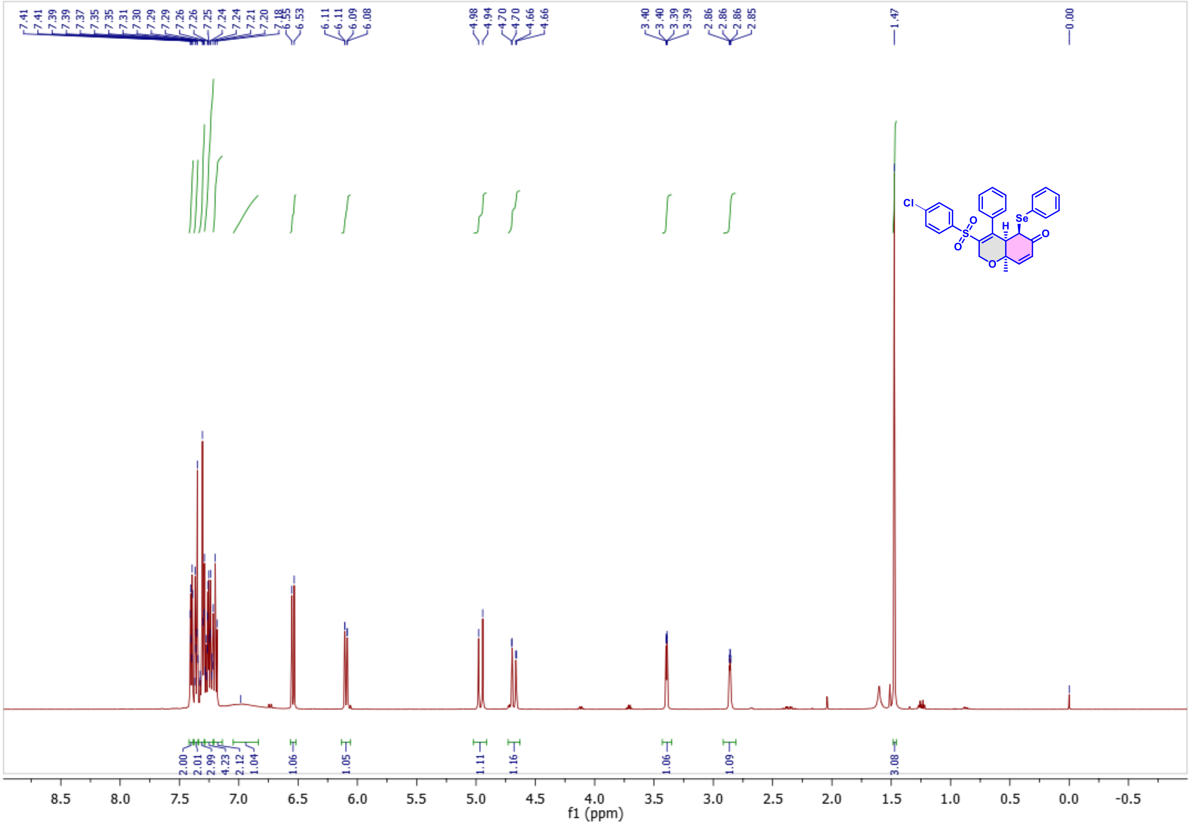

$^{13}C\{^{1}H\}$ NMR (100 MHz, $CDCl_3$) of 4r

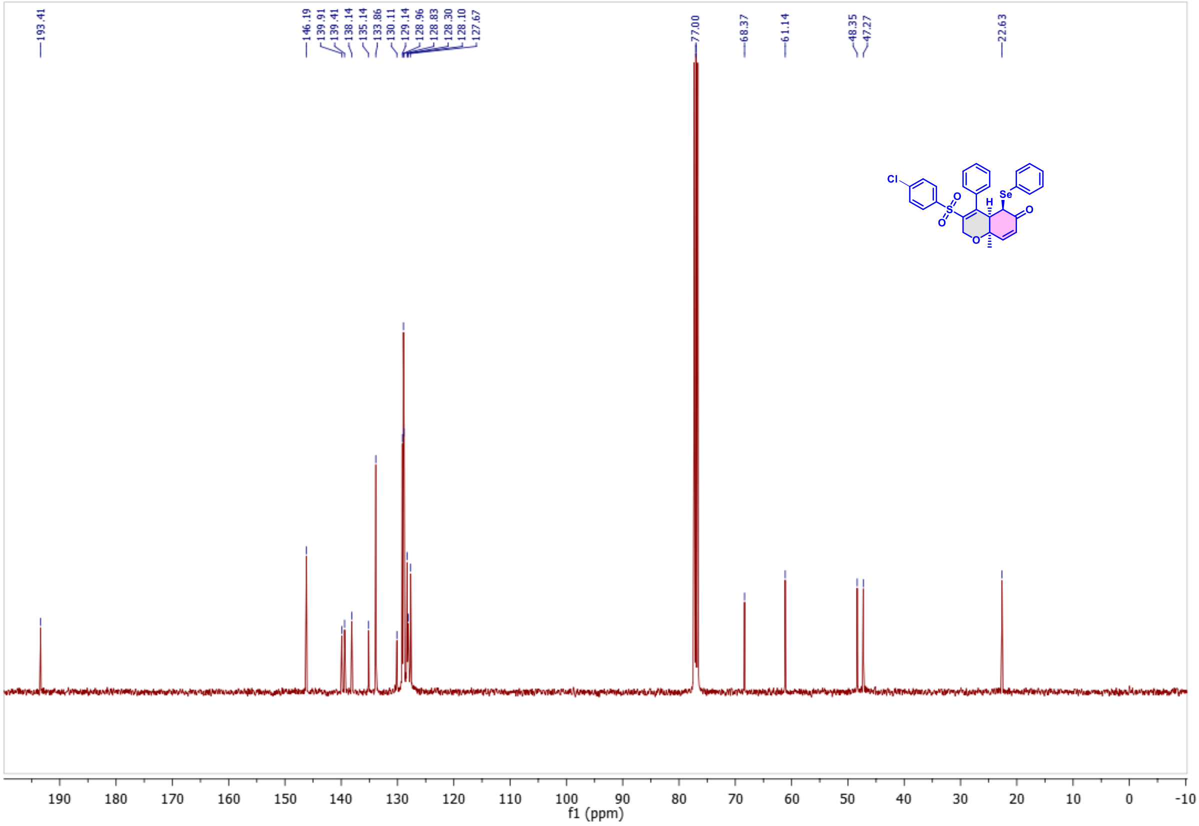

^{1}H NMR (500 MHz, $CDCl_3$) of 4s

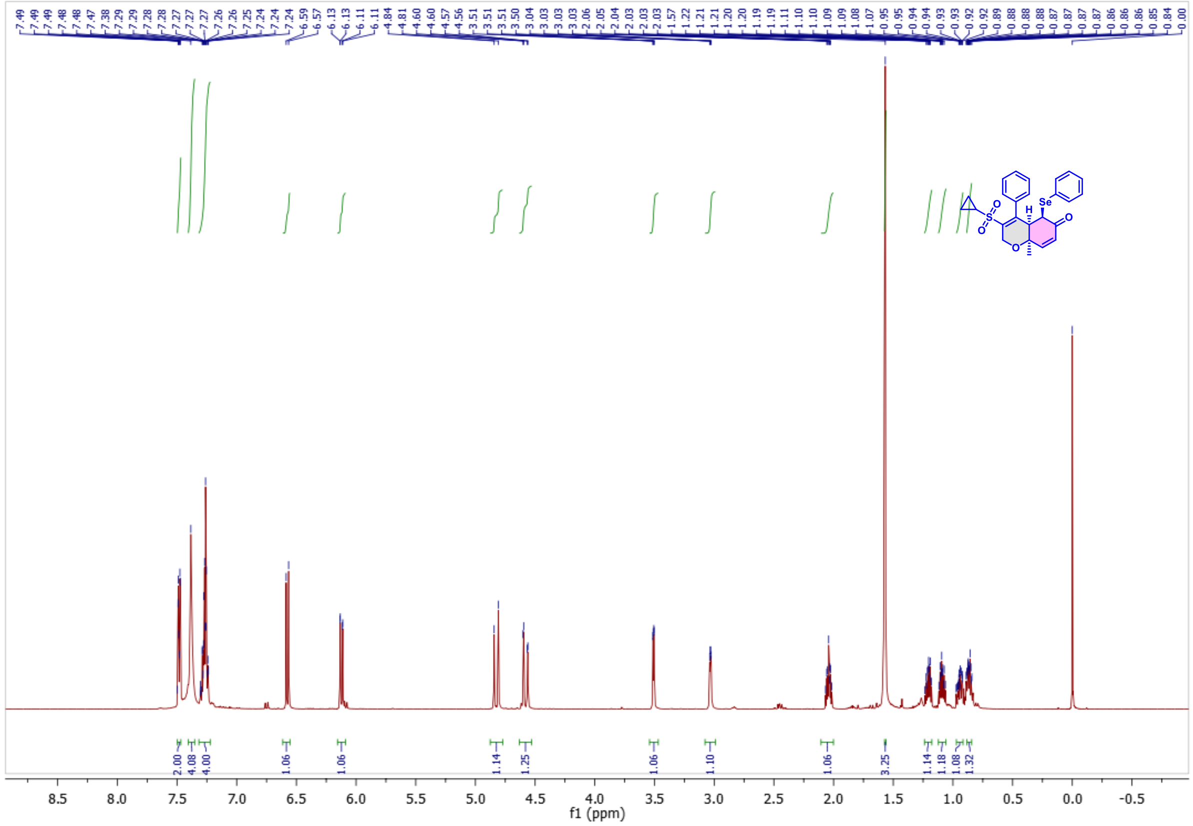

^{13}C{^{1}H} NMR (100 MHz, $CDCl_3$) of 4s

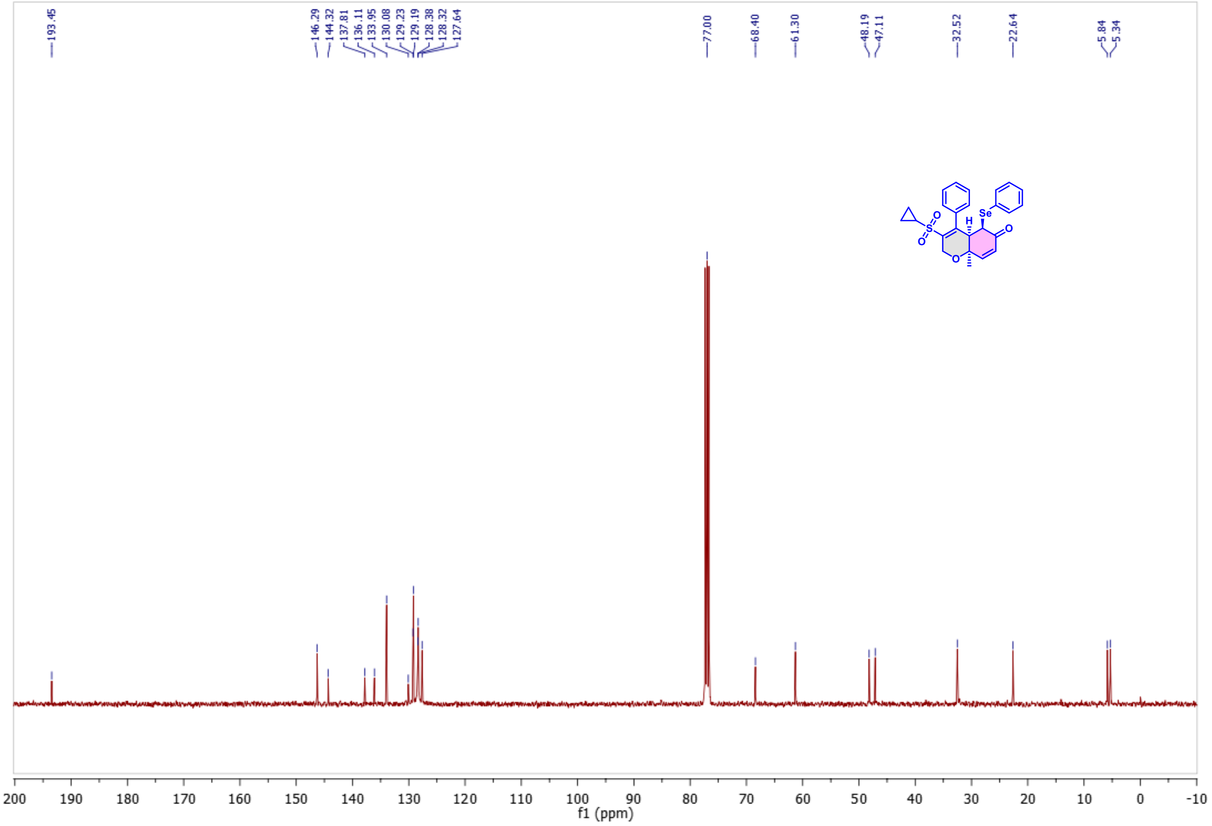

^{1}H NMR (500 MHz, $CDCl_3$) of 4t

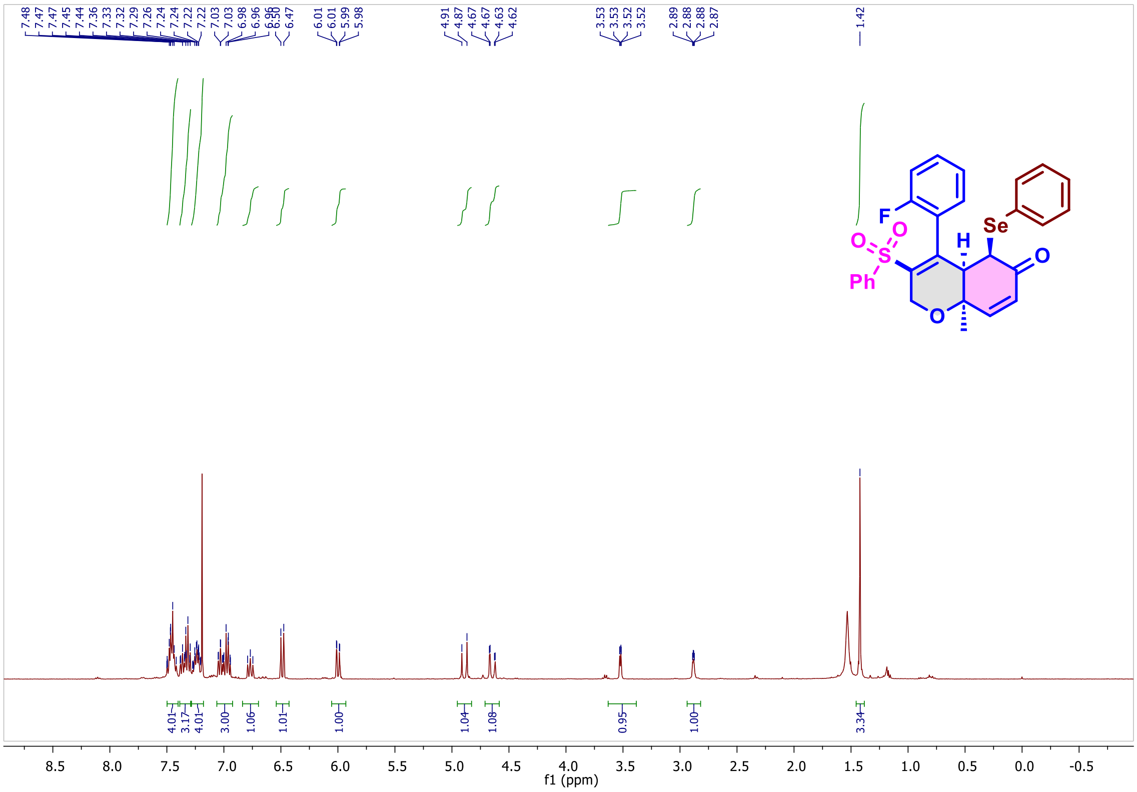

$^{13}C\{^{1}H\}$ NMR (100 MHz, $CDCl_3$) of 4t

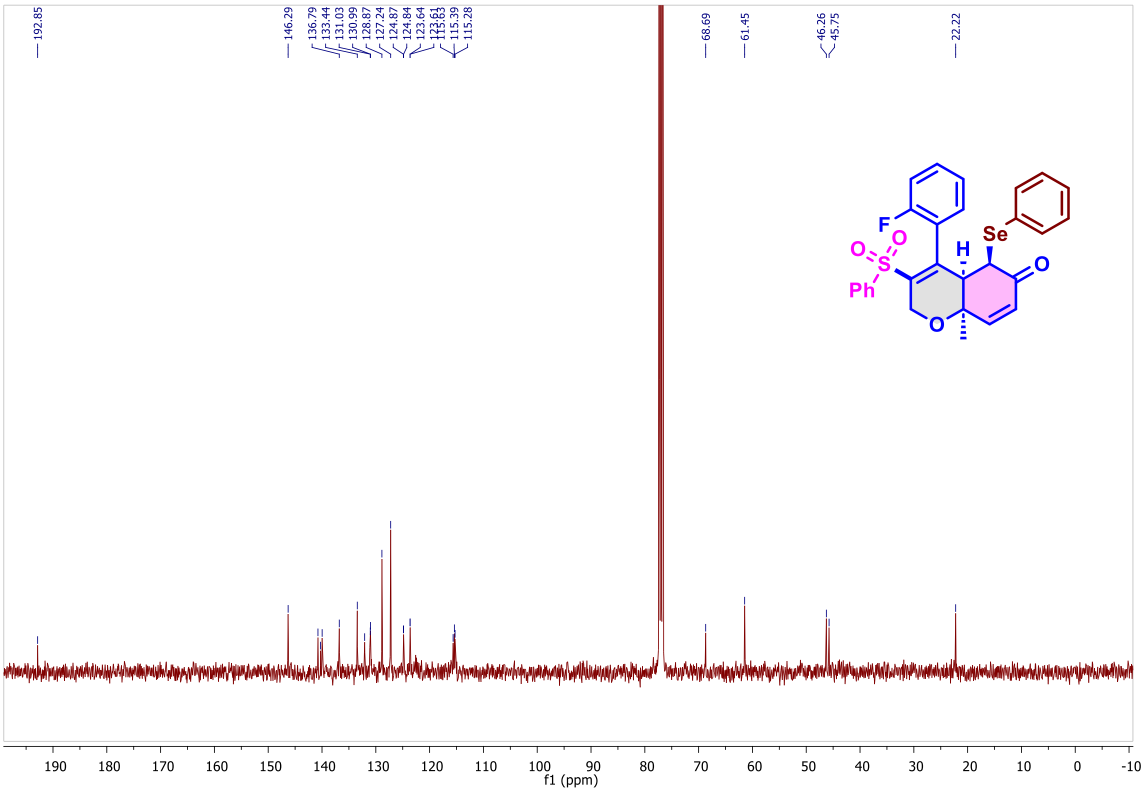

^{1}H NMR (400 MHz, $CDCl_3$) of 6a

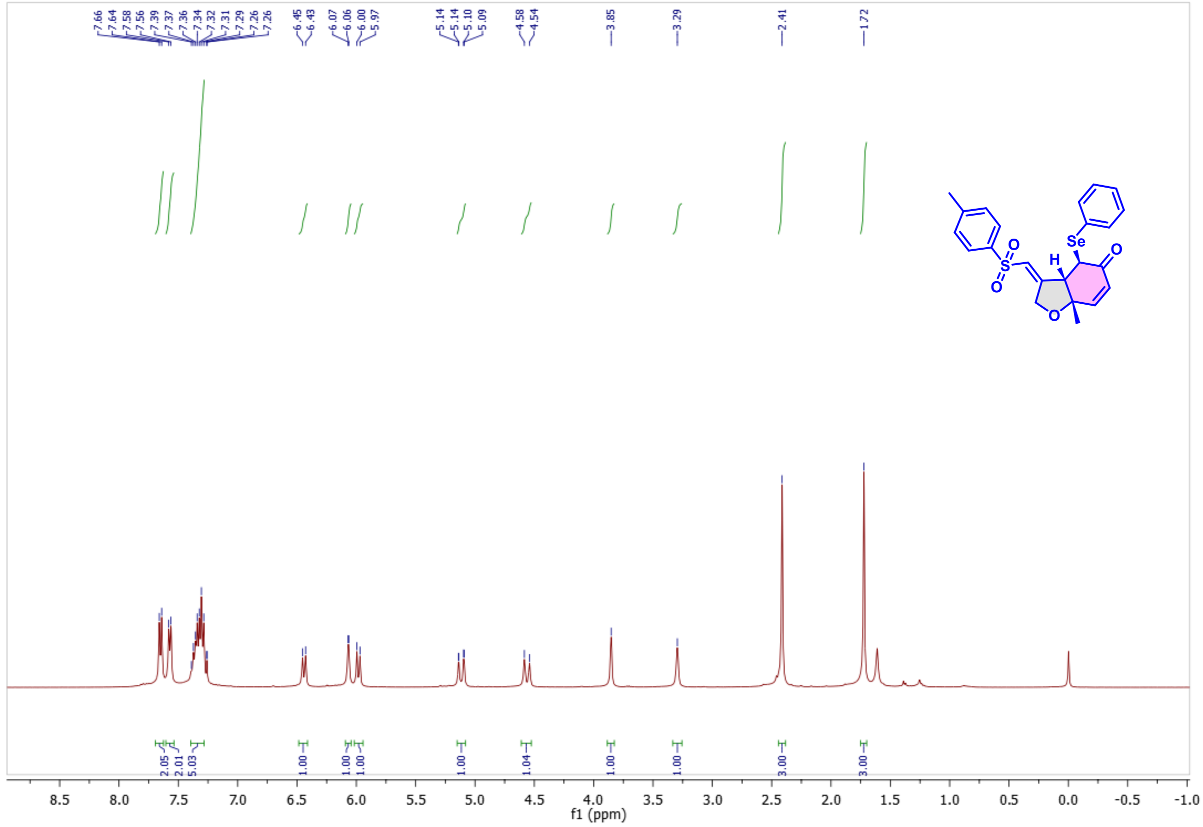

^{13}C{^{1}H} NMR (100 MHz, $CDCl_3$) of 6a

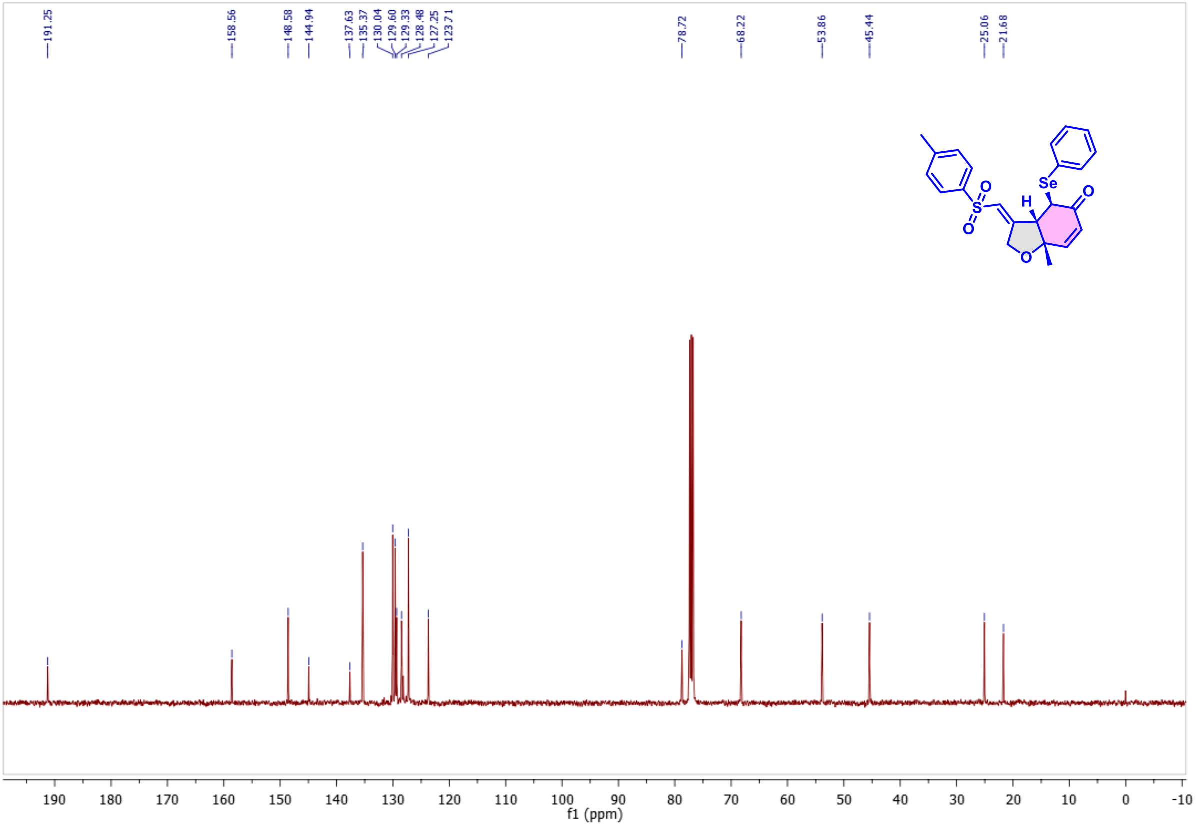

^{1}H NMR (500 MHz, $CDCl_3$) of 6b

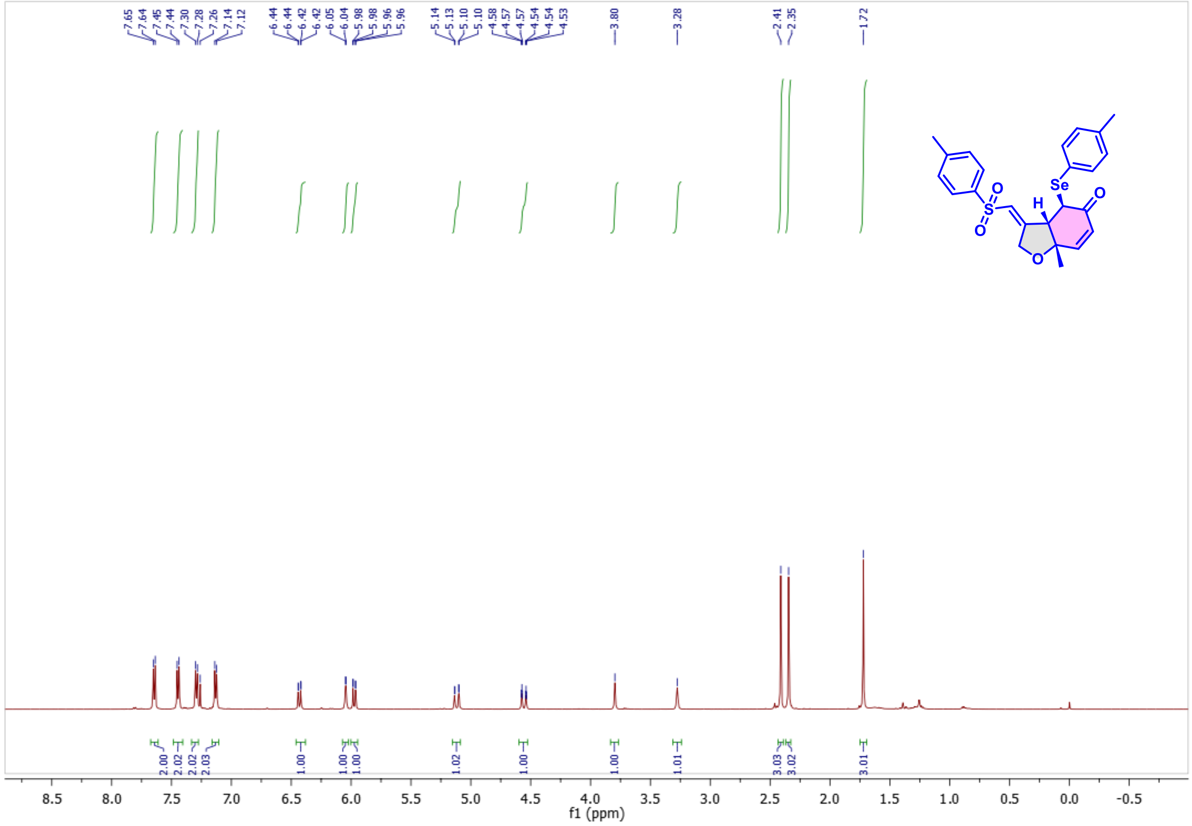

$^{13}C\{^{1}H\}$ NMR (100 MHz, $CDCl_3$) of 6b

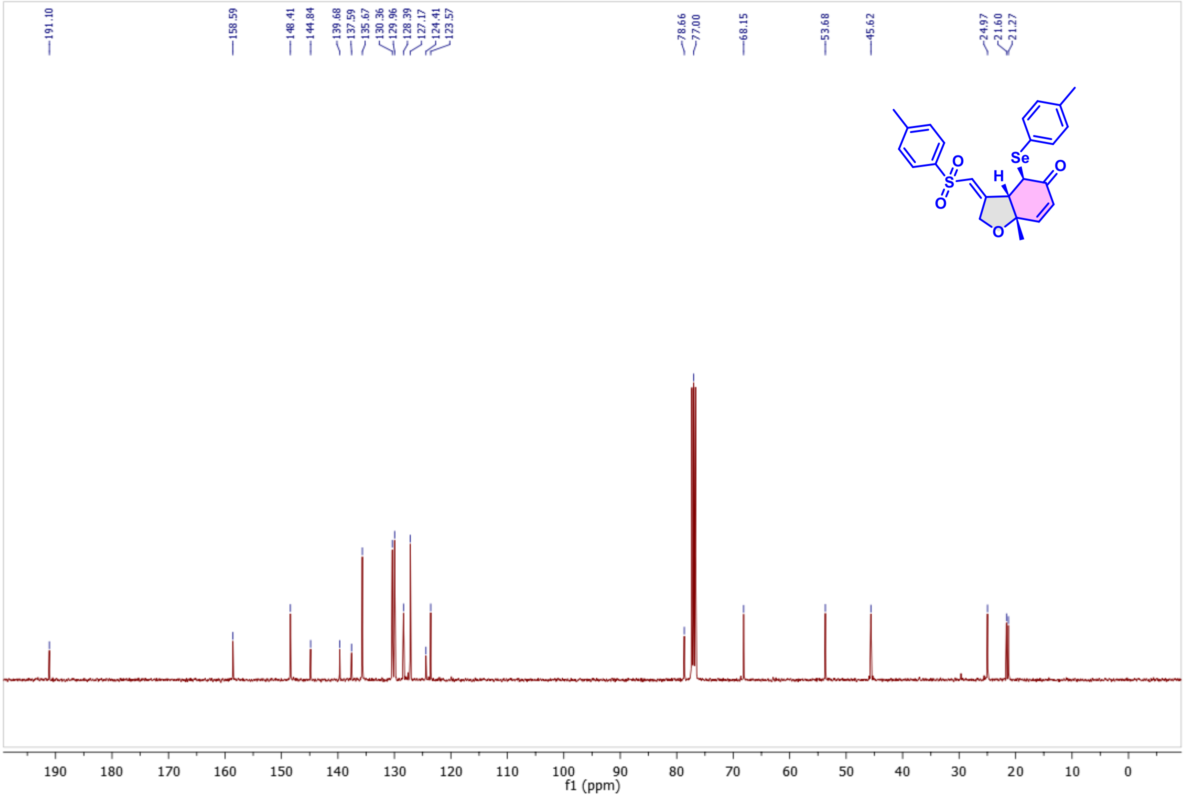

^{1}H NMR (500 MHz, $CDCl_3$) of 6c

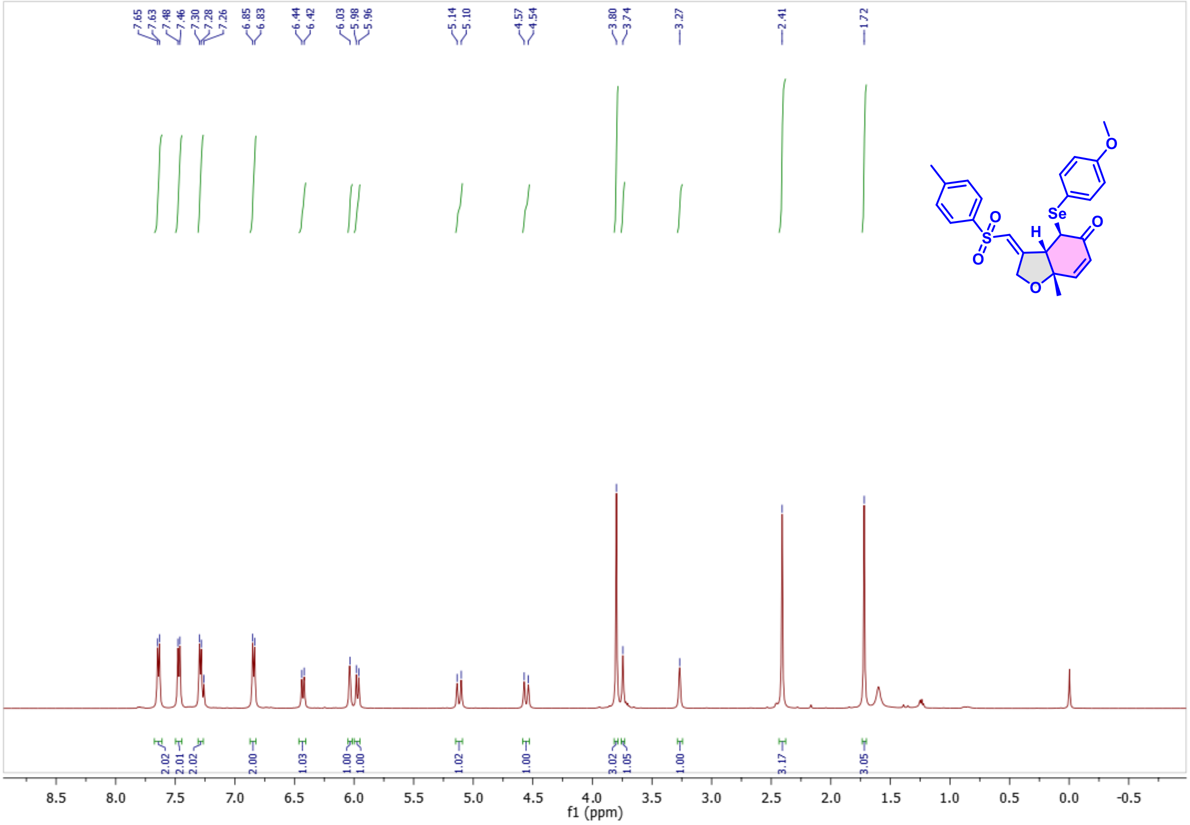

^{13}C{^{1}H} NMR (100 MHz, $CDCl_3$) of 6c

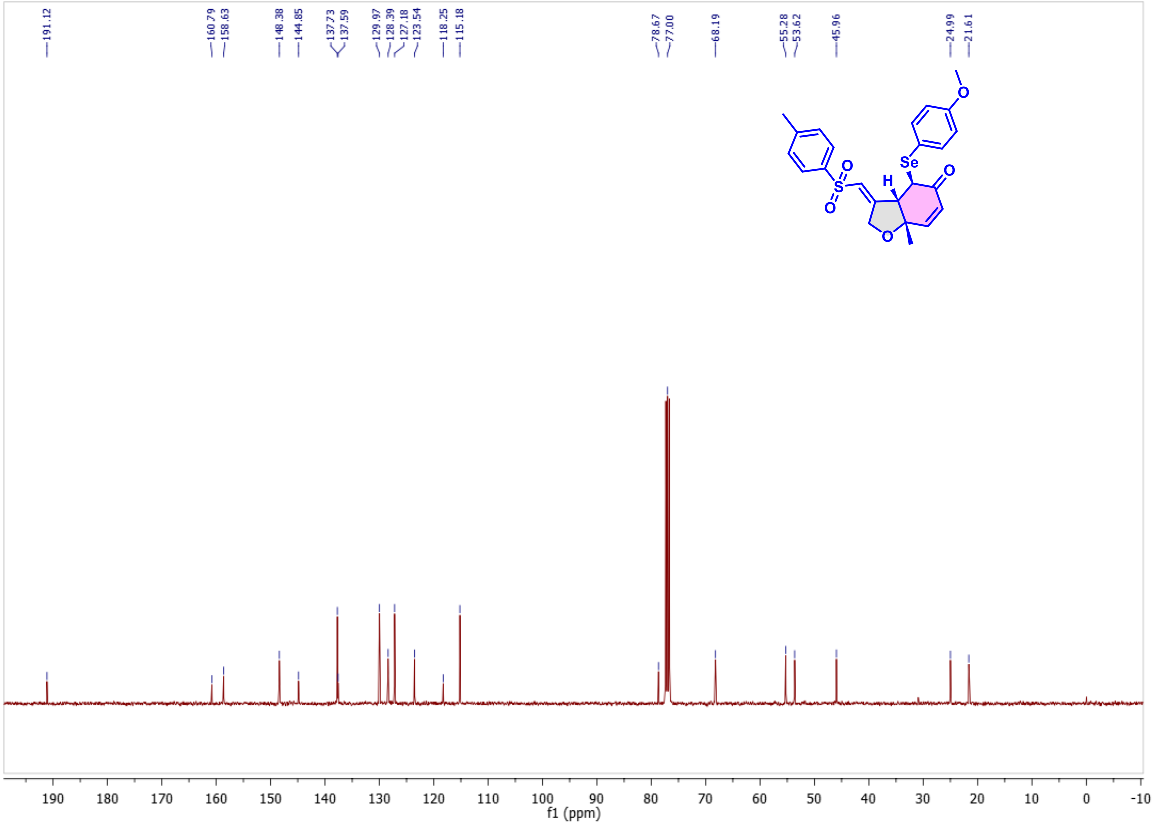

^{1}H NMR (500 MHz, CDCl₃) of 6d

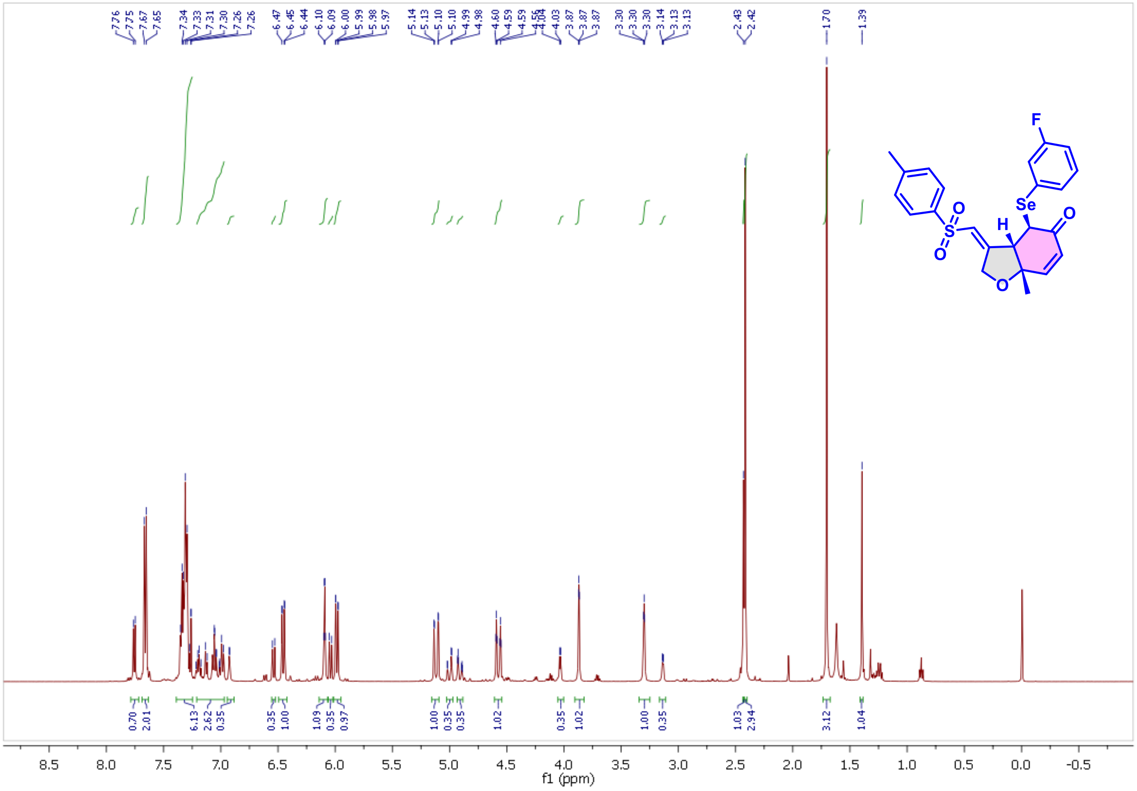

^{13}C{^{1}H} NMR (100 MHz, CDCl₃) of 6d

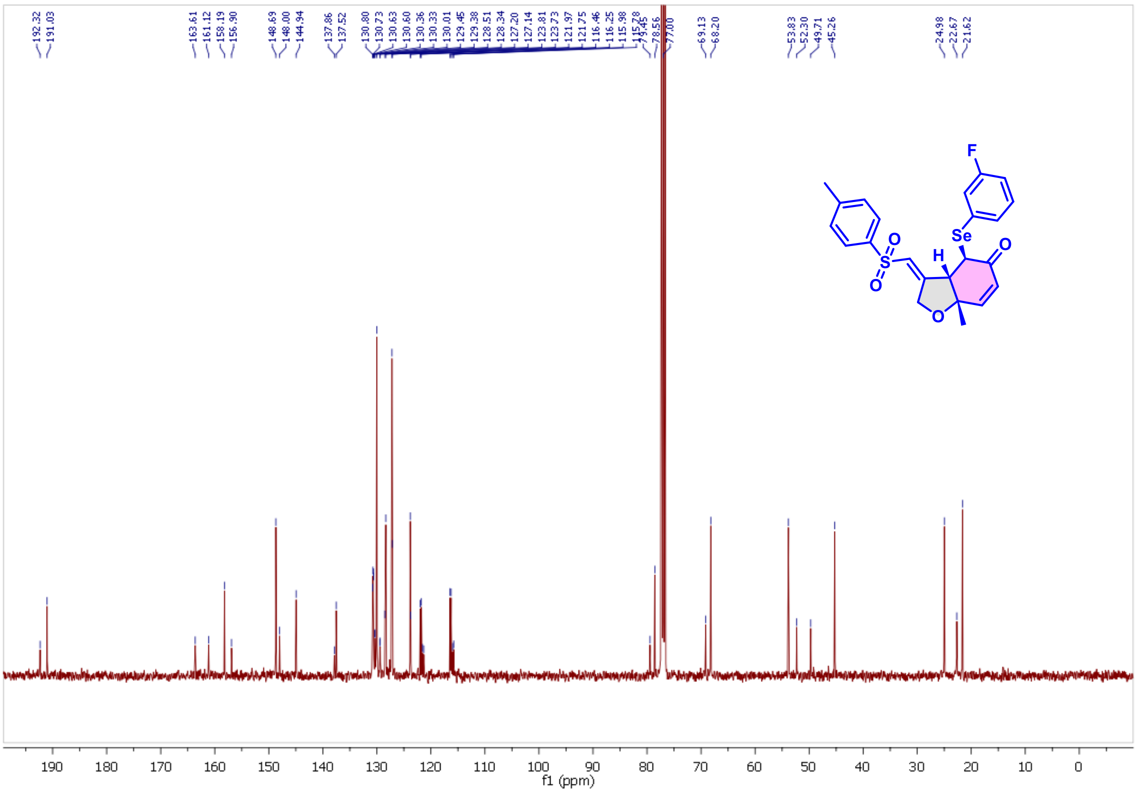

^{1}H NMR (400 MHz, $CDCl_3$) of 6e

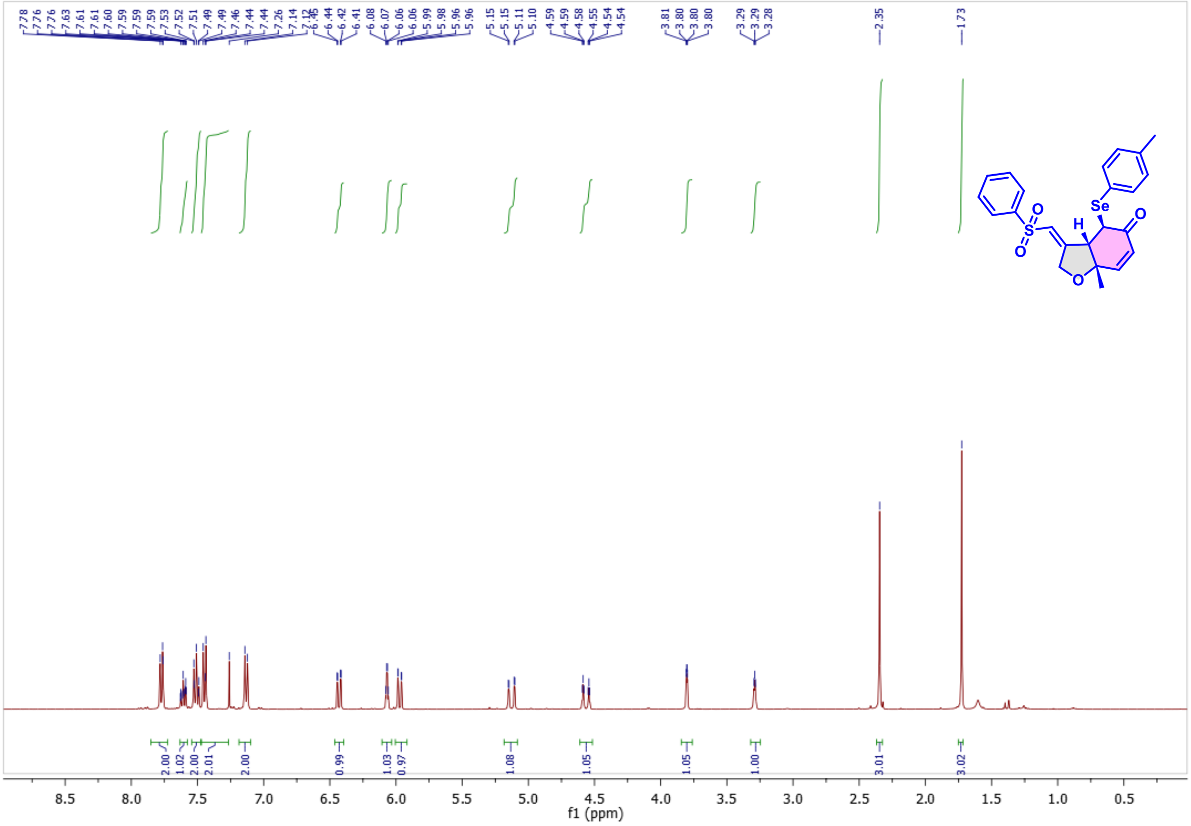

$^{13}C\{^{1}H\}$ NMR (100 MHz, $CDCl_3$) of 6e

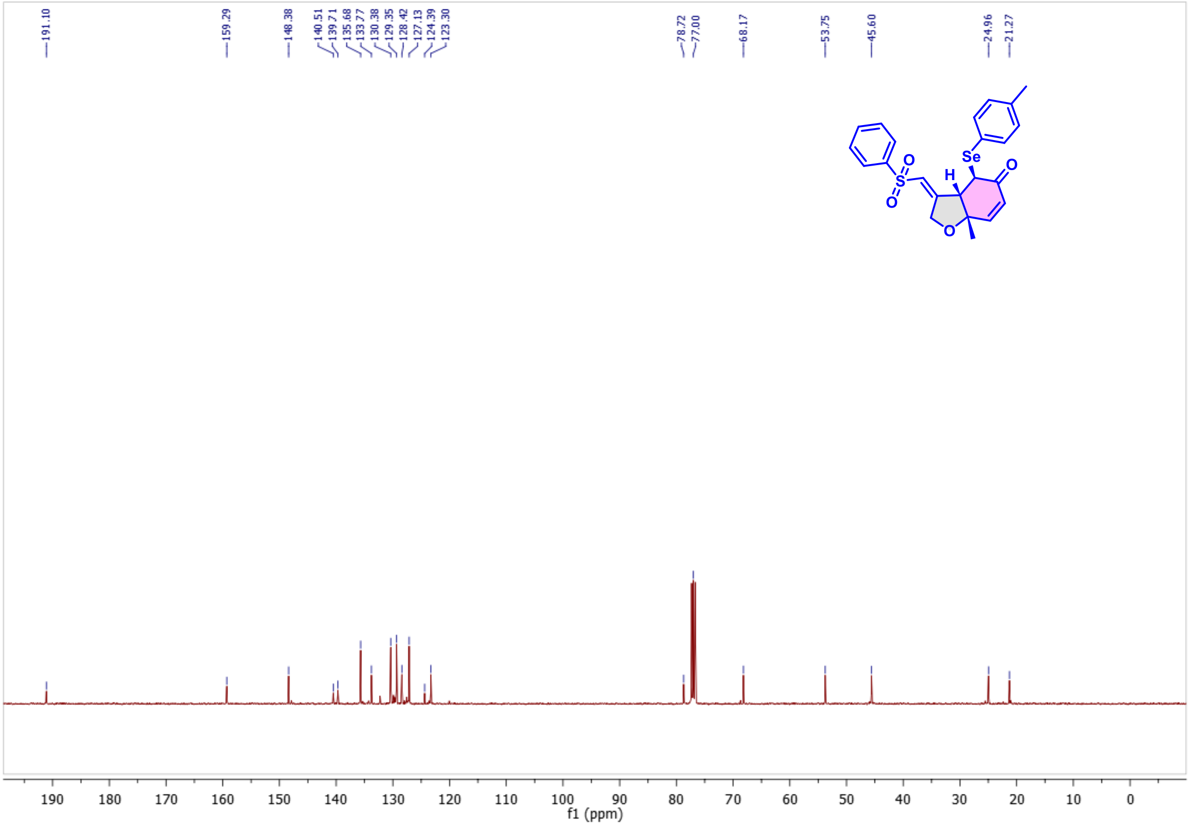

^{1}H NMR (400 MHz, $CDCl_3$) of 6f

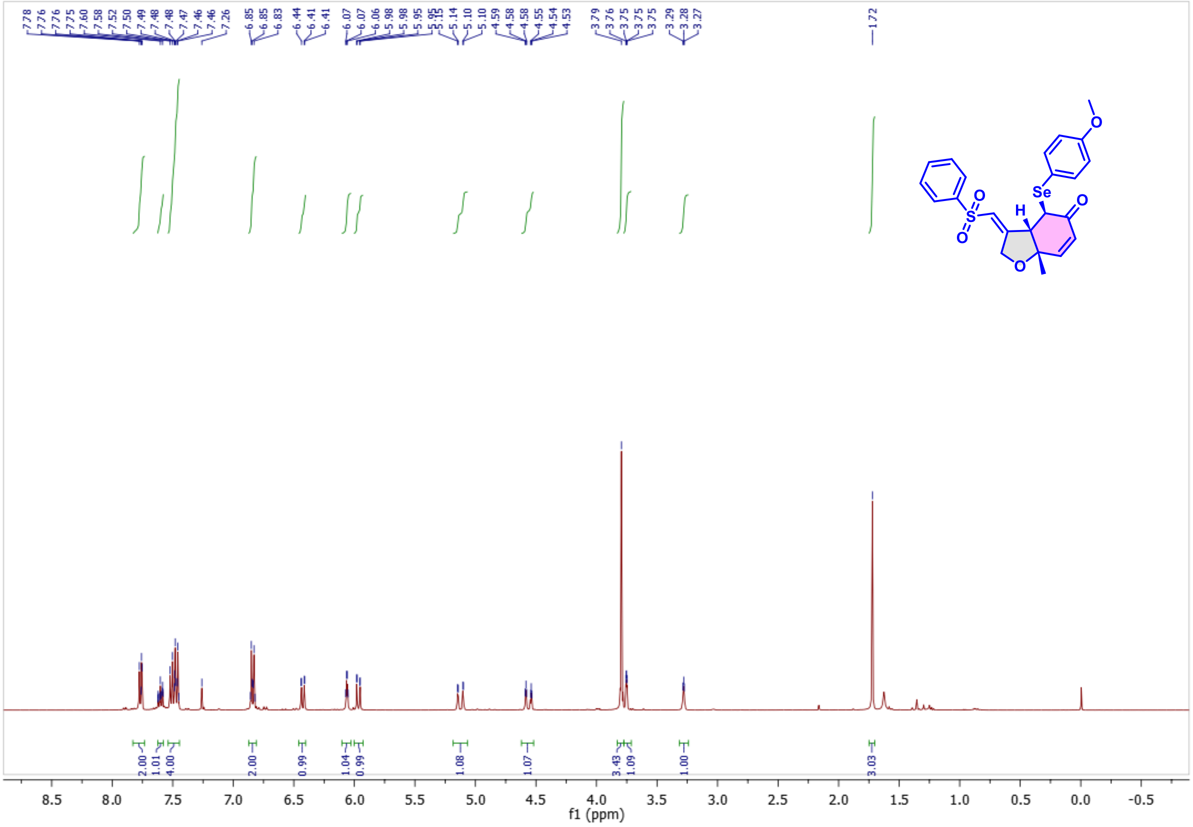

^{13}C{^{1}H} NMR (100 MHz, $CDCl_3$) of 6f

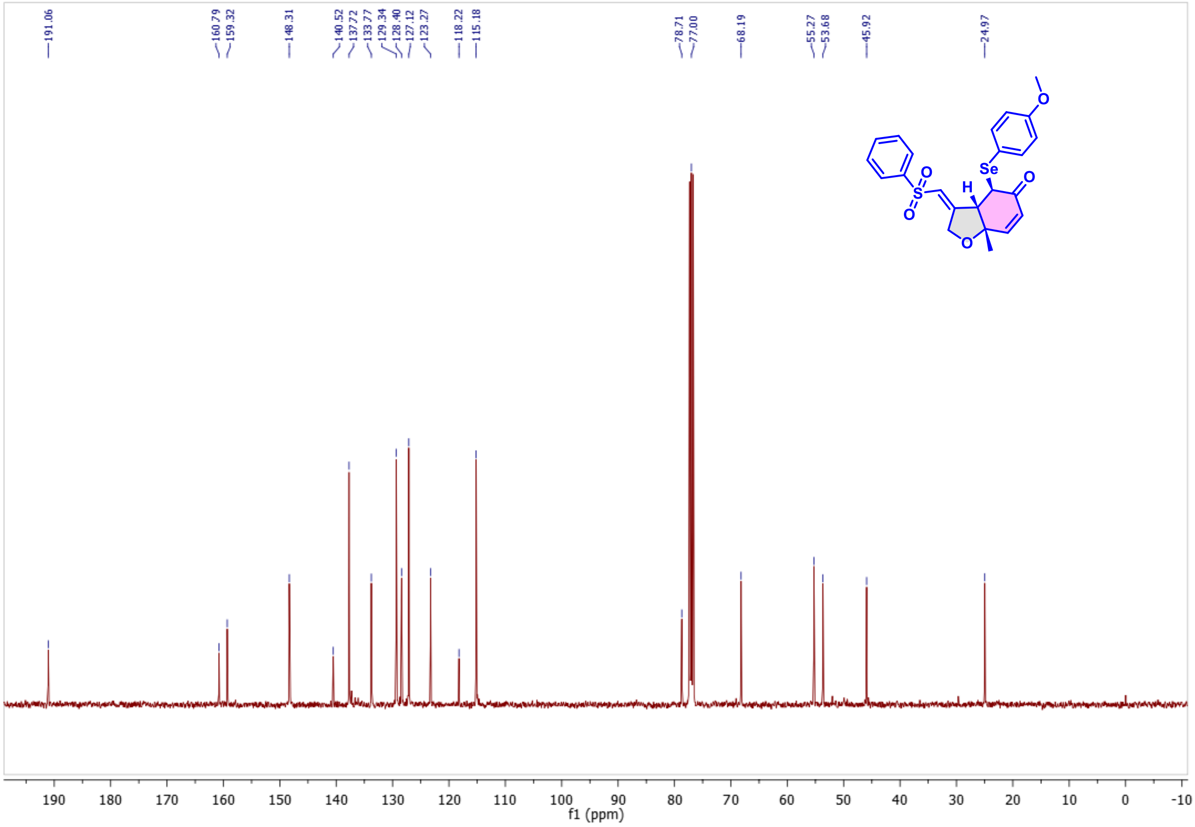

^{1}H NMR (500 MHz, $CDCl_3$) of 6g

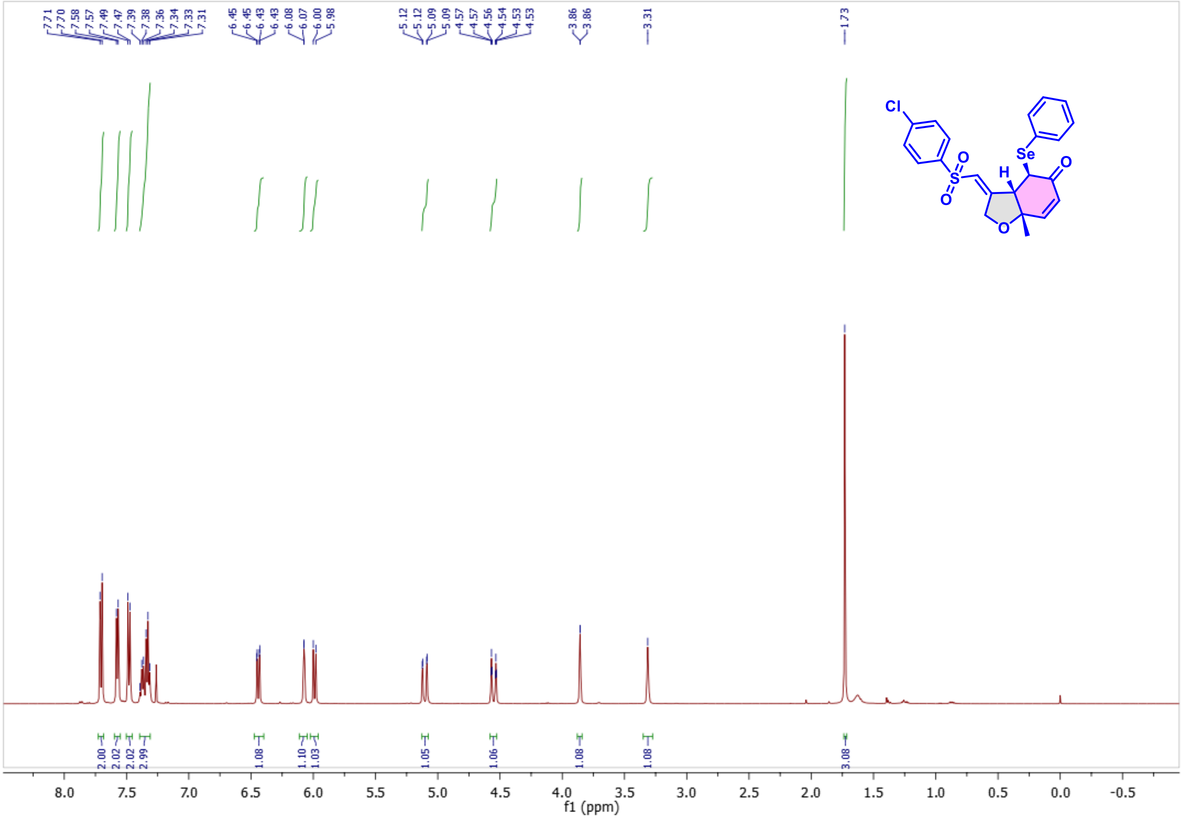

$^{13}C\{^{1}H\}$ NMR (100 MHz, $CDCl_3$) of 6g

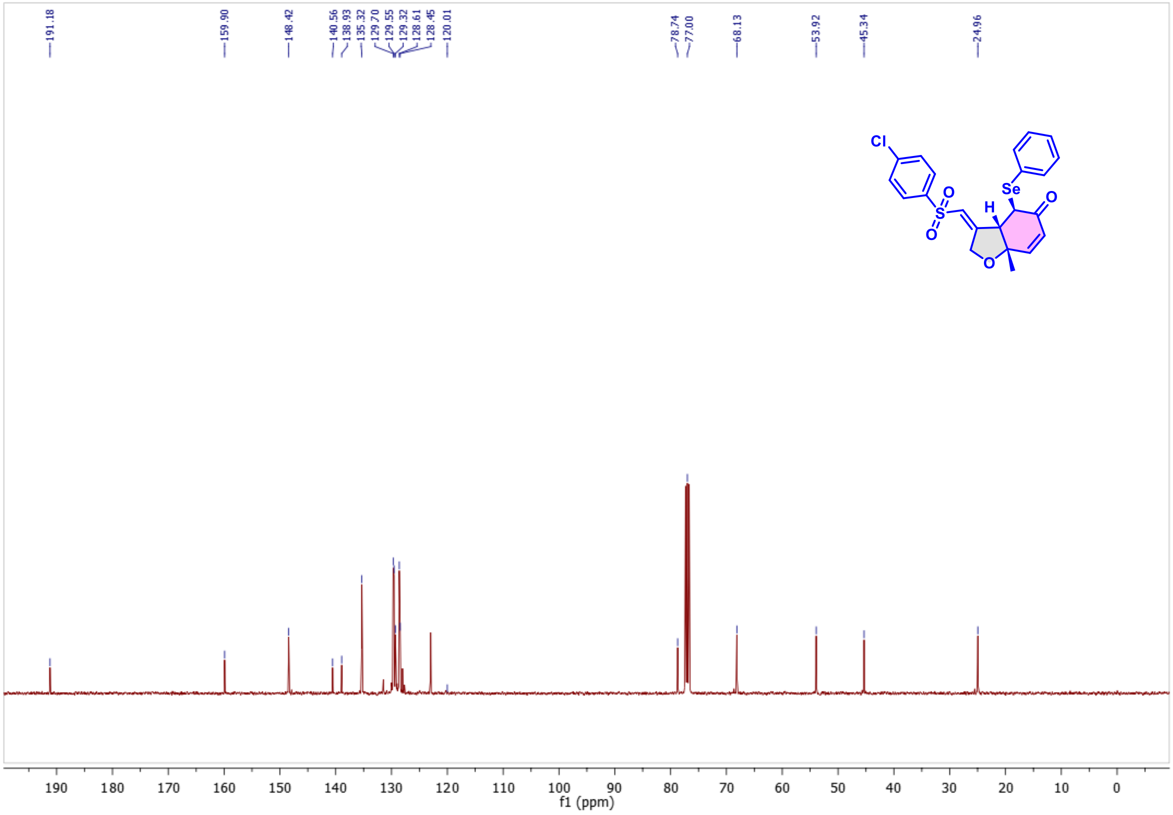

^1H NMR (500 MHz, $CDCl_3$) of 6h

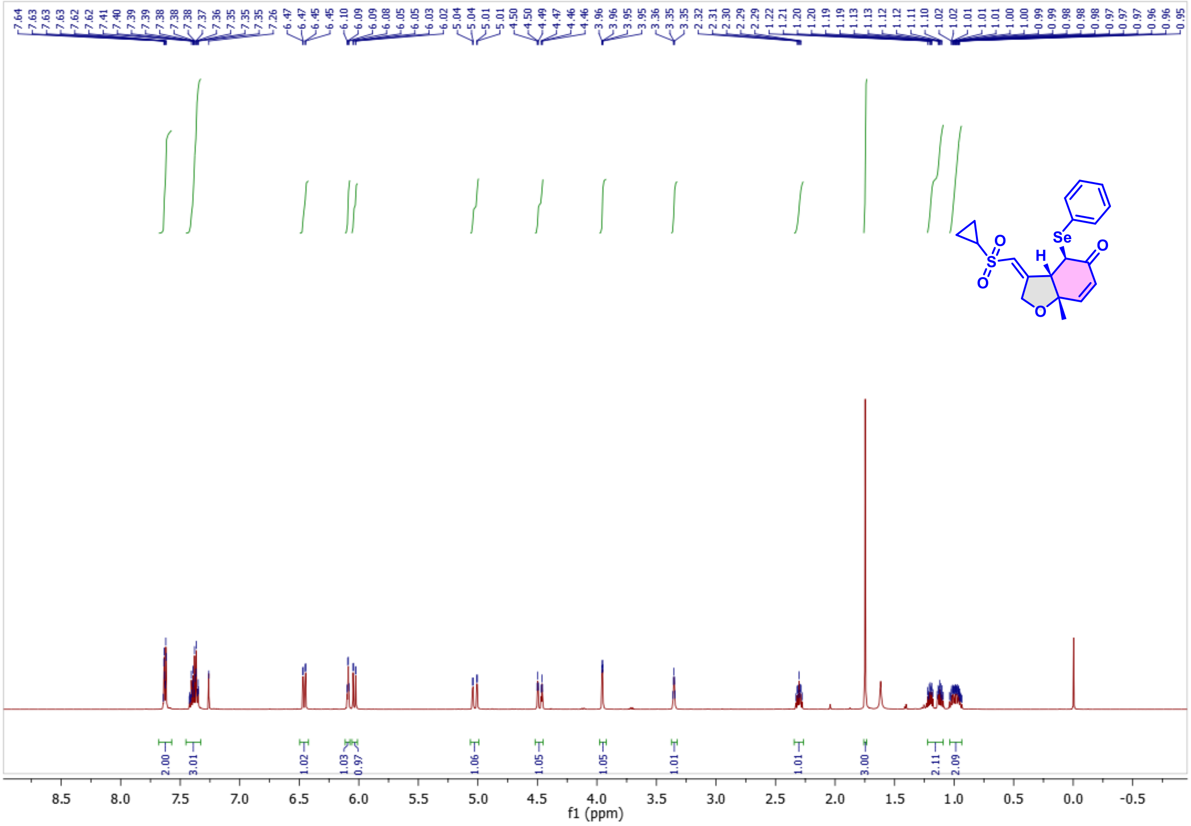

$^{13}C\{^1H\}$ NMR (100 MHz, $CDCl_3$) of 6h

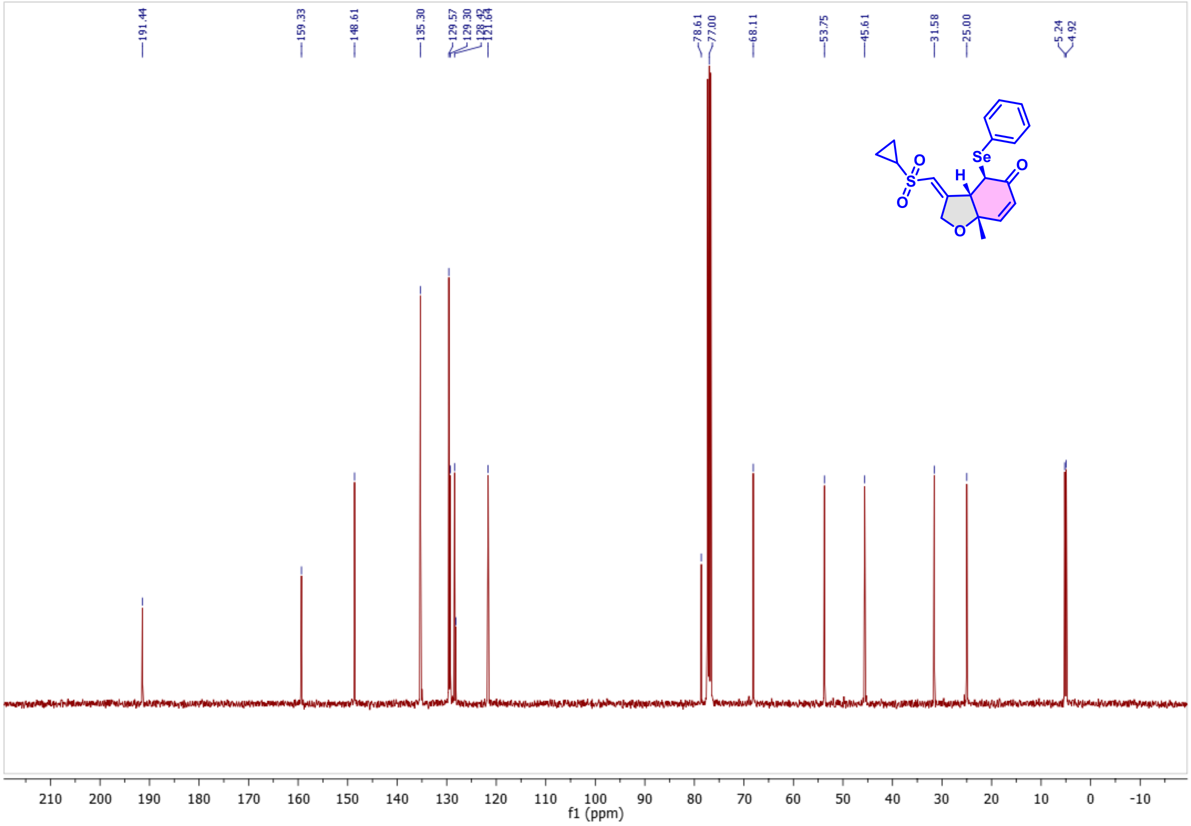

^{1}H NMR (400 MHz, $CDCl_3$) of 6i

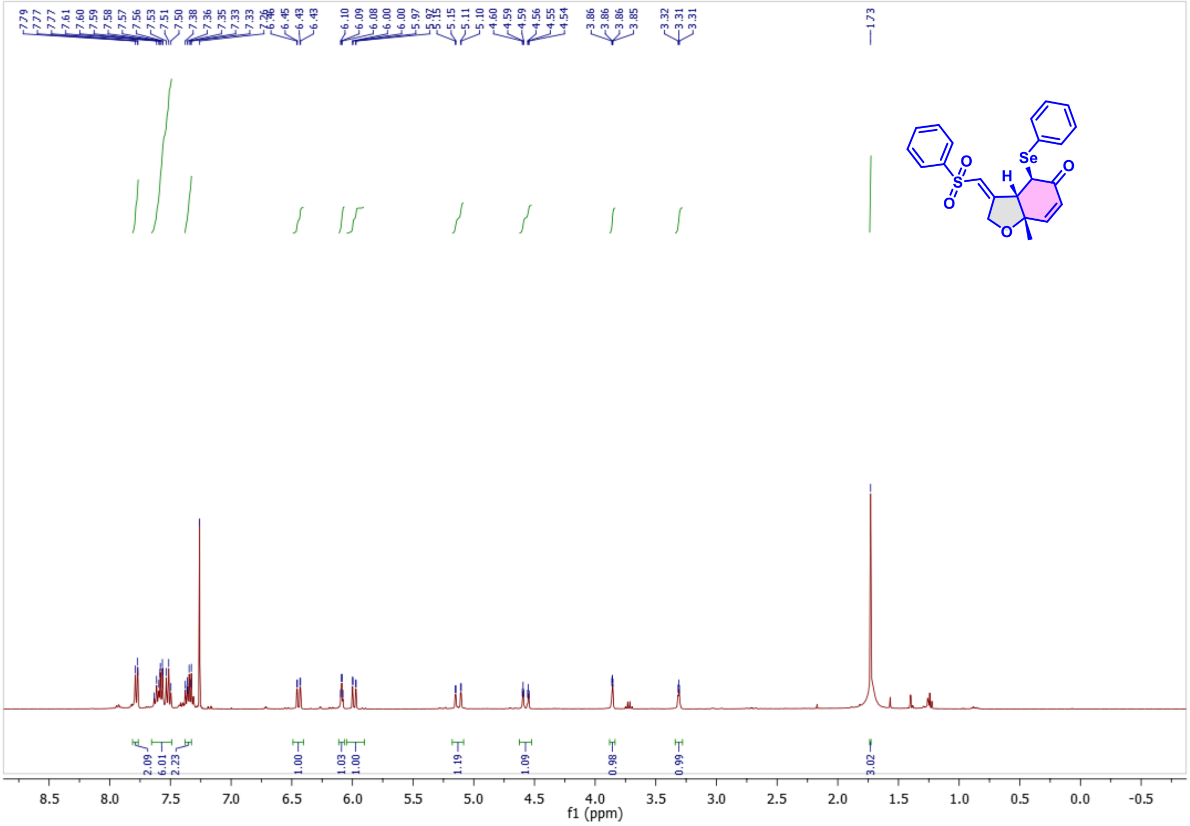

$^{13}C\{^{1}H\}$ NMR (100 MHz, $CDCl_3$) of 6i

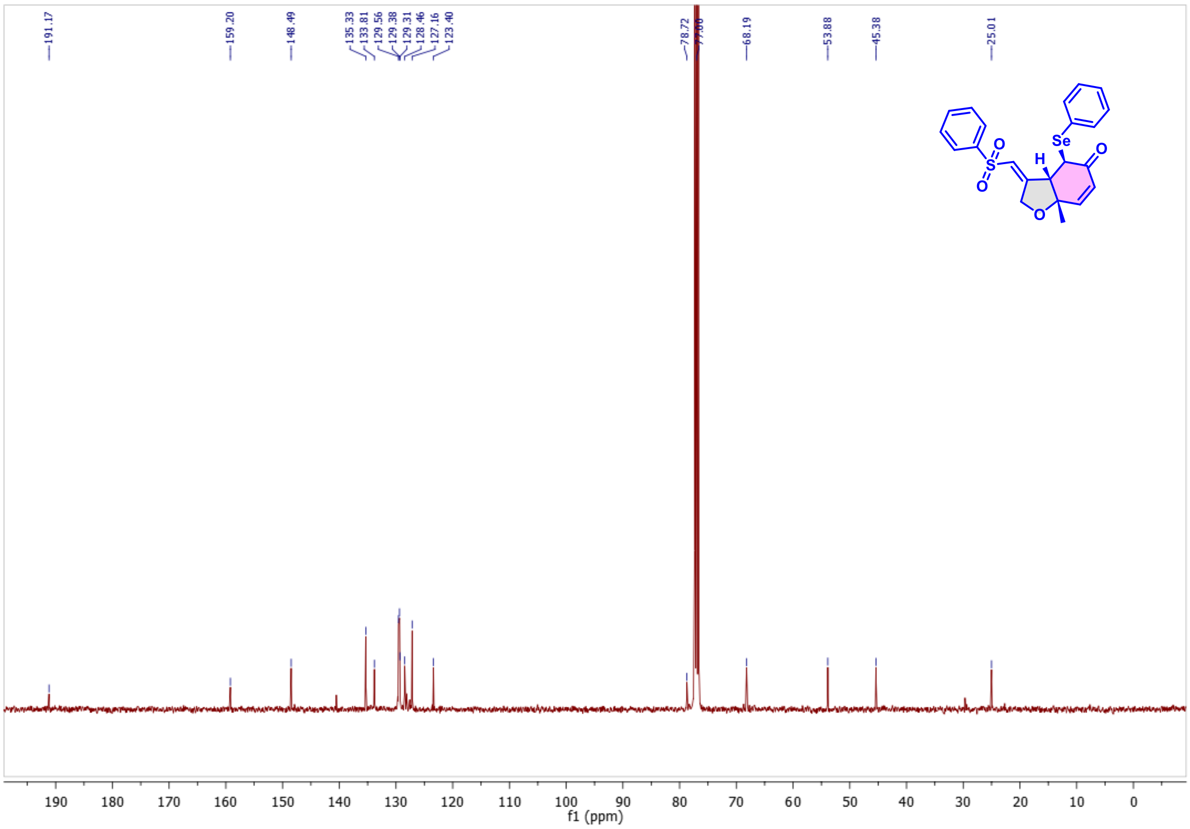

^{1}H NMR (400 MHz, CDCl$_3$) of 6j

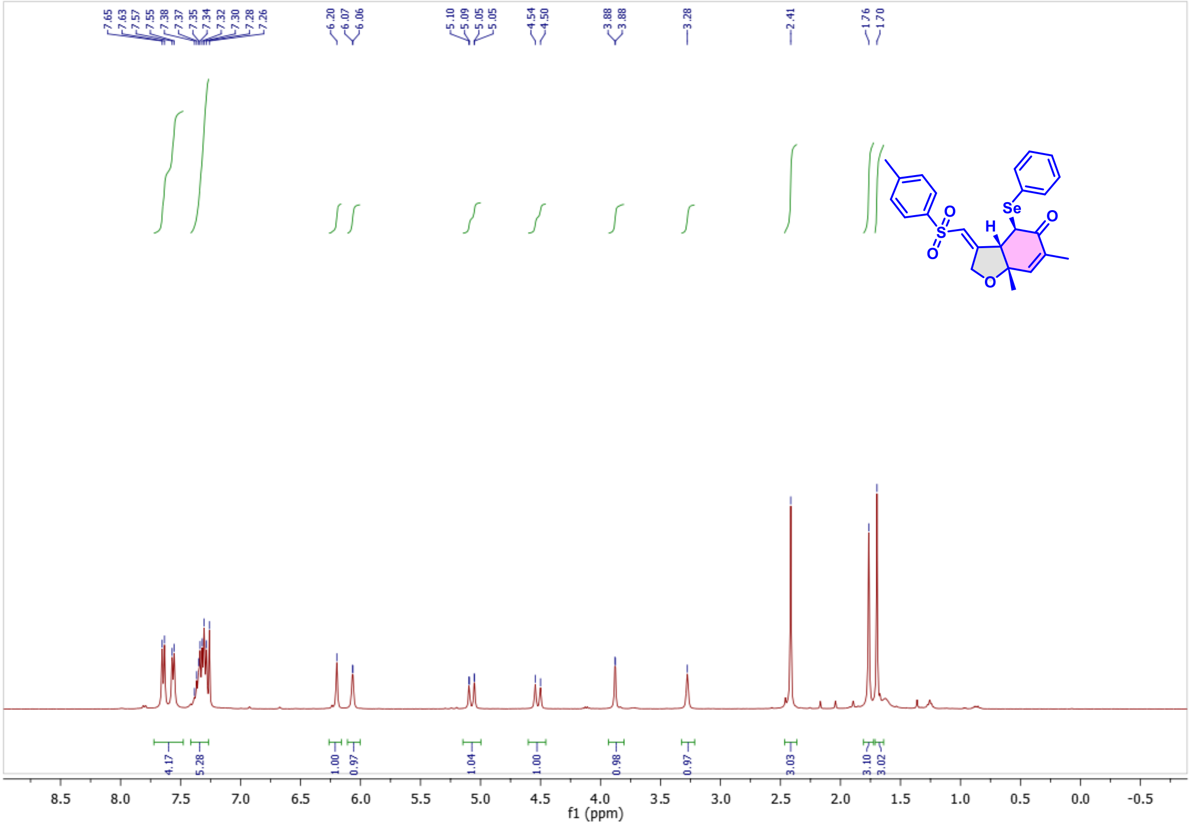

^{13}C{^{1}H} NMR (100 MHz, CDCl$_3$) of 6j

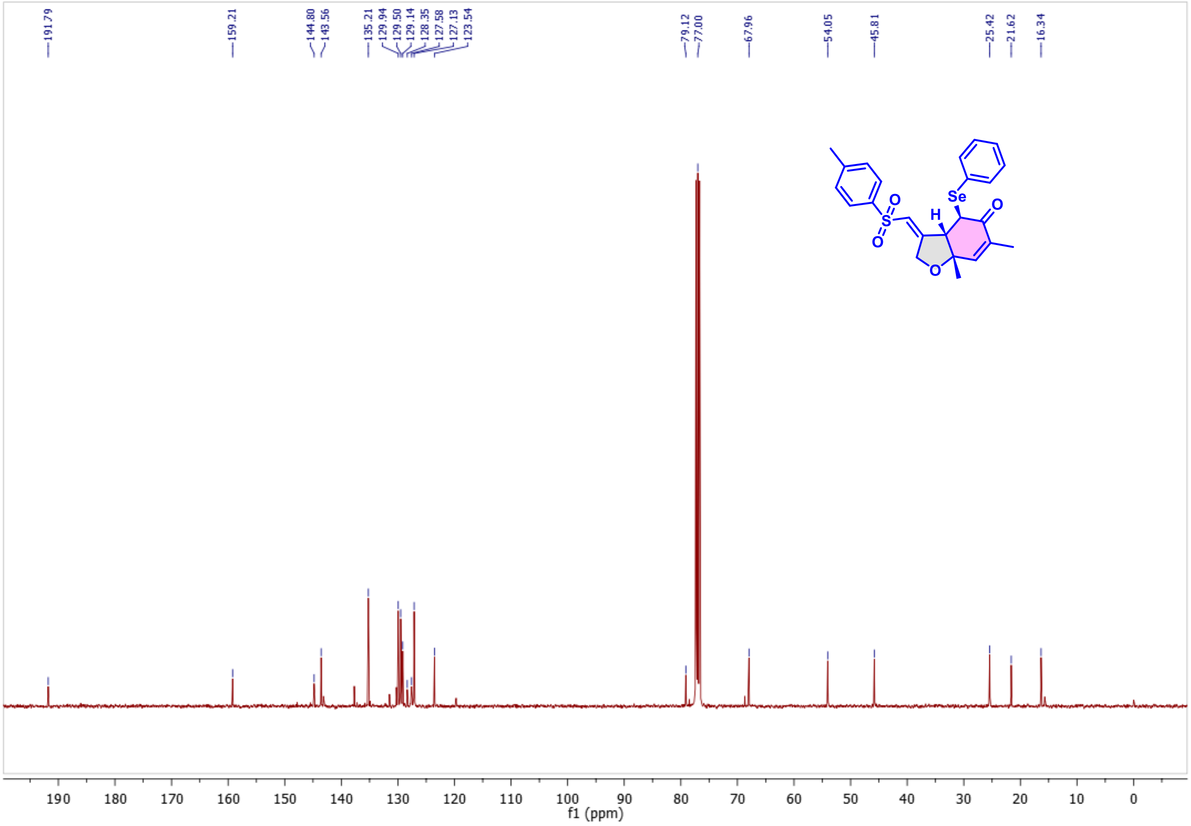

^{1}H NMR (400 MHz, $CDCl_3$) of 6k

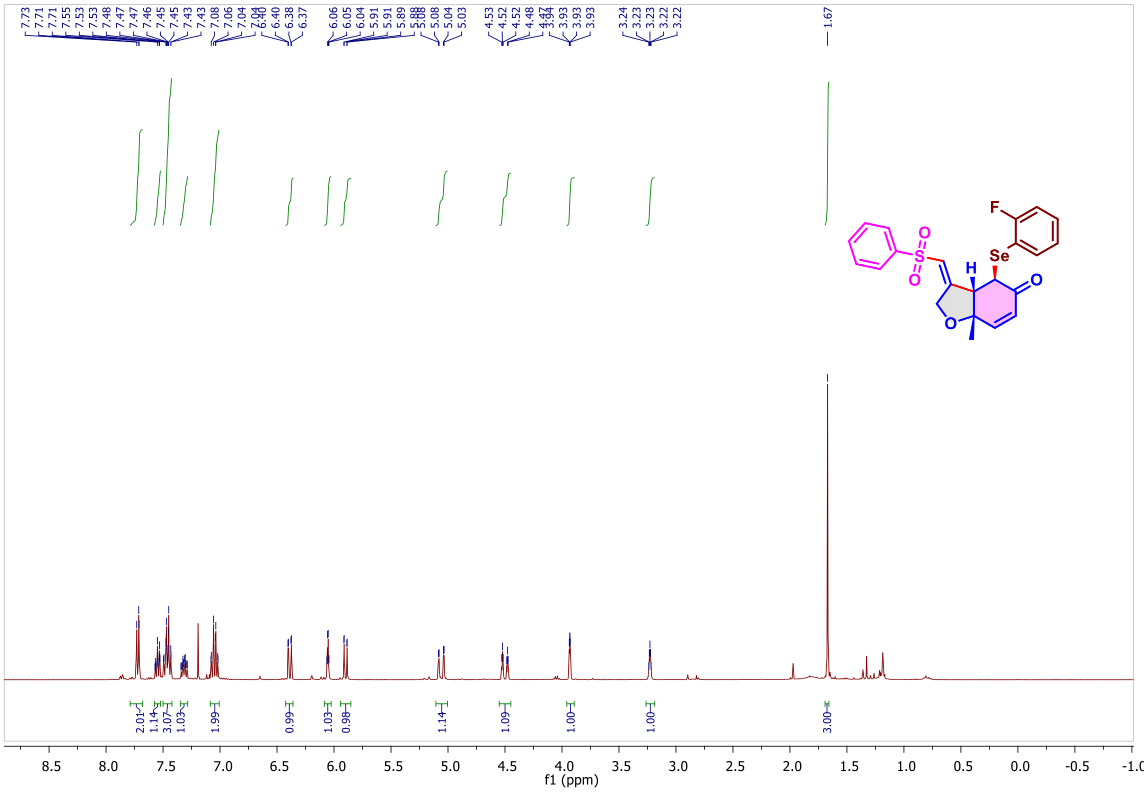

$^{13}C\{^{1}H\}$ NMR (100 MHz, $CDCl_3$) of 6k

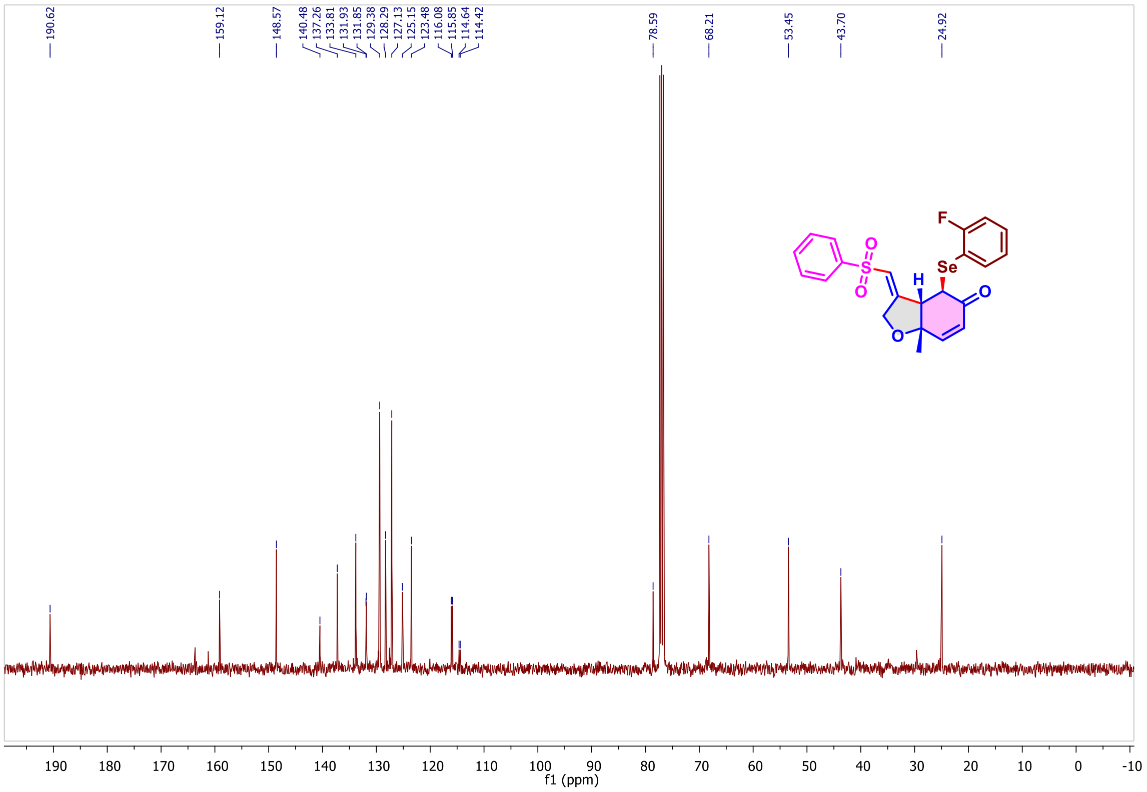

^{1}H NMR (500 MHz, $CDCl_3$) of 7

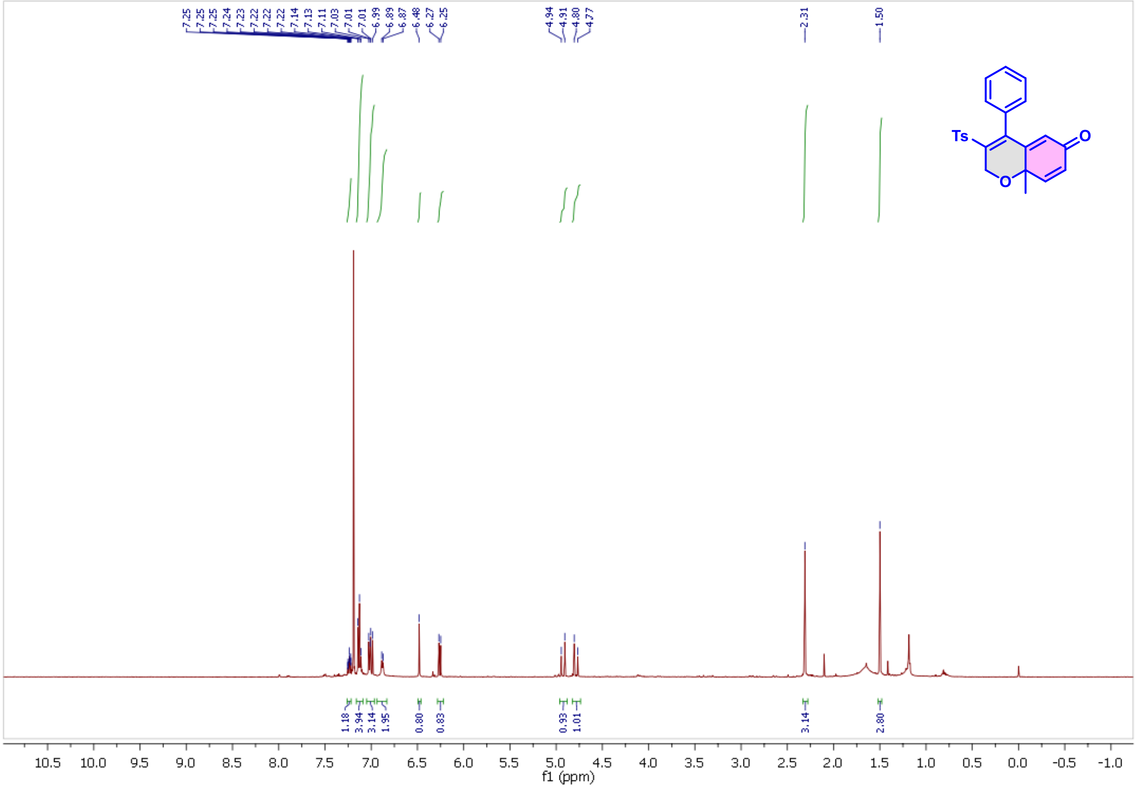

$^{13}C\{^{1}H\}$ NMR (100 MHz, $CDCl_3$) of 7

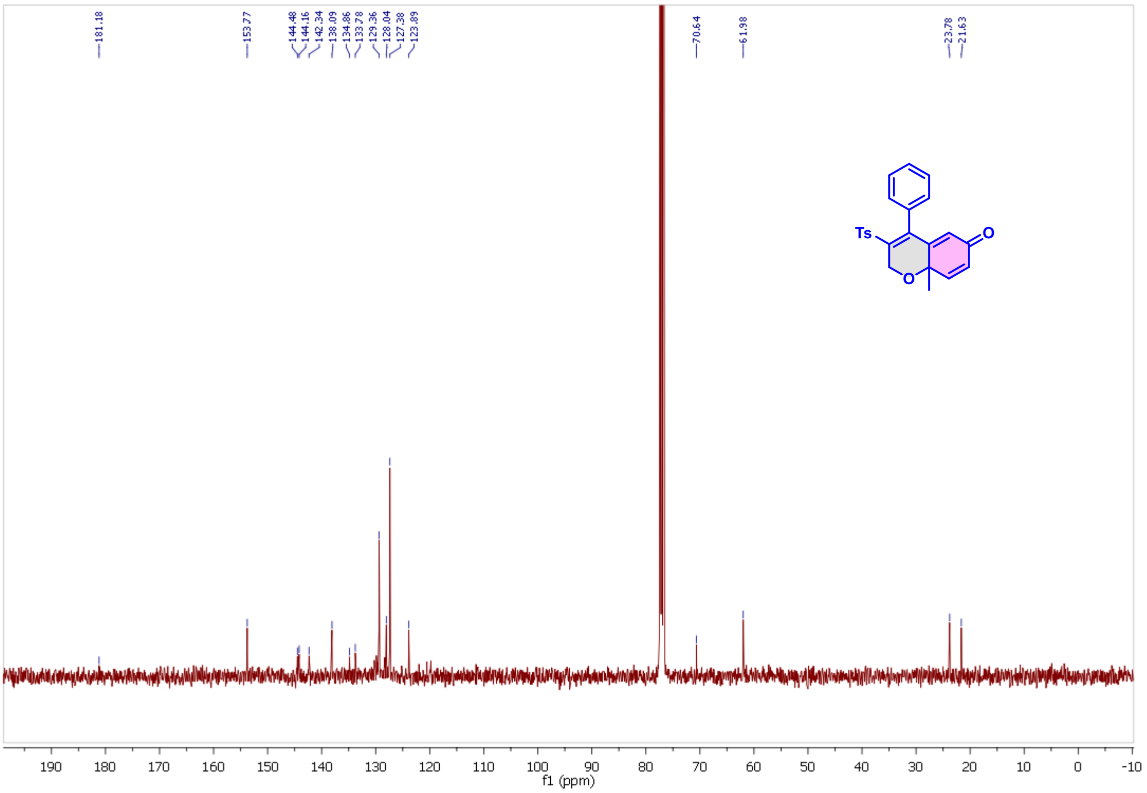

^{1}H NMR (400 MHz, $CDCl_3$) of 8

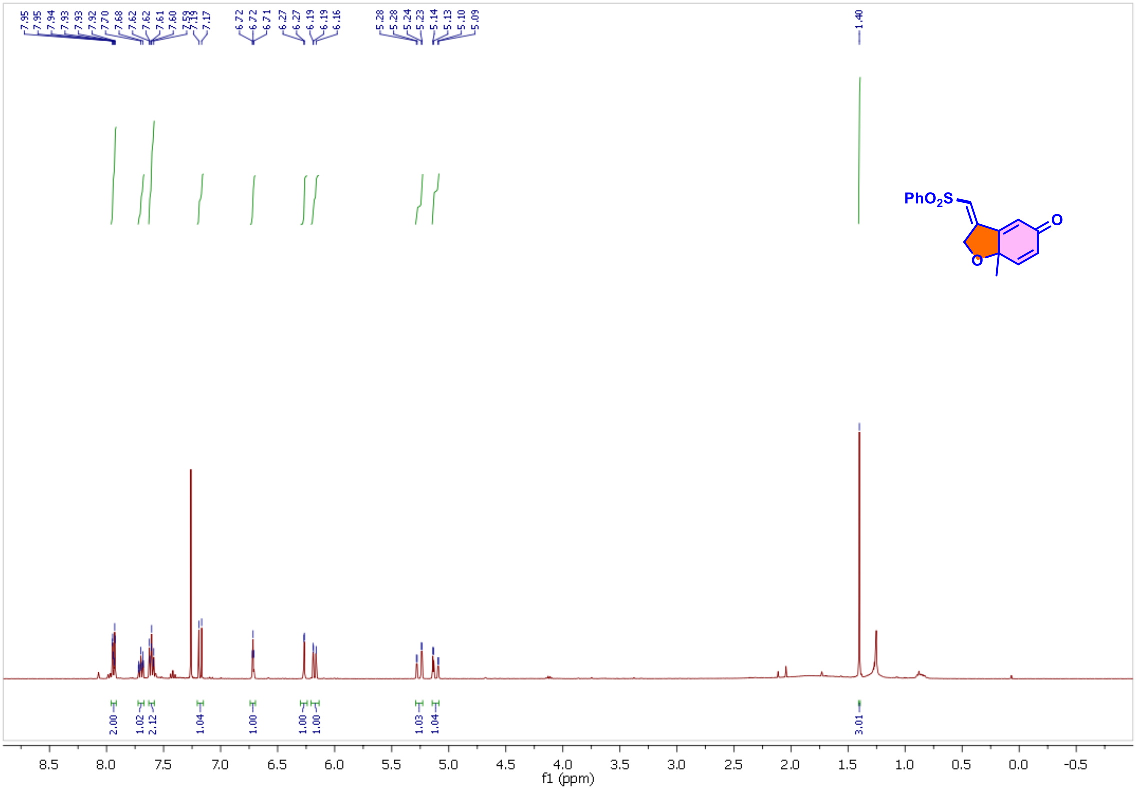

$^{13}C\{^{1}H\}$ NMR (100 MHz, $CDCl_3$) of 8

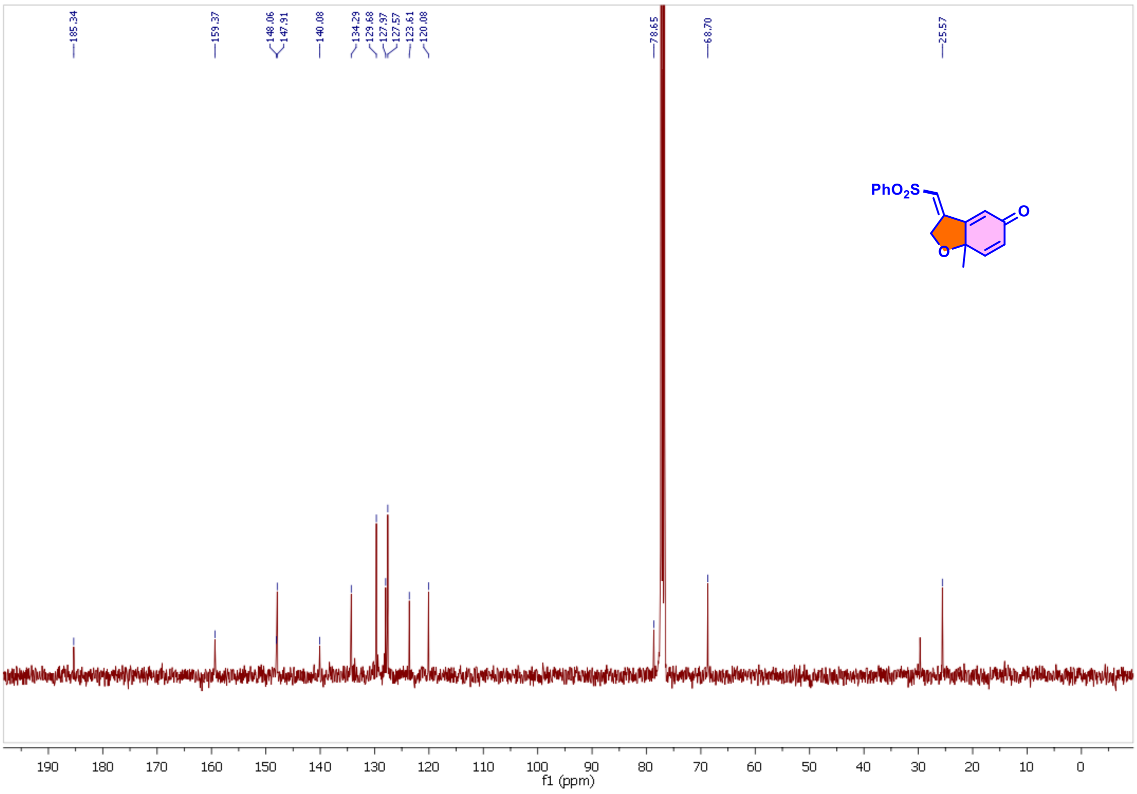

Supplement: Supplementary file 1 — ao3c03362_si_001.pdf [file ao3c03362_si_001.pdf]
